# Comorbidities in Obsessive-Compulsive Disorder Across the Lifespan: A Systematic Review and Meta-Analysis

**DOI:** 10.3389/fpsyt.2021.703701

**Published:** 2021-11-11

**Authors:** Eesha Sharma, Lavanya P. Sharma, Srinivas Balachander, Boyee Lin, Harshini Manohar, Puneet Khanna, Cynthia Lu, Kabir Garg, Tony Lazar Thomas, Anthony Chun Lam Au, Robert R. Selles, Davíð R. M. A. Højgaard, Gudmundur Skarphedinsson, S. Evelyn Stewart

**Affiliations:** ^1^Department of Child and Adolescent Psychiatry, National Institute of Mental Health and Neurosciences, Bangalore, India; ^2^Department of Psychiatry, National Institute of Mental Health and Neurosciences, Bangalore, India; ^3^Obsessive-Compulsive Disorders (OCD) Clinic, Accelerator Program for Discovery in Brain Disorders Using Stem Cells (ADBS), Department of Psychiatry, National Institute of Mental Health and Neurosciences, Bangalore, India; ^4^Department of Psychiatry, University of British Columbia, Vancouver, BC, Canada; ^5^Oxleas National Health Service (NHS) Foundation Trust, London, United Kingdom; ^6^Department of Child and Adolescent Psychiatry, Aarhus University Hospital, Psychiatry, Skejby, Denmark; ^7^Faculty of Psychology, University of Iceland, Reykjavik, Iceland

**Keywords:** obsessive-compulsive disorder, systematic review, meta-analysis, age, comorbidities

## Abstract

Comorbidities are seen with obsessive-compulsive disorder (OCD) across the lifespan. Neurodevelopmental comorbidities are common in young children, followed by mood, anxiety, and obsessive-compulsive related disorders (OCRDs) in children, adolescents and adults, and neurological and degenerative disorders in the elderly. Understanding comorbidity prevalence and patterns has clinical and research implications. We conducted a systematic review and meta-analysis on comorbidities in OCD across the lifespan, with the objective to, first, estimate age-wise pattern and prevalence of comorbidities with OCD and, second, to examine associations of demographic (age at assessment, gender distribution) and clinical characteristics (age of onset, illness severity) with comorbidities. Four electronic databases (PubMed, EMBASE, SCOPUS, and PsycINFO) were searched using predefined search terms for articles published between 1979 and 2020. Eligible studies, across age, reported original findings on comorbidities and had an OCD sample size of ≥100. We excluded studies that did not use standardised diagnostic assessments, or that excluded patients on the basis of comorbidity. We adhered to the Preferred Reporting Items for Systematic Reviews and Meta-Analyses guidelines. The review protocol has been registered on the International Prospective Register of Systematic Reviews. A comorbidity rate of 69% was found in a pooled sample of more than 15,000 individuals. Mood disorders (major depressive disorder), anxiety disorders (generalised anxiety disorder), neurodevelopmental disorders (NDDs) and OCRDs were the commonest comorbidities. Anxiety disorders prevailed in children, mood disorders in adults, whereas NDDs were similarly prevalent. Higher comorbidity with any psychiatric illness, NDDs, and severe mental disorders was seen in males, vs. females. Illness severity was inversely associated with rates for panic disorder, tic disorders, OCRDs, obsessive compulsive personality disorder, and anorexia nervosa. This systematic review and meta-analysis provides base rates for comorbidities in OCD across the lifespan. This has implications for comprehensive clinical evaluation and management planning. The high variability in comorbidity rates suggests the need for quality, multi-centric, large studies, using prospective designs.

**Systematic Review Registration:** Unique Identifier: CRD42020215904.

## Introduction

Obsessive-compulsive disorder (OCD) is a common and disabling psychiatric illness characterised by obsessions (repetitive, intrusive, unwanted and distressing thoughts, images, or urges) and compulsions (repetitive behaviours or mental acts that a person feels driven to perform in response to the obsessions, or according to rigid rules) (1, 2). Affecting children as young as 3–4 years old (3, 4), as well as the elderly over 70-years old (5), OCD incidence is generally highest during pre-adolescence (mean onset 11 years), and early adulthood (mean onset 23 years) (6, 7).

Various types of psychiatric disorders co-occur with OCD, including neurodevelopmental disorders (NDDs), mood disorders, anxiety disorders, severe mental illnesses (SMIs), and personality disorders (2, 8, 9). The understanding of comorbidities among psychiatric disorders is of immense clinical importance to inform primary diagnostic ascertainment, treatment planning and long-term management. Comorbidities also shed light on putative shared etiopathogenetic and neurobiological underpinnings.

Comorbidities in OCD have been examined, discussed and classified from different perspectives. Historically, relationships with anxiety disorders have been strongly emphasised, demonstrated by OCD's placement among anxiety disorders. However, with many OCD presentations extending beyond anxious states (e.g., disgust, “not-right” as the core negative valence state), the DSM-5 (1) has evolved to categorise OCD outside of the anxiety disorders and along with select phenomenologically similar comorbidities [Obsessive-Compulsive Related Disorders (OCRDs)]. Correspondingly, tic disorders have been examined as phenotypic markers for a homogeneous subgrouping among heterogeneous presentations of OCD (10–12), and their lifetime presence is a DSM-5 OCD specifier. Recent reviews have examined the epidemiological, clinical and psychopathological relationships between certain personality disorders, specifically schizotypal personality disorder (9) and obsessive-compulsive personality disorder (13), and OCD, suggesting the relevance of systematic clinical assessments and a need for further clinical, neurobiological and genetic enquiry in this area. Very few studies have systematically examined medical/neurological comorbidities in OCD. Risk of metabolic syndrome in OCD rises perhaps with the use of atypical antipsychotics as augmenting agents, with one study documenting more than 20% prevalence, much higher than general population estimates (14).

Reported rates of comorbid disorders with OCD have varied widely across published studies over the past 4 decades, even within similar socio-cultural backgrounds. For example, studies from the United States (USA) report varying lifetime prevalence rates of comorbid major depressive disorder (MDD) between 19% (15) and 66.8% (16) among adults with a primary diagnosis of OCD. Similar variations are observed in lifetime prevalence rates of other comorbid illnesses including anxiety disorders [22% (17)−56.3% (16)], tic disorders [8.7% (18)−31.6% (19)] and psychotic disorders [2.9% (20)−14.4% (21)]. Beyond variations related to chance and differences in study design, sampling strategy and measurement error, valid socio-demographic influences could plausibly underlie these observed variations. For example, differences in the reported comorbidity rate for MDD in OCD study populations of Klein et al. (15) and Mancebo et al. (16) may stem from differences in the mean age (34.4 vs. 40.1 years) and gender distribution (males 52.7 and 45%) within their samples. In comparing comorbid anxiety disorder rates, studies by Deacon et al. (17) and Mancebo et al. (16) differ with respect to age (35.8 vs. 40.1 year mean), age of onset of OCD (16.7 vs. 18.5 year mean), gender distribution (51 vs. 45%), and illness severity [Yale-Brown Obsessive Compulsive Scale (Y-BOCS) (22, 23) mean score 24 vs. 20.6]. Studies conducted with a primary aim to examine clinical variations by age, gender, etc., echo these observations (24–27), with differences particularly notable between paediatric samples (e.g., higher rates of NDDs like attention deficit hyperactivity disorder (ADHD) (28, 29) and adult samples (e.g., higher rates of mood disorders and anxiety disorders) (30, 31). Observed differences in comorbidity profiles across age possibly reflect variations in the relative etiopathological roles of genetic, neurobiological, and environmental factors (27, 32, 33). OCD presenting with comorbid illnesses tends to be more severe, with a more chronic course, and higher negative consequences on daily life functioning (34). It is also possible that changing diagnostic criteria and conceptualizations over time influence comorbidity rates.

Given the reported variations in comorbidity frequency across OCD samples, and the clinical and research implications of these differences, a critical evaluation of the extant literature is required. As a result, we report here a systematic review and meta-analysis of OCD comorbidities with the aim to estimate lifetime prevalence and age-related patterns of comorbidity (psychiatric, personality, and medical/neurological disorders) in OCD. A secondary aim was to examine the association between comorbidities and demographic (age at assessment, gender distribution) and clinical variables (age of onset, illness severity).

## Methods

### Search Strategy and Selection Criteria

We carried out a comprehensive search according to the Preferred Reporting Items for Systematic Reviews and Meta-Analyses (PRISMA) guidelines (35) across four databases–PubMed, Scopus, PsycINFO, and EMBASE. We looked for studies published between 1979 and October 2020 in keeping with the publication timelines for ICD-9 (36)/DSM-III (37) using a title search for terms “obsessive compulsive disorder,” “OCD,” “obsessive compulsive,” and “OC.” We looked at clinic or community based original studies published in English, meeting the following inclusion criteria: (i) OCD diagnosis meeting criteria on ICD-9/DSM-III or later versions, (ii) Diagnosis ascertained using standardised diagnostic interviews/instruments, (iii) OCD sample size ≥100, (iv) Reporting prevalence of comorbid disorders in frequency/percentages. Included studies were required to have at least 100 individuals with diagnosed OCD, in order to detect comorbidities at a least rate of 1%. Studies with comorbidity-based selection of participants were excluded. The protocol for this systematic review has been published on the International Prospective Register of Systematic Reviews (PROSPERO) (ID: CRD42020215904). Search results were imported into Covidence software (Veritas Health Innovation, n.d.) and all stages of screening and data extraction were completed on that platform. Each eligible study was screened, at both the title/abstract and full text screening stages, by two independent reviewers (from a team of ten reviewers). Conflicts were resolved by the lead reviewers (ES, LPS), who had an inter-rater agreement of more than 94%.

### Data Extraction and Assessment

Data from each study was independently extracted by two reviewers and then finalised by consensus. We extracted data on current and lifetime prevalence rates for all reported comorbid diagnoses. If a study did not state explicitly the examination of lifetime vs. current comorbidity, we subsumed reported rates under lifetime comorbidity. Other data extracted from each study included sample size, mean age at assessment (AAA), mean age of onset (AOO) of OCD, gender distribution (percentage of males), OCD illness severity rated on the Yale-Brown obsessive compulsive scale (Y-BOCS) (22, 23) or child Y-BOCS (CY-BOCS), respectively (38), country in which the study was carried out, time period of data collection, classification system used, diagnostic instrument used, study design, and recruitment source of participants [e.g. clinic-based (from treatment-seeking individuals presenting to the clinic) vs. community-based (from the general population), i.e., community based studies included all referrals from the community while the clinics only serve a selected proportion of all patients in the community]. Where reported, comorbidity data was extracted for both broad categories (e.g., mood disorders, anxiety disorders), as well as individual diagnoses [MDD, bipolar disorder, generalised anxiety disorder (GAD), etc.]. We combined prevalence of mania and bipolar disorder, and of schizophrenia and psychotic disorders. All other diagnoses were kept as reported.

### Statistical Analysis

All analyses were carried out with the R software (39), using the packages *metaphor* (40), *meta* (41), & *weight* (42). Meta-analysis of proportions, using random-effects models were carried out to estimate the pooled prevalence of each comorbidity. Only studies that explicitly reported rates of a particular comorbidity were included in the meta-analysis of that particular comorbidity. As most of the comorbidities had a prevalence rate of <20%, we used a double-arcsine transformation to ensure normality of the variance estimates. The pooled prevalence was then calculated using the DerSimionian-Laird inverse variance approach (43). This approach ensures that a study with a larger sample size is given more weight compared to a study with a smaller sample. The pooled prevalence estimates, along with the 95% confidence and prediction intervals are reported after back-transformation to percentages. The heterogeneity of the pooled estimates are reported using the *I*^2^ statistic and its *p*-value.

We used several methods for quality check of the meta-analysis (Supplementary File 2). Baujat plots, influence plots & the leave-one-out method of sensitivity analysis were used to look for studies with prevalence estimates that were outliers, along with their weightage. Data extraction from these studies were double-checked. If the study was found to be an outlier with a considerable effect on the pooled prevalence estimate for a particular comorbidity, then it was excluded from the analysis of only that comorbidity.

Subgroup analysis was done to look for the effect of age subgroups (adult: mean age ≥18 years; paediatric: mean age <18 years). Separate random-effects meta-analysis models were generated to estimate pooled prevalence estimates within these subgroups. Only those comorbidities that had at least 5 studies within the subgroups were meta-analysed. Subgroup effect is indicated using the *R*^2^ (indicates heritability explained by the moderator), *I*^2^ (residual heterogeneity of the meta-analysis after considering the moderator), and the Cochrane's Q_M_ statistic with its *p*-value. We also carried out meta-regression analysis with AAA, AOO, mean Y-BOCS/CY-BOCS total score (a measure of illness severity), and the percentage of male gender. We used the Q_M_ and corresponding *p-*value to test significance of the moderator.

## Results

### Study Selection

**Figure 1:**
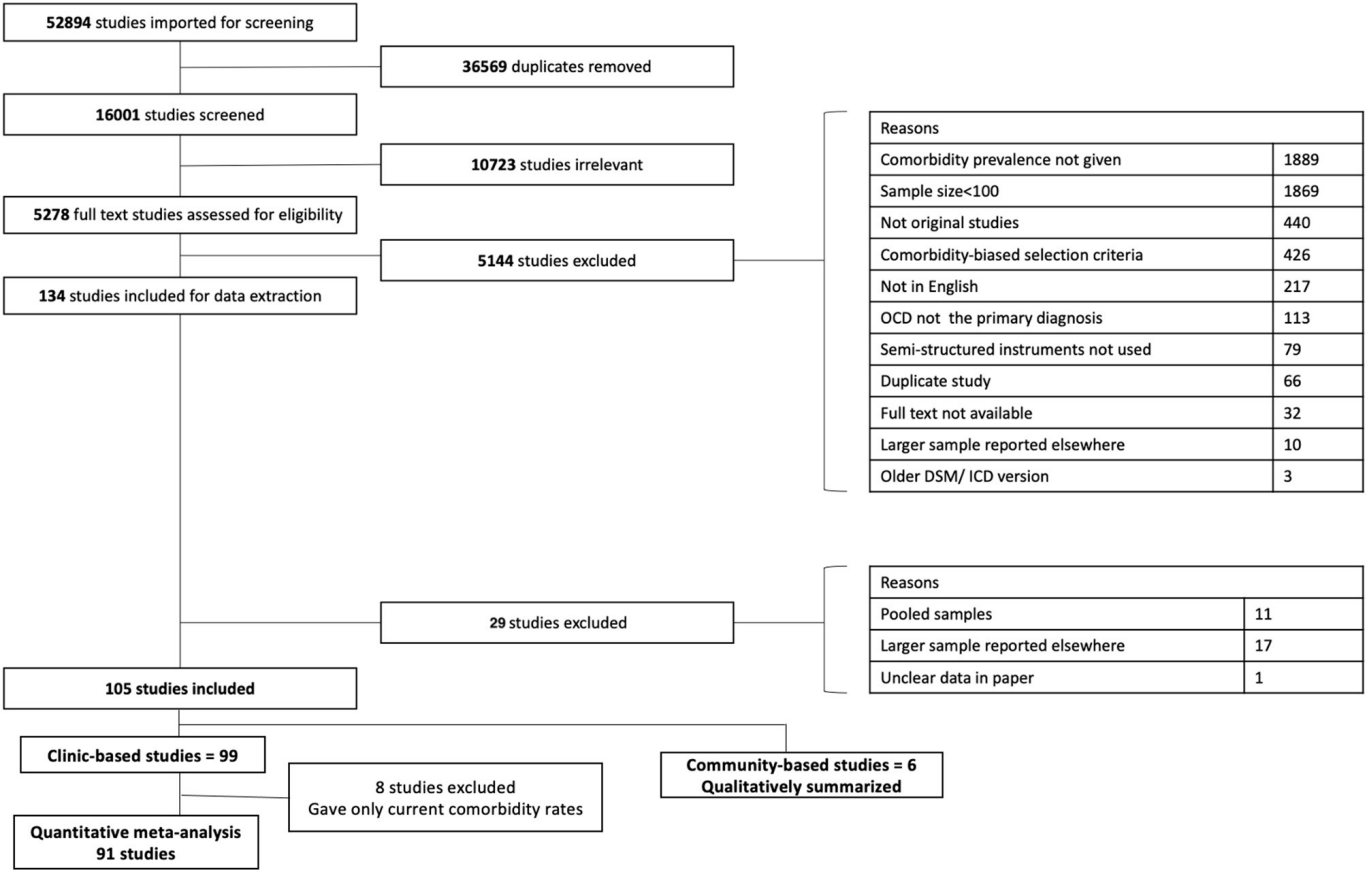
PRISMA flow diagram.

The PRISMA flow diagram is illustrated in Figure 1. From an initial set of 52,894 studies, 134 studies were short-listed for extraction following full-text screening. Following the extraction process, an additional 29 studies were excluded given concerns related to pooled samples (i.e., individual samples recruited for different studies that differed in selection criteria), and sample overlaps. In the latter case we retained comorbidity data from whichever reference reported on the largest sample from a given study, for each comorbid diagnosis. We chose to include comorbidities that had at least 5 studies reporting their prevalence within their samples. Among the 105 studies finally identified, 6 had community based recruitment. Pooling results from clinic- with community-based studies may suffer from biases given the conceptual and design differences in these two kinds of research. We have used only clinic-based studies, which reported lifetime comorbidities (*n* = 91), in our meta-analysis. Findings from community based studies have been qualitatively summarised separately. All selection decisions were reached by consensus.

### Quality Check

Supplementary File 2 (Figures 1.1–1.36) shows the steps for quality control, and studies that were excluded from the final analysis. Studies in the top right area of the Baujat plot (marked in red in the influence plots) were considered for exclusion.

### Characteristics of Clinic-Based Studies Included in Quantitative Meta-Analysis

Supplementary File 1 contains Tables (1–36) of all included studies for each comorbid disorder. The meta-analysis included 91 studies published over 3 decades (1992–2020). The pooled sample size of 15,808 individuals is marginally female predominant (51.5%); in both adult (females ~ 51.4%) and paediatric studies (females ~ 52.1%), in keeping with epidemiologic patterns (44, 45). Around 90% studies used the DSM-IV (46)/ICD-10 (47) or later diagnostic criteria to define OCD and comorbid disorders. There were 16 studies on paediatric OCD. Mean age varied between 11 and 15 years in the paediatric studies, and between 23 and 45 years in the adult studies. A majority of the studies reported on findings from cross-sectional assessments (66.3%). More than 30% of studies were conducted in the United States (USA), followed by Italy (10%) and The Netherlands (9%). Samples represented patients from highly developed nations such as Norway, Germany, Sweden, Australia, The Netherlands, and Denmark, as well as developing countries including South Africa and India (48). Less than 20% of the studies explicitly reported current comorbidities. We have pooled lifetime comorbidity rates in the meta-analysis.

### Pooled Prevalence Estimation in the Total Sample

**Figure 2:**
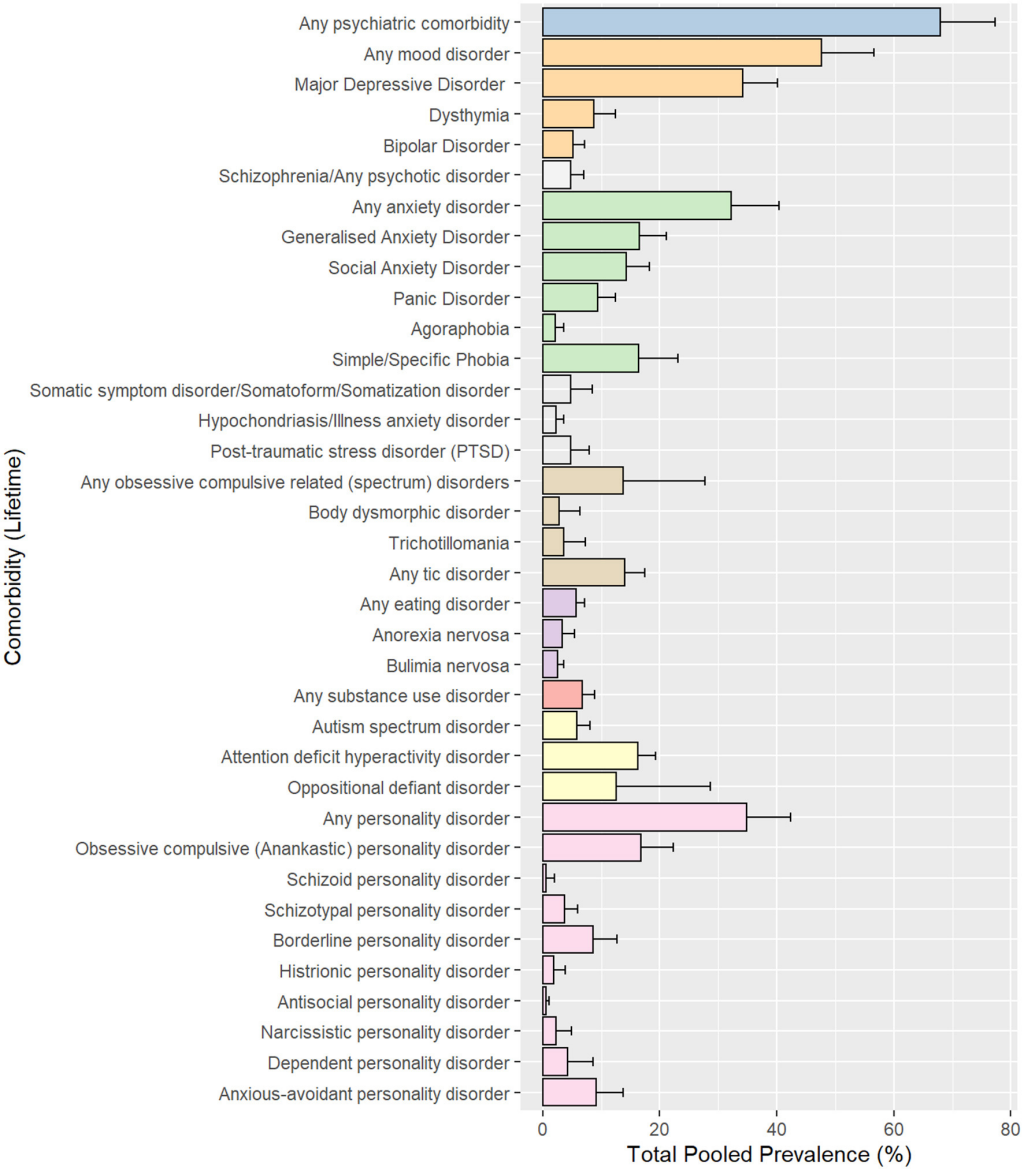
Pooled prevalence rates by comorbidity.

Figure 2.1.1–2.36.2 in Supplementary File 3 shows the results of the main meta-analysis for each disorder. Tables 1, 2, and Figure 2 show results of the main meta-analysis for each disorder, along with the number of studies and the total number of OCD subjects represented within the studies. In entirety, 69% (95% CI 59–78%) of the pooled sample had any psychiatric comorbidity. The most common comorbidity type, according to the highest pooled prevalence, was mood disorders, with a prevalence of 48% (95% CI 39–57%). The second most common comorbidity type was anxiety disorders, with a pooled prevalence of 32% (95% CI 24–40%). Other prominent comorbidities included ADHD [16% (95% CI 13–19%)], tic disorders [14% (95% CI 11–17%)], and OCRDs [14% (95% CI 4–28%)]. Autism spectrum disorders (ASD) [6% (95% CI 4–8%)] and oppositional defiant disorder (ODD) [12% (95% CI 2–29%)] were primarily reported in paediatric studies (only 1 study on adults reported ASD, while none reported ODD). The prevalence of personality disorders, from studies on adults, was also found to be high [35% (95% CI 28–42%)]. Among personality disorders, the most commonly reported was obsessive-compulsive personality disorder (OCPD) [17% (95% CI 12–22%)]. A very high level of heterogeneity (*I*^2^ > 90%) was found for nearly all the comorbidities examined.

**Table 1:** Pooled prevalence of psychiatric comorbidities in obsessive compulsive disorder.

**Comorbidity**	**Studies [N_**total**_ (n_**paediatric**_)]**	**Pooled sample size (*N*)**	**Pooled prevalence [95% CI, 95% PI]**	***I^**2**^***	**QE *p*-value**	**Pooled prevalence in adults (95% CI)**	**Pooled prevalence in children (95% CI)**	***I^**2**^* & QE *p*-value**	***R^**2**^* & Qm *P*-value**	**General population prevalence in adults (95% CI)**	**General population prevalence in children (95% CI)**
Any psychiatric comorbidity	23 (5)	6,272	69.3 [(59.4–78.3), (21.3–99.5)]	98.5	<0.001	70.8 (59.4–80.9)	63.6 (44.3–80.9)	98.1 (<0.001)	<0.001 (0.507)	13.04 (12.1–14.01) (49)	13.4 (11.3–15) (50)
**Mood disorders**											
Any mood disorder	21 (4)	6,187^∧^	47.7 [(38.8–56.7), (12.1–84.7)]	97.9	<0.001	53.8 (46.1–61.4)	NA	NA	NA	9.6 (8.5–10.7) (51)	
Major depressive disorder	43 (9)	9,909	35.4 [(29.2–41.8), (4–76.9)]	97.7	<0.001	40.8 (35.1–46.6)	17.1 (6.3–31.6)	97.1 (<0.001)	23.1 (0.001)	2.5 (2.2–2.8) (49)	1.3 (0.7–2.3) (50)
Dysthymia	17 (2)	4,924^∧^	9.7 [(6.3–13.7), (0.4–28.5)]	94.4	<0.001	10.4 (6.8–14.7)	NA	NA	NA	1.3 (1.1–1.6) (49)	
Bipolar disorder	22 (3)	6,158^∧^	5 [(3.2–7.3), (0–17.9)]	92.1	<0.001	4.9 (3.2–7.1)	NA	NA	NA	0.5 (0.4–0.6) (49)	1.8 (1.1–3.0) (52) (age range 7–21)
**Anxiety disorders**											
Any anxiety disorder	26 (9)	7,236	32.2 [(24.5–40.4), (2.6–74.5)]	98.1	<0.001	32.7 (22.7–43.7)	31.2 (21.1–42.4)	97.7 (<0.001)	<0.001 (0.847)	4.05 (3.4–4.8) (49) 12.9 (11.3–14.7) (51)	6.5 (4.7–9.1) (50)
Generalised anxiety disorder	29 (6)	7,658	17.2 [(12.8–22.1), (0.8–47.4)]	96.6	<0.001	15.02 (10.4–20.3)	26.6 (19.3–34.7)	95.2 (<0.001)	12.9 (0.029)	3.7 (0.1 SE) (53)	No comparable data
Social anxiety disorder	28 (6)	6,716	14.4 [(10.7–18.6), (0.7–39.8)]	95.3	<0.001	14.7 (10.1–19.9)	13.6 (10.3–17.1)	93.5 (<0.001)	<0.001 (0.778)	4 (0.1 SE) (54)	No comparable data
Panic disorder	25 (5)	6,180	9.4 [(6.4–12.8), (0.03–30.1)]	94.5	<0.001	10.3 (7.2–13.9)	6.1 (0.5–16.4)	93.6 (<0.001)	2.6 (0.224)	1.7 (0 SE) (55)	No comparable data
Agoraphobia	9 (2)	3,237^∧^	2.1 [(1.1–3.5), (0.06–6.5)]	78.6	<0.001	2.3 (1.1–3.8)	NA	NA	NA	1.4–1.5% (SE 0) (56)	
Simple/specific phobia	18 (5)	5,244	15.4 [(9.8–21.9), (0.05–47.9)]	97.3	<0.001	16.4 (9.6–24.7)	12.8 (4.7–23.9)	97.3 (<0.001)	<0.001 (0.596)	7.4 (0.1 SE) (57)	No comparable data
**Neurodevelopmental disorders**											
Attention deficit hyperactivity disorder	19 (13)	4,761	16.2 [(13.3–19.3), (6.2–29.5)]	85.8	<0.001	16.3 (12.1–21.02)	16.1 (12.05–20.6)	90.05 (<0.001)	<0.001 (0.932)	2.5 (2.1–3.1) (58)	3.4 (2.6–4.5) (50)
Any tic disorder	31 (12)	7,367	14 [(10.9–17.4), (1.6–35.4)]	93.7	<0.001	15.3 (10.8–20.5)	11.9 (9.1–14.9)	93.4 (<0.001)	0.15 (0.310)	No comparable data	Tourette's syndrome: 0.8 (0.4–1.5) (59) Transient Tic Disorder: 3 (1.6–5.6) (59)
Autism spectrum disorders	7 (6)	1,497^#^	5.9 [(4–8.05), (2.4–10.6)]	54.6	0.051	NA	5.8 (3.47–8.75)	NA	NA	0.4 (0.3–0.4) (49)	2.8–94/10,000 (~0.03–0.9) (60)
**Obsessive compulsive related disorders (OCRDs)**											
Any OCRD	5 (1)	1,624	13.7 [(4.1–27.7), (0–51.6)]	97.8	<0.001	17.5 (5.7–33.9)	NA	NA	NA	No comparable data	No comparable data
Body dysmorphic disorder	6 (1)	1,561	2.8 [(0.6–6.4), (0–12.9)]	87.5	<0.001	3.1 (0.4–7.8)	NA	NA	NA	1.9 (1.4–2.7) (61)	No comparable data
Trichotillomania	5 (2)	1,102	3.6 [(1.1–7.3), (0–13.6)]	85.7	<0.001	5.3 (1.1–12.2)	NA	NA	NA	No comparable data	No comparable data
**Eating disorders**											
Any eating disorder	17 (3)	5,298	5.6 [(4.3–7.1), (1.7–11.6)]	75.7	<0.001	5.3 (3.8–7.04)	NA	NA	NA	1.01 (0.5–1.9) (62)	No comparable data
Anorexia nervosa	9 (4)	1,790	3.2 [(1.6–5.4), (0.01–10.6)]	79.2	<0.001	3.8 (1.9–6.3)	NA	NA	NA	0.2 (0.1–0.4) (62)	No comparable data
Bulimia nervosa	7 (1)	1,418	2.6 [(1.7–3.6), (1.7–3.6)]	0.00	0.438	2.8 (1.8–3.8)	NA	NA	NA	0.8 (0.6–1.1) (62)	No comparable data
**Somatic symptom and related disorders**											
Somatic symptom disorder^+^	11 (0)	3,839	5.3 [(2.5–9.1), (0–21.2)]	94.9	<0.001	5.3 (2.5–9.1)	NA	NA	NA	No comparable data	No comparable data
Illness anxiety disorder^++^	7 (0)	1,971	2.2 [(1.1–3.6), (0.2–5.8)]	65.4	0.008	2.2 (1.1–3.6)	NA	NA	NA	No comparable data	No comparable data
**Other disorders**											
Any substance use disorder	17 (1)	5,334^∧^	6.7 [(4.9–8.8), (1.3–15.7)]	85.8	<0.001	7.2 (5.4–9.3)	NA	NA	NA	10.7 (9.2–12.4) (51)	No comparable data
Post-traumatic stress disorder	16 (2)	4,167^∧^	5.1 [(2.5–8.4), (0–21.9)]	94.2	<0.001	5.9 (3.04–9.7)	NA	NA	NA	3.9 (SE 0.1) (63)	No comparable data
Schizophrenia/any psychotic disorder	13 (2)	4,642^∧^	4.5 [(2.7–6.7), (0.1–13.7)]	89.7	<0.001	3.5 (2.1–5.2)	NA	NA	NA	1 (0.7–1.2) (64)	No comparable data
Oppositional defiant disorder	7 (7)	1,095^#^	12.5 [(2.3–28.7), (0–64.3)]	97.8	<0.001	NA	12.5 (2.3–28.7)	NA	NA	Not applicable	3.6 (2.8–4.7) (50)

*I^2^, Residual heterogeneity; QE & p-value, Significance of residual heterogeneity; 95% CI, 95% Confidence Intervals; 95% PI, 95% Prediction intervals; R^2^, Explained heterogeneity; QM & p-value, Significance of heterogeneity explained by the moderator; NA, Not applicable;*

∧ *Pooled prevalence not calculated separately for paediatric studies since they numbered <5;*

# *Pooled prevalence not calculated separately for adult studies since they numbered <5;*

+ *Includes somatoform/somatization disorders;*

++ *Includes hypochondriasis*.

**Table 2:** Pooled prevalence of personality disorder comorbidities among adults with obsessive compulsive disorder.

**Comorbidity**	**No. of Studies**	**Pooled sample size**	**Pooled prevalence [95% CI, 95% PI]**	***I^**2**^***	**QE *p*-value**	**General population prevalence (95% CI)**
Any personality disorder	7	1,970	34.9 [(27.8–42.3), (17.02–55.3)]	91.2	<0.001	7.8 (6.1–9.5) (68)
Obsessive compulsive personality disorder	12	2,518	16.8 [(11.8–22.4), (3.4–37.2)]	91.6	<0.001	3.2 (2.4, 4.1) (68)
Anxious avoidant personality disorder	9	2,076	9.2 [(5.4–13.8), (0.7–25.1)]	90.6	<0.001	2.7 (1.9, 3.7) (68)
Borderline personality disorder	9	1,997	8.6 [(5.3–12.6), (1.03–22.1)]	87.5	<0.001	1.8 (1.2, 2.5) (68)
Dependent personality disorder	8	1,936	4.3 [(1.3–8.6), (0–20.05)]	93.2	<0.001	0.8 (0.5, 1.3) (68)
Schizotypal personality disorder	11	2,324	3.7 [(1.9–5.9), (0.01–12.2)]	82.9	<0.001	0.8 (0.5, 1.1) (68)
Narcissistic personality disorder	7	1,775	2.3 [(0.6–4.9), (0–11.2)]	88.2	<0.001	1.9 (0.1, 5.6) (68)
Histrionic personality disorder	7	1,775	1.9 [(0.5–3.8), (0–7.6)]	80.8	<0.001	0.6 (0.4, 0.9) (68)
Antisocial personality disorder	8	1,885	0.6 [(0.2–1.1), (0.1–1.3)]	8.6	0.363	1.4 (0.8, 2.3) (68)
Schizoid personality disorder	8	1,936	0.6 [(0–1.9), (0–5.5)]	83.0	<0.001	1.1 (0.7, 1.5) (68)

*I^2^, Residual heterogeneity; QE, Cochran's Q statistic for significance of the residual heterogeneity; 95% CI, 95% Confidence Intervals; 95% PI, 95% Prediction intervals*.

### Subgroup Analysis

**Figure 3:**
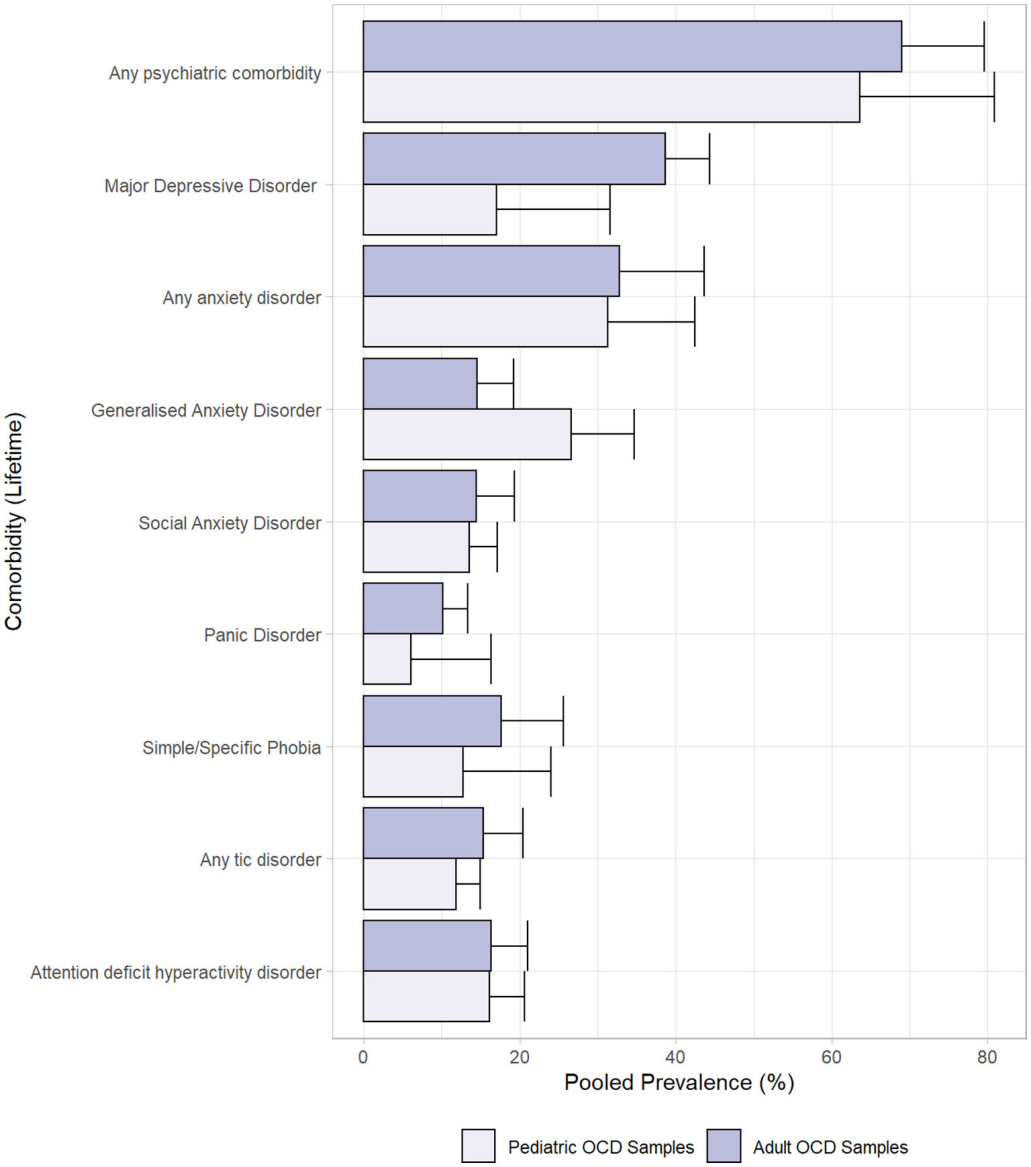
Pooled prevalence rates by comorbidity in adult and paediatric subgroups.

Figure 3 and Table 1 show the comparison of pooled prevalence estimates between the adult vs. paediatric subgroups. Pooled prevalence for any psychiatric comorbidity was similar in adult studies and paediatric studies (71 and 64%, *p* = 0.51). Anxiety disorders were the most common comorbidity in the paediatric subgroup whereas mood disorders were the most common in the adult subgroup. Significant differences between the subgroups were found for MDD (41 vs. 17%, *p* < 0.001), and GAD (15 vs. 27%, *p* = 0.029), whereas NDDs, specifically tic disorders and ADHD showed similar pooled prevalence across adult and paediatric subgroups.

**Figure 4:**
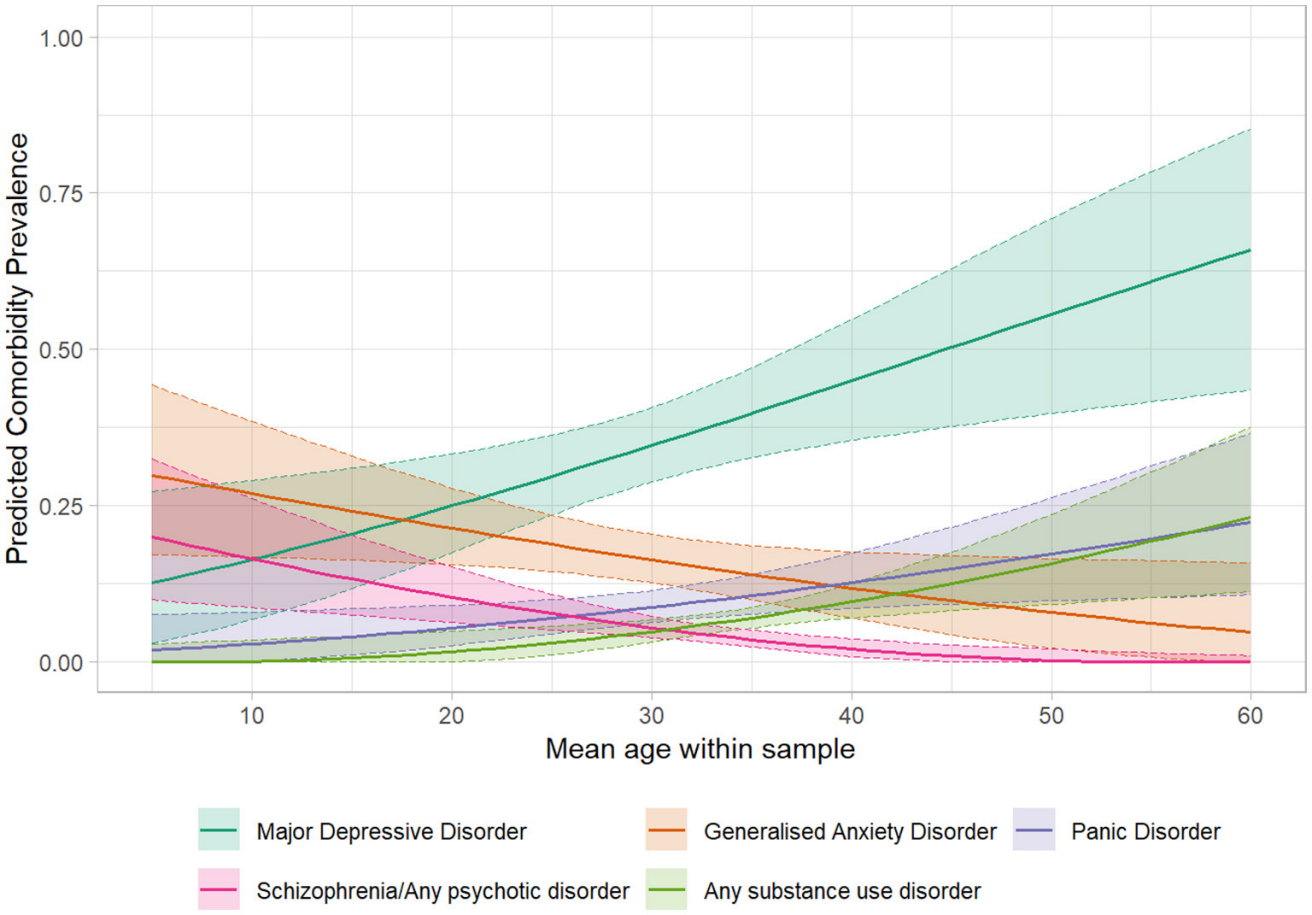
Significant meta-regressions by mean age at assessment of samples. Rates of MDD, Substance use & Panic Disorder are higher in older samples, whereas rates of Schizophrenia/Psychosis & GAD are higher in younger samples.

**Figure 5:**
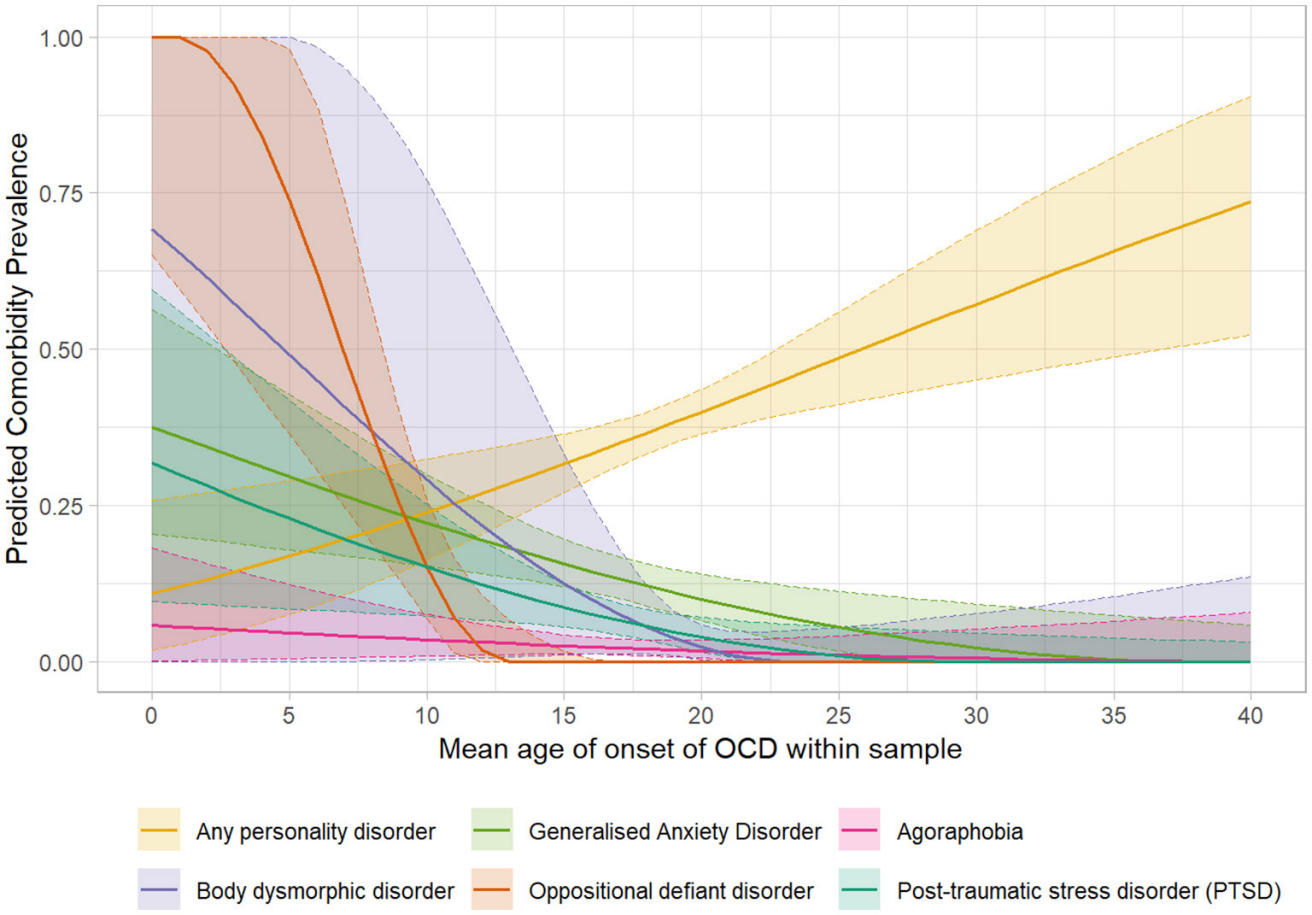
Significant meta-regressions by mean age of onset of samples. Rates of GAD, Agoraphobia, ODD, PTSD & BDD are higher in samples with earlier mean age of onset, while rates of Personality Disorders are higher in samples with later age of onset.

**Figure 6:**
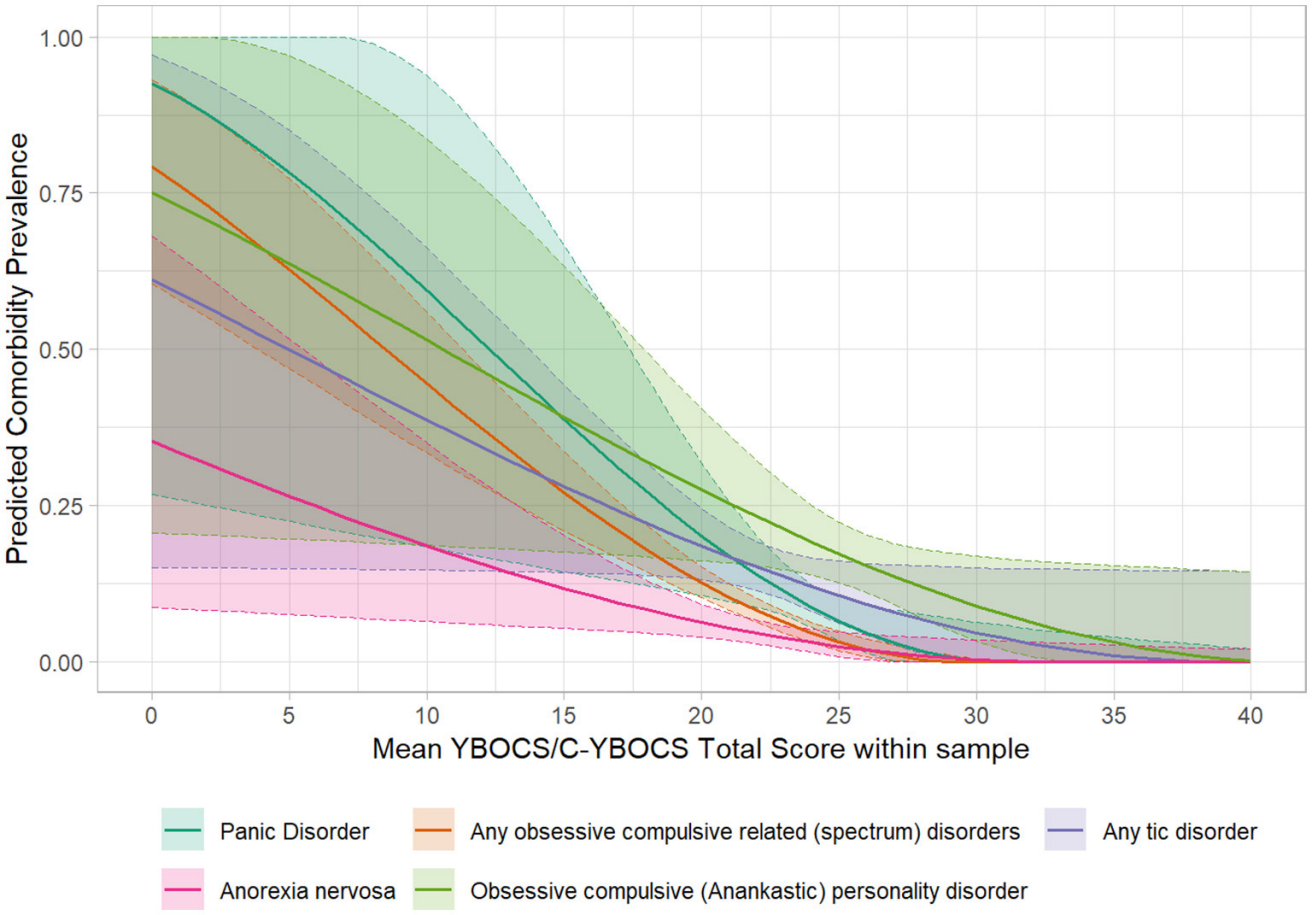
Significant meta-regressions by mean Y-BOCS/CY-BOCS total score in the samples. The prevalence rates of Panic Disorder, OCRDs, Tic Disorder, Anorexia Nervosa & OCPD are lower in samples with higher illness severity.

**Figure 7:**
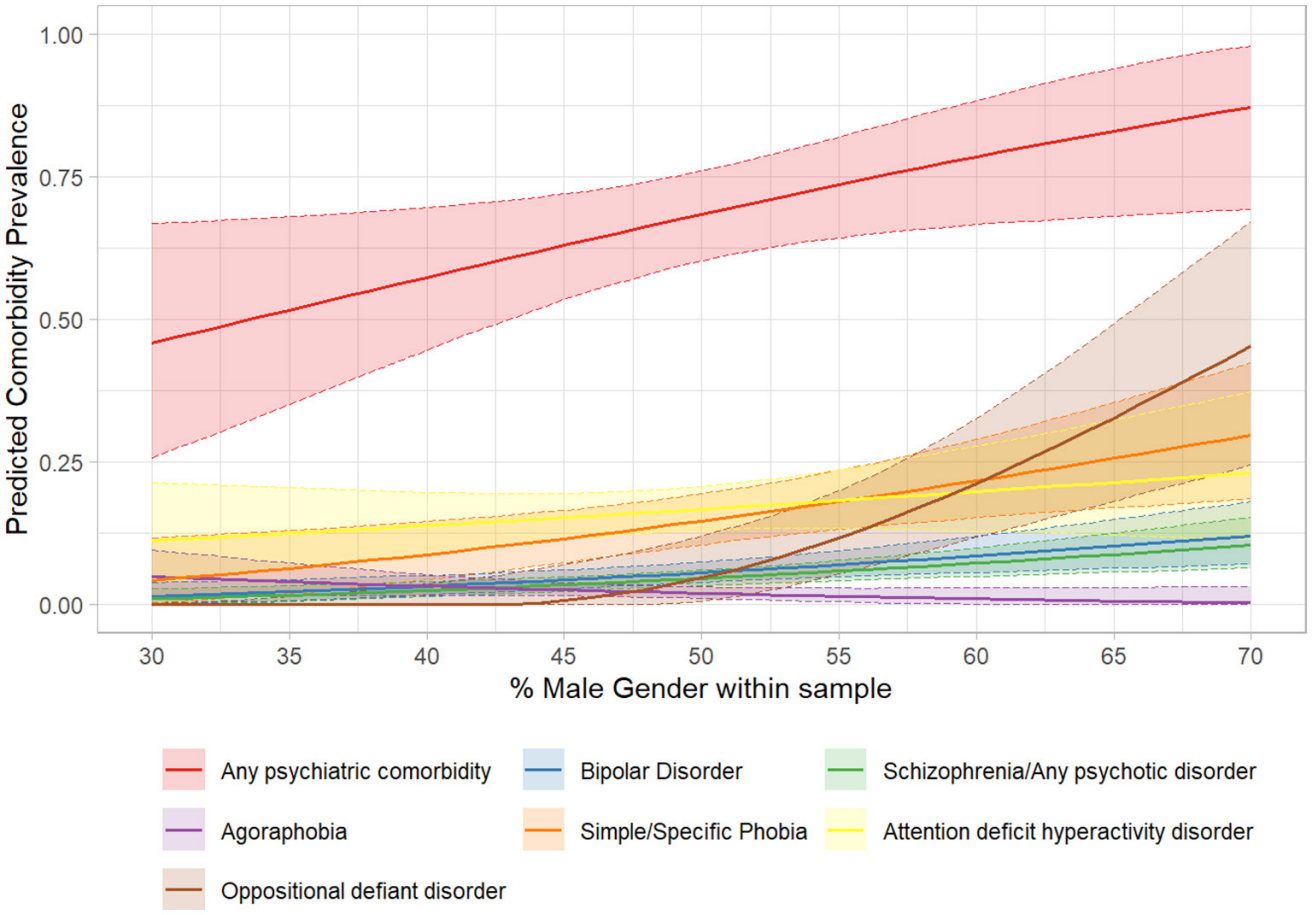
Significant meta-regressions by percentage of males within samples. The prevalence rates of Any Psychiatric Illness & ODD are higher in samples with higher percentage of males. Bipolar Disorder, Schizophrenia/Psychosis & ADHD show a similar trend.

Figure 3.1–3.36 in Supplementary File 4 shows figures depicting the results of the meta-regressions, for each comorbidity. Significant associations are presented in Figures 4–7. Significant effects of AAA were found for MDD (higher AAA, higher MDD), GAD (higher AAA, lower GAD), panic disorder (higher AAA, higher panic disorder), psychotic disorder (higher AAA, lower psychotic disorders), and substance use disorders (SUDs) (higher AAA, higher SUD)]. Significant effects of AOO were found for GAD (lower AAO, higher GAD), post-traumatic stress disorder (PTSD) (lower AOO, higher PTSD), agoraphobia (lower AOO, higher agoraphobia), body dysmorphic disorder (BDD) (lower AOO, higher BDD), ODD (lower AOO, higher ODD), and personality disorders (higher AOO, higher personality disorders). Y-BOCS total score, representing OCD severity, was significantly associated with lower rates of comorbid panic disorder (higher Y-BOCS, lower comorbid panic), tic disorders (higher Y-BOCS, lower comorbid tics), any OCRDs (higher Y-BOCS, lower OCRDs), anorexia nervosa (higher Y-BOCS, lower anorexia nervosa), and OCPD (higher Y-BOCS, lower OCPD). Percentage of male gender within the sample was associated with higher prevalence of any psychiatric comorbidity, bipolar disorder, psychosis, agoraphobia, specific phobia, ADHD, and ODD.

### Comorbidity Patterns in Community-Based Studies

Six community-based studies (Table 3) met the selection criteria of this meta-analysis. These studies were published between 1988 and 2020. All these studies involved cross-sectional assessments on adult participants. These studies were based on five community surveys—Epidemiologic Catchment Area study (ECA) in the USA (65, 71), the Singapore Mental Health Studies 2010 and 2016 (69, 70), a population-based study from Iran (68), and the British National Psychiatry Morbidity Survey 2000 (66). A direct comparison between epidemiologic/ community- and clinic-based samples was not made, due to the stark difference in numbers, as also, more fundamentally, study conceptualisation and design. Reports from the ECA survey highlight the instability in primary and comorbid diagnoses in community surveys (65, 71), perhaps influenced by recall bias and reporting variations due to stigma (69). The ECA studies (65, 71) reported comorbidities including mood disorders, anxiety disorders, substance use disorders, schizophrenia, and schizophreniform disorders. Those with a stable diagnosis across waves 1 and 2 had lower age of onset and higher rates of depressive and anxiety disorders, substance use and schizophrenia (71). The Singapore national mental health surveys (69, 70) described comorbidity patterns of psychiatric disorders as well as medical disorders. The study from Iran (68) found OCD to be highly comorbid with depressive and anxiety disorders; they also reported prevalence of comorbid severe mental illness (bipolar disorder, schizophrenia) and epilepsy. The British study (66, 67) described various substance use comorbidity patterns and a screening report on personality disorders with OCD. Overall, these studies reported prevalence of depressive disorders, the most common comorbidity, ranging from 14 to 43%. Rates of anxiety disorders varied (Specific phobias: 5–46%, GAD: 5–31%, Social phobia: 8–25%, Panic disorder: 6–26%). The prevalence of schizophrenia was between 2 and 3%, except for a higher prevalence of 17.9% reported in wave I of the ECA study (65). However, this study had a substantially lower number of individuals (<12%) with a “stable” diagnosis of OCD (71). The rates of alcohol and substance use disorders varied widely across studies.

**Table 3:** Prevalence of comorbidities in obsessive-compulsive disorder in community surveys.

**Study**	**Country**	**Total sample size**	**No. of individuals with OCD identified**	**OCD Sample characteristics**	**Diagnostic instrument used**	**Comorbidities**			
						**Depressive disorders**	**Anxiety disorders**	**Severe mental illness**	**Others**
Karno et al. (65) Epidemiologic catchment area study	USA	18,572	468	Adult	Diagnostic interview schedule (DIS)	31.7%	Phobia 46.5% Panic disorder 13.8%	Schizophrenia 12.2%	Alcohol abuse/dependence: 24.1% Other drug abuse/dependence: 17.6%
Torres et al. (66, 67) British national psychiatric morbidity survey of 2000	UK	8,580	114	Adult (16–74 years), 35% males	Clinical interview schedule–revised (CIS-R) & Structured clinical interview for axis-II disorders (SCID-II)	36.8%	GAD 31.4% Agoraphobia/Panic disorder 22.1% Social phobia 17.3% Specific phobia 15.1%	Schizophrenia 2.6%	Alcohol dependence 20.2% Any drug dependence 13.5% Cannabis dependence 11.5% Personality disorders (screening criteria) 74%
Mohammadi et al. (68)	Iran	25,180	444	Adult (Mean age 37.2+/−6.6 years) 50.3% males	Schedule for affective disorders and schizophrenia	14%	Simple phobia 10.8% Social phobia 8.1% Panic disorder 6.5% GAD 5.2%	Bipolar disorder I 0.5% Bipolar II 2.5% Schizophrenia 2.3%	Epilepsy 6.8% PTSD 1.1% Somatoform disorder 0.9%
Subramaniam et al. (69) Singapore mental health study 2010	Singapore	6,616	230	Adult	Composite international diagnostic interview version 3.0 (CIDI 3.0)	26.8%	GAD 12.3%	Bipolar disorder 10.5%	Alcohol dependence 2.1% Physical comorbidity 51.6% Chronic pain 21.8% Respiratory conditions 17.4% Hypertension 10.8%
Subramaniam et al. (70) Singapore mental health study 2016	Singapore	6,126	217	Adult 49.6% males	CIDI 3.0	28.2%	GAD 9.9%	Bipolar disorder 12.2%	Alcohol dependence: 0.9% Nicotine dependence: 4.6% Physical comorbidity^*^: 52.3% Chronic pain 33.2% Hyperlipidaemia 15.3% Hypertension 13.2%

∧ *Comorbidity characteristics have been further reported in another ECA paper (71) that compares and estimates the impact of diagnostic stability on comorbidity patters*.

* *For e.g., hypertension, hyperlipidemia, diabetes, asthma, chronic pain, cardiovascular disease, ulcer, thyroid disease, cancer*.

### GRADE Based Evaluation of the Pooled Prevalence Estimates for Comorbidities

The GRADE (Grading of Recommendations Assessment, Development and Evaluation) approach was primarily designed for clinical practice guidelines. No formal guidelines exist for applying these to systematic reviews of prevalence (72). We have adapted Iorio's et al. (73) suggestions for operationalization of the GRADE approach for prognosis estimates. The Tables 1, 2 in Supplementary File 5 presents the GRADE scoring for pooled estimation of each comorbid condition. The findings are summarised below.

#### Risk of Bias

Stringent selection criteria substantially reduced the risk of bias. For the meta-analysis, we included only studies that reported on a clinical cohort of patients with OCD, who were not selected (included or excluded) on the basis of any comorbidities. A diagnosis of OCD and comorbid disorders was made using standardised assessment instruments. In our estimation of pooled prevalence rates, we have considered several covariates by moderator analyses, as discussed earlier. Sensitivity analyses were conducted with Baujat plots, influence plots and the leave-one-out method. A considerable impact on the pooled prevalence rate was found only for OCPD. The outlier study, in this case, was removed in the final pooled prevalence estimation. For all other comorbidities, outliers did not significantly change the pooled prevalence estimates.

#### Inconsistency

Heterogeneity was considerable, and statistically significant, across comorbidities except for ASD, Bulimia nervosa and ASPD. As discussed, this may be due to variations in demographic, socio-cultural, clinical characteristics and study methods. With sub-group analysis for adult, vs. paediatric, studies, we have accounted for some of these variables. Our selection criteria required a minimum sample size of 100 OCD patients. Our interest was to detect comorbidities at a low rate of 1%. The included studies however varied in the sample sizes. Only one study had a sample size of 100. A majority (49.5%) of the studies had a sample size between 101 and 200, another 23.1% had a sample size of 201–300. Inconsistency may have impacted the results of the meta-analysis.

#### Imprecision

This is possibly a problem with select comorbidities. The pooled prevalence rates (across adult and paediatric studies) have wide confidence intervals for OCRDs, varying from <5 to >25% for any OCRD; <1–>6% for BDD, and ~1–>7% for Trichotillomania. If we consider only studies on adults, the confidence intervals widen, for any OCRD varying between ~6 and >30%; for BDD <1–>7%; for trichotillomania between ~1 and >10%. Wide confidence intervals were also seen for three comorbidities in the paediatric sub-group—MDD [17.1% (95% CI 6.3–31.6)], Panic disorder [6.1% (95% CI 0.5–16.4)], and ODD [12.5% (95% CI 2.3–28.7)]. Therefore, the pooled prevalence rates for these comorbid disorders score low in terms of precision.

#### Indirectness

The meta-analysis in this paper is generalizable to clinical populations of patients with OCD. We have attempted to compare pooled prevalence rates with those seen in community based studies, with comparable sample sizes of OCD patients, however, such studies were only 6 in number. A large number of community based studies were excluded because, expectedly, they had much smaller numbers of OCD patients.

#### Publication Bias

Funnel plot, Egger's test, and Begg's test were used to examine publication bias. Either of the two tests was significant for schizophrenia/any psychotic disorder, OCPD and narcissistic personality disorder. Funnel plots showed asymmetry for dysthymia, agoraphobia, PTSD, any eating disorder, and schizoid personality disorder. The pooled prevalence rates for these comorbidities may therefore have been affected by publication bias. Nearly 50% of the 91 included studies in the meta-analysis reported rates for MDD, however, personality disorders, and psychotic disorders were reported in <20% of the studies, suggesting that these comorbidities are left out in the evaluation of comorbidities in clinical studies, perhaps due to their comparatively lower prevalence.

Three comorbidities (ASD, Bulimia nervosa and ASPD) had a GRADE score 5 out of 5; most other comorbidities (*n* = 21) scored a 4 out of 5, and the remaining (*n* = 12) scored a 3 out of 5. MDD and panic disorder also scored a 3 out of 5 for paediatric studies. Based on ratings on the GRADE criteria, we interpret a moderate to high confidence in the pooled prevalence rates for comorbidities in OCD from our meta-analysis.

## Discussion

This is the first systematic review and meta-analysis to examine comorbid disorders across the lifespan in individuals with OCD. The meta-analysed studies were clinic-based and reported original findings on individuals with OCD, evaluated using standardised diagnostic interviews/instruments. We chose to report lifetime comorbidities in this paper, since these were reported in the largest number of studies. We grouped the studies into adult and paediatric subgroups to examine comorbidity rates from a lifespan perspective. Meta-analyses for individual comorbidities had high heterogeneity (*I*^2^ 80–98). High heterogeneity may reflect variations in demographic (mean age, gender distribution), socio-cultural (country of origin), and clinical (age of onset of OCD, illness severity) characteristics, besides differences in research methods (study design, recruitment source, decision-making processes, measurement errors, recall biases in reporting lifetime comorbidity etc.). Interestingly, heterogeneity was statistically non-significant for ASD, bulimia nervosa and anti-social personality disorder (ASPD). Based on the GRADE criteria (risk of bias, inconsistency, imprecision and indirectness, and publication bias) we report a moderate to high confidence in the presented pooled estimates.

### Comorbid Disorders in OCD

Lifetime psychiatric comorbidities were present in 69% of the pooled sample. All comorbidities were manifold times higher than general population prevalence expected for the individual disorders (49–55, 57, 59–64, 74, 75). Overall rates were slightly (although non-significantly) higher in adult (71%) vs. paediatric (64%) subgroups, which stands to reason given that lifetime rates were being noted, and as paediatric OCD is known to persist long-term in ~40% of cases (76). Mood disorders, anxiety disorders, NDDs and OCRDs were the most common comorbidities. Bio-psycho-social commonalities across these disorders spanning genetic, temperamental (32, 33), and neuropsychological vulnerabilities (77) plausibly account for these observations. Community based surveys on adults were similar to clinic-based studies in terms of the most common comorbid disorders (depressive disorders followed by anxiety disorders), however, at much lower prevalence rates.

### Comorbidity Patterns in Adult and Paediatric Subgroups

Commonest lifetime comorbidities differed across adult and paediatric subgroups. Anxiety disorders were the most common in children, vs. mood disorders in adults. These perhaps reflect etiopathological origins and natural history of these disorders. Anxious temperament traits such as high harm avoidance (78) and shyness/behavioural inhibition (79) are seen in children with anxiety disorders and OCD. As such, a shared developmental/temperamental vulnerability may lead to emergence of both disorder types at a young age (80, 81). Compulsive behaviours possibly provide anxious youth with increased perceived control over uncertainty, given that they have less direct control over their environment than adults (80). A shared vulnerability to anxiety could also explain our findings of an association between lower AOO of OCD and higher comorbid rates of GAD. In comparison, onset of mood disorders (regardless of OCD comorbidity) tends to occur after that of anxiety disorders, and often in adulthood (82). The high lifetime prevalence of mood disorders in adults could suggest a secondary impact of OCD over time, i.e., resultant from cumulative patterns of avoidance, increased negative emotional states, impairment, and reduced quality of life.

The similarity between NDD comorbidity rates across adult and paediatric subgroups is potentially unexpected, and subsequently worthy of discussion. It is generally believed that NDD comorbidities are more prevalent in children than adults with OCD. This would be reflected in rates of current comorbidity, as opposed to our examination of lifetime comorbidity in this meta-analysis. The youngest sample (83) in this meta-analysis had a mean age of around 12 years, therefore the pooled sample had crossed the typical age of onset/presentation for ADHD and tic disorders (84). Similar lifetime rates for NDDs in children and adults with OCD suggest a neurodevelopmental continuum in the etiopathogenesis of this disorder.

Comorbidity rates of ASD could be reliably pooled only from paediatric studies. ASD and OCD are often difficult to differentiate in young children due to an inadequately developed verbal repertoire to express experiences of obsessions, and the phenomenologically similar repetitiveness and inflexibility in behaviours (85). According to our analysis, around 6% children with OCD may have comorbid ASD. The reverse comorbidity, i.e., OCD in children with ASD, is almost double (86).

Childhood OCD affects not only the individual child, but invariably also involves primary caregivers, by means of family accommodation (87). Moreover, therapists and parents often find it challenging to differentiate avoidance, and anxiety-related impairment from, say, oppositional behaviours. In this context, a comorbid ODD rate of 12% indicates the need for comprehensive functional behavioural analysis, as well as a careful psychotherapeutic plan that ties in treatment components for both disorders (88).

### Age Trends in Comorbidity

To better understand comorbidities across the lifespan, we used two age related variables, AAA and AOO, in moderator analysis by meta-regression. An increasing AAA was associated with an increase in prevalence of MDD, panic disorder and SUDs, and a decrease in prevalence of GAD and psychosis. An increase in AOO was associated with an increase in prevalence of personality disorders and a decrease in prevalence of GAD, PTSD, BDD, ODD, and agoraphobia. These trends are clinically informative in guiding inquiry for comorbidities.

The typical onset for MDD (82), panic disorder (89), and SUDs (90) is around early adulthood. We found the same when they were comorbid with OCD. It is possible that the detrimental psychological impact of a chronic and disabling OCD manifests around the stressful period of transition to adulthood in the form of depressive and panic disorders or as maladaptive coping with substance use. Personality disorders may reflect another vulnerability profile wherein OCD is triggered during a stressful transition to adulthood. In contrast, anxiety related comorbidities (GAD, PTSD, agoraphobia) decrease with a later AOO. As discussed earlier, these seem to emerge from shared developmental/temperamental vulnerabilities between OCD and anxiety disorders in youth. The interactive influences of childhood traumatic experiences, dissociative experiences, and vulnerabilities to anxiety are interesting in this regard (91, 92). Consistent with their emergence in young childhood and adolescence, comorbidity rates for ODD and BDD also fell with later AOO.

The above discussion suggests that clinicians should be aware of NDD comorbidities throughout the life span, while also considering higher risk of disruptive behaviours in young children, OCRDs (BDD), PTSD, and anxiety disorders (GAD) in children and adolescents, and the emergence of mood/anxiety, substance use, and personality disorders during the transition to adulthood.

### Gender Differences in Comorbidity

Consistent with previous research indicating greater comorbidity risk in males compared to females (24, 93), samples with a higher proportion of males were associated with higher comorbidity rates of any psychiatric disorder, ADHD, bipolar disorder, psychosis, specific phobia, agoraphobia, and ODD. Surprisingly, we did not find a higher prevalence of tic disorders and substance use disorders among males. The lack of a gender association in mood and anxiety disorder prevalence was also consistent with previous work (24, 93, 94); however, unlike previous reports, we did not find a higher prevalence of eating disorders in females (95).

### Illness Severity may Preclude a Diagnosis of Certain Comorbidities

We found that a high illness severity was associated with low comorbidity rates for tic disorders, panic disorder, OCRDs, anorexia nervosa and OCPD. Our finding on tic disorders is in line with previous research in that tic-related OCD severity was either comparable (11, 96) or measured as being lower (97) than non-tic related OCD in baseline measures. In fact, a longitudinal study recorded higher rates and a shorter time to remission in youth with OCD, suggesting that developmental processes that result in a natural remission of tics possibly impact comorbid OCD outcomes as well (98).

There may also be a practical clinical explanation for these findings. As these comorbidities have phenomenological overlap with OCD—repetitive thoughts/urges/behaviours in tics, OCRDs, anorexia nervosa and OCPD; distressing anxiety in panic disorder—clinicians may be more likely to subsume comorbid symptoms under the OCD diagnosis in those with a severe illness. For example, if an individual's panic attacks are understood as resulting from the excessive anxiety triggered by obsessions, they might not be identified as a distinct comorbidity. Similarly, certain motor or vocal tics may be mistaken for compulsive behaviours triggered by sensory phenomena, while cognitions driving OCRDs, anorexia nervosa and OCPD may be identified as obsessive in nature. On the other hand, clinicians who conceptualise these disorders as distinct comorbid phenomena may rate a lower severity for OCD when contributions from comorbidities are disregarded. It is implied that diagnostic evaluation be done over multiple consultations and repeated in follow-up for clarity on phenomenological and comorbidity profile.

### Comorbid Personality Disorders in OCD

Personality disorders were prevalent in ~35% of the pooled sample from studies on adults with OCD. While this rate was lower than mood disorders (54%), it was similar to anxiety disorders (32%). OCPD was the most common personality disorder (17%), followed by anxious-avoidant personality disorder (AAPD) (9%), and borderline personality disorder (BPD) (9%). Given these high comorbidity rates, it is noteworthy that <15% studies on adult OCD reported personality disorders. An earlier meta-analysis reported OCD to have a higher probability of comorbid personality disorders, in comparison to anxiety disorders (99). A report from the Nepean OCD study suggested that comorbid OCPD is associated with *prominent* symptoms, *intense* co-occurring psychopathology, and greater distress (100). In another systematic examination of clinical correlates of comorbid personality disorders in OCD, phenomenological and mood disorder comorbidity related differences were reported across OCPD, BPD, and AAPD (101).

### Implications

This is, to our knowledge, the largest meta-analysis of OCD comorbidity over the lifespan. Lifetime comorbid psychiatric illness is the rule rather than the exception in OCD, regardless of age. This may reflect common underpinnings across psychiatric disorders (102), overlapping phenotypes and/or sequelae of OCD pathology. In this context, information from our meta-analysis on base rates of common comorbid disorders with OCD would improve their identification and overall diagnostic efficiency (103). Our findings suggest that age, both of onset and at assessment, is a relevant factor influencing comorbidity profile and thereby, also treatment. Clinicians must screen for neurodevelopmental disorders in both children and adults, for anxiety and obsessive-compulsive related disorders in children, and for mood, substance-use, and personality disorders in adults. A high prevalence of comorbid personality disorders emphasises the need to include these in the clinical evaluation of all adults presenting with OCD. Males have a higher risk for comorbidity with NDDs, SMIs, and certain anxiety disorders. Several phenomenologically similar comorbidities such as tic disorders, OCRDs, OCPD, anorexia nervosa, and panic disorder, may be missed in individuals with a severe OCD. This suggests a need to track not only diagnostic severity over time, but also symptom profiles, for a more definitive comorbidity assessment.

High heterogeneity values in our analyses, and consequent wide prediction intervals, result from a high inter-study variability. These persist even with sub-group analyses for paediatric vs. adult studies. Quality-controlled, multi-centre, large studies, using prospective designs are needed to evaluate comorbidities in OCD comprehensively.

### Limitations

The following limitations in our research are noted. First, paediatric samples were under-represented compared to adults, likely due to general limitations in the extant literature on childhood OCD and our selection criteria requiring a OCD sample size of ≥100. Second, comorbidity evaluation was not consistent across studies, with several comorbidities not commonly reported in paediatric OCD studies (e.g., bipolar disorder, OCRDs, eating disorders, PTSD, psychosis) and only one adult study recording comorbidity rates for ASD. Third, a more nuanced lifespan examination of comorbidities was not possible given the nature of included samples and a lack of studies representing the elderly population with OCD. Fourth, several studies did not explicitly state assessment of lifetime vs. current comorbidities. To maximise data utilisation from the studies we treated all studies unclear in this regard as reporting lifetime comorbidity rates. Fifth, we did not find enough no. of studies that reported medical/neurological comorbidities. Sixth, due to lack of clarity on their use of standardised assessments, several large samples (e.g., registry based datasets), were not represented. Seventh, we could include only clinic-based studies for meta-analysis, given the conceptual differences from community-based studies of which there were only a few with substantial numbers of individuals with OCD. The results of the meta-analysis are generalizable, therefore, to treatment-seeking clinical populations. However, clinic-based studies may suffer from Berkson's bias (104), i.e., clinical samples of OCD are more likely to be comorbid and therefore may show higher comorbidities compared to general population or community settings. Eighth, given the aims and nature of epidemiologic research, our inclusion Criteria for a sample size of ≥100 individuals with OCD would have resulted in the exclusion of a large number of community-based studies, especially those that assessed children and adolescents. Finally, while outside of the scope of the current paper, it is notable that several other factors may influence comorbidity rates, including study design, year of publication, country of origin, and socio-cultural differences.

### Future Directions

It would be useful to study comorbidity patterns uniformly, using standardised diagnostic instruments, across multiple centres. Prospective follow up of large clinical and community cohorts of OCD, would enable one to track development, onset and evolution of comorbidity, and identify putative risk factors that may inform better interventions, including prevention strategies. Such systematic data may also inform further study into phenomenological/ clinical endophenotypes of obsessive-compulsive disorder.

## Conclusion

In this first meta-analysis on comorbidities in OCD across the lifespan we found that more than two thirds of patients, children or adults, have comorbid disorders. NDDs are equally prevalent in children and adults, whereas anxiety disorders (children > adults) and mood disorders (adults > children) show age related variations. Age at assessment, gender, age of onset, and illness severity are significant factors impacting comorbidity prevalence in OCD. This meta-analysis suggests the need for a screening, guided by the age at assessment, and a longitudinal tracking, especially of symptoms that may be phenomenologically related to OCD, for a comprehensive ascertainment of comorbidities.

## Data Availability Statement

The original contributions presented in the study are included in the article/Supplementary Material, further inquiries can be directed to the corresponding author/s.

## Author Contributions

ES coordinated the review. ES, LPS, SB, BL, HM, PK, CL, KG, TLT, and ACLA screened articles for selection and extracted data from finally included studies. SB conducted the statistical analysis. ES, SB, and LPS prepared the first draught of the paper and worked on revisions. RRS worked on revisions in the manuscript. RRS, GS, DRMAH, and SES supervised work at every step of this paper, besides resolving conflicts during the study screening process, and contributing to revisions of the manuscript. All authors contributed to the article and approved the submitted version.

## Conflict of Interest

The authors declare that the research was conducted in the absence of any commercial or financial relationships that could be construed as a potential conflict of interest.

## Publisher's Note

All claims expressed in this article are solely those of the authors and do not necessarily represent those of their affiliated organizations, or those of the publisher, the editors and the reviewers. Any product that may be evaluated in this article, or claim that may be made by its manufacturer, is not guaranteed or endorsed by the publisher.

## References

[1] American Psychiatric Association. Diagnostic and Statistical Manual of Mental Disorders: DSM-5. 5th ed. Washington, DC: American Psychiatric Association (2013). 10.1176/appi.books.9780890425596

[2] Pittenger C. Obsessive-Compulsive DisorderPhenomenology, Pathophysiology, and Treatment: Phenomenology, Pathophysiology, and Treatment. Oxford: Oxford University Press (2017). 10.1093/med/9780190228163.001.0001

[3] Lewin AB Park JM Jones AM Crawford EA DeNadai AS Menzel J . Family-based exposure and response prevention therapy for preschool-aged children with obsessive-compulsive disorder: a pilot randomized controlled trial. Behav Res Ther. (2014) 56:30–8. 10.1016/j.brat.2014.02.001 24657310

[4] Sharma E Jacob P Dharmendra A Reddy YC J Seshadri SP Girimaji SC . Preschool onset OCD: a review of literature and clinical experience. Bull Menninger Clin. (2021) 85:298–315. 10.1521/bumc.2021.85.3.298 34468213

[5] Weiss AP Jenike MA. Late-onset obsessive-compulsive disorder: a case series. J Neuropsychiatry Clin Neurosci. (2000) 12:265–8. 10.1176/jnp.12.2.265 11001607

[6] Albert U Manchia M Tortorella A Volpe U Rosso G Carpiniello B . Admixture analysis of age at symptom onset and age at disorder onset in a large sample of patients with obsessive-compulsive disorder. J Affect Disord. (2015) 187:188–96. 10.1016/j.jad.2015.07.045 26339929

[7] Taylor S. Early versus late onset obsessive-compulsive disorder: evidence for distinct subtypes. Clin Psychol Rev. (2011) 31:1083–100. 10.1016/j.cpr.2011.06.007 21820387

[8] Ivarsson T Melin K Wallin L. Categorical and dimensional aspects of co-morbidity in obsessive-compulsive disorder (OCD). Eur Child Adolesc Psychiatry. (2008) 17:20–31. 10.1007/s00787-007-0626-z 18004647

[9] Attademo L Bernardini F. Schizotypal personality disorder in clinical obsessive-compulsive disorder samples: a brief overview. CNS Spectr. (2020) 26:468–80. 10.1017/S1092852920001716 32713392

[10] Conelea CA Walther MR Freeman JB Garcia AM Sapyta J Khanna M . Tic-related obsessive-compulsive disorder (OCD): phenomenology and treatment outcome in the pediatric OCD treatment study II. J Am Acad Child Adolesc Psychiatry. (2014) 53:1308–16. 10.1016/j.jaac.2014.09.014 25457929 PMC4254546

[11] Højgaard DRMA Skarphedinsson G Nissen JB Hybel KA Ivarsson T Thomsen PH. Pediatric obsessive–compulsive disorder with tic symptoms: clinical presentation and treatment outcome. Eur Child Adolesc Psychiatry. (2017) 26:681–9. 10.1007/s00787-016-0936-0 28032202

[12] Jaisoorya TS Reddy YCJ Srinath S Thennarasu K. Obsessive-compulsive disorder with and without tic disorder: a comparative study from India. CNS Spectr. (2008) 13:705–11. 10.1017/S1092852900013791 18704026

[13] Pozza A Starcevic V Ferretti F Pedani C Crispino R Governi G . Obsessive-compulsive personality disorder co-occurring in individuals with obsessive-compulsive disorder: a systematic review and meta-analysis. Harv Rev Psychiatry. (2021) 29:95–107. 10.1097/HRP.0000000000000287 33666394

[14] Albert U Aguglia A Chiarle A Bogetto F Maina G. Metabolic syndrome and obsessive-compulsive disorder: a naturalistic Italian study. Gen Hosp Psychiatry. (2013) 35:154–9. 10.1016/j.genhosppsych.2012.10.004 23158675

[15] Klein KP Harris EK Björgvinsson T Kertz SJ. A network analysis of symptoms of obsessive compulsive disorder and depression in a clinical sample. J Obsessive-Compuls Relat Disord. (2020) 27:100556. 10.1016/j.jocrd.2020.100556

[16] Mancebo MC Grant JE Pinto A Eisen JL Rasmussen SA. Substance use disorders in an obsessive compulsive disorder clinical sample. J Anxiety Disord. (2009) 23:429–35. 10.1016/j.janxdis.2008.08.008 18954963 PMC2705178

[17] Deacon BJ Abramowitz JS. The yale-brown obsessive compulsive scale: factor analysis, construct validity, and suggestions for refinement. J Anxiety Disord. (2005) 19:573–85. 10.1016/j.janxdis.2004.04.009 15749574

[18] Leonard RC Jacobi DM Riemann BC Lake PM Luhn R. The effect of depression symptom severity on OCD treatment outcome in an adolescent residential sample. J Obsessive-Compuls Relat Disord. (2014) 3:95–101. 10.1016/j.jocrd.2014.02.003

[19] Leckman JF Grice DE Barr LC de Vries AL Martin C Cohen DJ . Tic-related vs. non-tic-related obsessive compulsive disorder. Anxiety. (1994) 1:208–15. 9160576

[20] Pardue CM Sibrava NJ Boisseau CL Mancebo MC Eisen JL Rasmussen SA. Differential parental influence in the familial aggregation of obsessive compulsive disorder. J Obsessive Compuls Relat Disord. (2014) 3:215–9. 10.1016/j.jocrd.2014.05.001 25068099 PMC4110208

[21] Geller D Biederman J Faraone SV Frazier J Coffey BJ Kim G . Clinical correlates of obsessive compulsive disorder in children and adolescents referred to specialized and non-specialized clinical settings. Depress Anxiety. (2000) 11:163–8. 10.1002/1520-6394(2000)11:4<163::AID-DA3>3.0.CO;2-3 10945136

[22] Goodman WK Price LH Rasmussen SA Mazure C Fleischmann RL Hill CL . The yale-brown obsessive compulsive scale. I. Development, use, and reliability. Arch Gen Psychiatry. (1989) 46:1006–11. 10.1001/archpsyc.1989.01810110048007 2684084

[23] Goodman WK Price LH Rasmussen SA Mazure C Delgado P Heninger GR . The yale-brown obsessive compulsive scale. II. validity. Arch Gen Psychiatry. (1989) 46:1012–6. 10.1001/archpsyc.1989.01810110054008 2510699

[24] de Mathis MA de Alvarenga P Funaro G Torresan RC Moraes I Torres AR . Gender differences in obsessive-compulsive disorder: a literature review. Rev Bras Psiquiatr. (2011) 33:390–9. 10.1590/S1516-44462011000400014 22189930

[25] Kalra SK Swedo SE. Children with obsessive-compulsive disorder: are they just “little adults”? J Clin Invest. (2009) 119:737–46. 10.1172/JCI37563 19339765 PMC2662563

[26] Mathes BM Morabito DM Schmidt NB. Epidemiological and clinical gender differences in OCD. Curr Psychiatry Rep. (2019) 21:36. 10.1007/s11920-019-1015-2 31016410

[27] Sharma E Sundar AS Thennarasu K Reddy YCJ. Is late-onset OCD a distinct phenotype? Findings from a comparative analysis of “age at onset” groups. CNS Spectr. (2015) 20:508–14. 10.1017/S1092852914000777 26189938

[28] Abramowitz JS Jacoby RJ. Obsessive-compulsive and related disorders: a critical review of the new diagnostic class. Annu Rev Clin Psychol. (2015) 11:165–86. 10.1146/annurev-clinpsy-032813-153713 25581239

[29] Selles RR Storch EA Lewin AB. Variations in symptom prevalence and clinical correlates in younger versus older youth with obsessive-compulsive disorder. Child Psychiatry Hum Dev. (2014) 45:666–74. 10.1007/s10578-014-0435-9 24549726

[30] Overbeek T Schruers K Vermetten E Griez E. Comorbidity of obsessive-compulsive disorder and depression: prevalence, symptom severity, and treatment effect. J Clin Psychiatry. (2002) 63:1106–12. 10.4088/JCP.v63n1204 12523869

[31] Storch EA Lewin AB Larson MJ Geffken GR Murphy TK Geller DA. Depression in youth with obsessive-compulsive disorder: clinical phenomenology and correlates. Psychiatry Res. (2012) 196:83–9. 10.1016/j.psychres.2011.10.013 22370151

[32] Radonjić NV Hess JL Rovira P Andreassen O Buitelaar JK Ching CRK . Structural brain imaging studies offer clues about the effects of the shared genetic etiology among neuropsychiatric disorders. Mol Psychiatry. (2021) 26:2101–10. 10.1038/s41380-020-01002-z 33456050 PMC8440178

[33] Yang Z Wu H Lee PH Tsetsos F Davis LK Yu D . Investigating shared genetic basis across tourette syndrome and comorbid neurodevelopmental disorders along the impulsivity-compulsivity spectrum. Biol Psychiatry. (2021) 90:317–27. 10.1016/j.biopsych.2020.12.028 33714545 PMC9152955

[34] Hofmeijer-Sevink MK Van Oppen P Van Megen HJ Batelaan NM Cath DC Van Der Wee NJA . Clinical relevance of comorbidity in obsessive compulsive disorder: the Netherlands OCD association study. J Affect Disord. (2013) 150:847–54. 10.1016/j.jad.2013.03.014 23597943

[35] Moher D Liberati A Tetzlaff J Altman DG The PRISMA group. Preferred reporting items for systematic reviews and meta-analyses: the PRISMA statement. PLoS Med. (2009) 6:e1000097. 10.1371/journal.pmed.1000097 19621072 PMC2707599

[36] Medicode (Firm). ICD-9-CM: International Classification of Diseases, 9th Revision, Clinical Modification. 9th ed. Salt Lake City, UT: Medicode (1996).

[37] Volkmar FR. DSM-III. In: Volkmar FR, editor. Encyclopedia of Autism Spectrum Disorders. New York, NY: Springer New York (2013). p. 999–1001.

[38] Scahill L Riddle MA McSwiggin-Hardin M Ort SI King RA Goodman WK . Children's yale-brown obsessive compulsive scale: reliability and validity. Fifth Rev. (1997) 36:844–852. 10.1097/00004583-199706000-00023 9183141

[39] R Core Team. R: A Language and Environment for Statistical Computing. Vienna: R Foundation for Statistical Computing (2020). Available online at: https://www.R-project.org/

[40] Viechtbauer W. Conducting meta-analyses in R with the metafor package. J Stat Softw. (2010) 36:1–48. 10.18637/jss.v036.i03

[41] Balduzzi S Rücker G Schwarzer G. How to perform a meta-analysis with R: a practical tutorial. Evid Based Ment Health. (2019) 22:153–60. 10.1136/ebmental-2019-300117 31563865 PMC10231495

[42] Coburn KM Vevea JL. Package “weightr”: Estimating Weight-Function Models for Publication Bias in R. (2019). Available online at: https://cran.r-project.org/web/packages/weightr/index.html (accessed March 31, 2021).

[43] Deeks JJ Higgins JP Altman DG on behalf of the Cochrane Statistical Methods Group. Analysing data undertaking meta-analyses. In: Higgins JPT Thomas J Chandler J Cumpston M Li T Page MJ Welch VA, editors. Cochrane Handbook for Systematic Reviews of Interventions (Chichester, UK: Wiley). p. 241–84. 10.1002/9781119536604.ch10

[44] Boileau B. A review of obsessive-compulsive disorder in children and adolescents. Dialogues Clin Neurosci. (2011) 13:401–11. 10.31887/DCNS.2011.13.4/bboileau 22275846 PMC3263388

[45] Torres AR Fontenelle LF Shavitt RG Hoexter MQ Pittenger C Miguel EC. Epidemiology, Comorbidity, and Burden of OCD. Pittenger C, editor. Oxford, UK: Oxford University Press (2017). 10.1093/med/9780190228163.003.0004

[46] American Psychiatric Association. Diagnostic and Statistical Manual of Mental Disorders: DSM-IV. Washington, DC: American Psychiatric Association (1994).

[47] World Health Organization. The ICD-10 Classification of Mental and Behavioural Disorders: Clinical Descriptions and Diagnostic Guidelines. Geneva: World Health Organization (1992).

[48] United Nations Development Programme. 2020 Human Development Index Ranking. (2020). Available online at: http://hdr.undp.org/en/content/latest-human-development-index-ranking (accessed April 17, 2021).

[49] Vos T Lim SS Abbafati C Abbas KM Abbasi M Abbasifard M . Global burden of 369 diseases and injuries in 204 countries and territories, 1990–2019: a systematic analysis for the global burden of disease study 2019. Lancet. (2020) 396:1204–22. 10.1016/S0140-6736(20)30925-9 33069326 PMC7567026

[50] Polanczyk GV Salum GA Sugaya LS Caye A Rohde LA. Annual research review: a meta-analysis of the worldwide prevalence of mental disorders in children and adolescents. J Child Psychol Psychiatry. (2015) 56:345–65. 10.1111/jcpp.12381 25649325

[51] Steel Z Marnane C Iranpour C Chey T Jackson JW Patel V . The global prevalence of common mental disorders: a systematic review and meta-analysis 1980–2013. Int J Epidemiol. (2014) 43:476–93. 10.1093/ije/dyu038 24648481 PMC3997379

[52] Meter ARV Moreira ALR Youngstrom EA. Meta-Analysis of epidemiologic studies of pediatric bipolar disorder. J Clin Psychiatry. (2011) 72:1250–6. 10.4088/JCP.10m06290 21672501

[53] Ruscio AM Hallion LS Lim CCW Aguilar-Gaxiola S Al-Hamzawi A Alonso J . Cross-sectional comparison of the epidemiology of DSM-5 generalized anxiety disorder across the globe. JAMA Psychiatry. (2017) 74:465–75. 10.1001/jamapsychiatry.2017.0056 28297020 PMC5594751

[54] Stein DJ Lim CCW Roest AM de Jonge P Aguilar-Gaxiola S Al-Hamzawi A . The cross-national epidemiology of social anxiety disorder: data from the world mental health survey initiative. BMC Med. (2017) 15:143. 10.1186/s12916-017-0889-2 28756776 PMC5535284

[55] Jonge P de Roest AM Lim CCW Florescu SE Bromet EJ Stein DJ . Cross-national epidemiology of panic disorder and panic attacks in the world mental health surveys. Depress Anxiety. (2016) 33:1155–77. 10.1002/da.22572 27775828 PMC5143159

[56] Roest AM Vries YA de Lim CCW Wittchen H-U Stein DJ Adamowski T . A comparison of DSM-5 and DSM-IV agoraphobia in the world mental health surveys. Depress Anxiety. (2019) 36:499–510. 10.1002/da.22885 30726581 PMC6548607

[57] Wardenaar KJ Lim CCW Al-Hamzawi AO Alonso J Andrade LH Benjet C . The cross-national epidemiology of specific phobia in the world mental health surveys. Psychol Med. (2017) 47:1744–60. 10.1017/S0033291717000174 28222820 PMC5674525

[58] Simon V Czobor P Bálint S Mészáros Á Bitter I. Prevalence and correlates of adult attention-deficit hyperactivity disorder: meta-analysis. Br J Psychiatry. (2009) 194:204–11. 10.1192/bjp.bp.107.048827 19252145

[59] Knight T Steeves T Day L Lowerison M Jette N Pringsheim T. Prevalence of tic disorders: a systematic review and meta-analysis. Pediatr Neurol. (2012) 47:77–90. 10.1016/j.pediatrneurol.2012.05.002 22759682

[60] Elsabbagh M Divan G Koh Y-J Kim YS Kauchali S Marcín C . Global prevalence of autism and other pervasive developmental disorders. Autism Res. (2012) 5:160–79. 10.1002/aur.239 22495912 PMC3763210

[61] Veale D Gledhill LJ Christodoulou P Hodsoll J. Body dysmorphic disorder in different settings: a systematic review and estimated weighted prevalence. Body Image. (2016) 18:168–86. 10.1016/j.bodyim.2016.07.003 27498379

[62] Qian J Hu Q Wan Y Li T Wu M Ren Z . Prevalence of eating disorders in the general population: a systematic review. Shanghai Arch Psychiatry. (2013) 25:212–23. 10.3969/j.issn.1002-0829.2013.04.003 24991159 PMC4054558

[63] Koenen KC Ratanatharathorn A Ng L McLaughlin KA Bromet EJ Stein DJ . Posttraumatic stress disorder in the world mental health surveys. Psychol Med. (2017) 47:2260–74. 10.1017/S0033291717000708 28385165 PMC6034513

[64] Moreno-Küstner B Martín C Pastor L. Prevalence of psychotic disorders and its association with methodological issues. A systematic review and meta-analyses. PLoS ONE. (2018) 13:e0195687. 10.1371/journal.pone.0195687 29649252 PMC5896987

[65] Karno M Golding JM Sorenson SB Burnam A. The epidemiology of obsessive-compulsive disorder in five US communities. Arch Gen Psychiatry. (1988) 45:1094–9. 10.1001/archpsyc.1988.01800360042006 3264144

[66] Torres AR Prince MJ Bebbington PE Bhugra D Brugha TS Farrell M . Obsessive-compulsive disorder: prevalence, comorbidity, impact, and help-seeking in the British national psychiatric morbidity survey of 2000. Am J Psychiatry. (2006) 163:1978–85. 10.1176/ajp.2006.163.11.1978 17074950

[67] Torres AR Moran P Bebbington P Brugha T Bhugra D Coid JW . Obsessive-compulsive disorder and personality disorder. Soc Psychiatry Psychiatr Epidemiol. (2006) 41:862–7. 10.1007/s00127-006-0118-3 16983489

[68] Mohammadi M-R Ghanizadeh A Moini R. Lifetime comorbidity of obsessive-compulsive disorder with psychiatric disorders in a community sample. Depress Anxiety. (2007) 24:602–7. 10.1002/da.20259 17152061

[69] Subramaniam M Abdin E Vaingankar JA Chong SA. Obsessive–compulsive disorder: prevalence, correlates, help-seeking and quality of life in a multiracial Asian population. Soc Psychiatry Psychiatr Epidemiol. (2012) 47:2035–43. 10.1007/s00127-012-0507-8 22526825

[70] Subramaniam M Abdin E Vaingankar J Shafie S Chang S Seow E . Obsessive-compulsive disorder in Singapore: prevalence, comorbidity, quality of life and social support. Ann Acad Med Singapore. (2020) 49:15–25. 10.47102/annals-acadmedsg.2019185 32200393

[71] Nelson E Rice J. Stability of diagnosis of obsessive-compulsive disorder in the epidemiologic catchment area study. Am J Psychiatry. (1997) 154:826–31. 10.1176/ajp.154.6.826 9167511

[72] Borges Migliavaca C Stein C Colpani V Barker TH Munn Z Falavigna M . How are systematic reviews of prevalence conducted? A methodological study. BMC Med Res Methodol. (2020) 20:96. 10.1186/s12874-020-00975-3 32336279 PMC7184711

[73] Iorio A Spencer FA Falavigna M Alba C Lang E Burnand B . Use of GRADE for assessment of evidence about prognosis: rating confidence in estimates of event rates in broad categories of patients. BMJ. (2015) 350:h870. 10.1136/bmj.h870 25775931

[74] Simon W. Follow-up psychotherapy outcome of patients with dependent, avoidant and obsessive-compulsive personality disorders: a meta-analytic review. Int J Psychiatry Clin Pract. (2009) 13:153–65. 10.1080/13651500802570972 24916735

[75] Winsper C Bilgin A Thompson A Marwaha S Chanen AM Singh SP . The prevalence of personality disorders in the community: a global systematic review and meta-analysis. Br J Psychiatry. (2020) 216:69–78. 10.1192/bjp.2019.166 31298170

[76] Stewart SE Geller DA Jenike M Pauls D Shaw D Mullin B . Long-term outcome of pediatric obsessive-compulsive disorder: a meta-analysis and qualitative review of the literature. Acta Psychiatr Scand. (2004) 110:4–13. 10.1111/j.1600-0447.2004.00302.x 15180774

[77] Brem S Grünblatt E Drechsler R Riederer P Walitza S. The neurobiological link between OCD and ADHD. ADHD Atten Deficit Hyperact Disord. (2014) 6:175–202. 10.1007/s12402-014-0146-x 25017045 PMC4148591

[78] Marchesi C Ampollini P DePanfilis C Maggini C. Temperament features in adolescents with ego-syntonic or ego-dystonic obsessive-compulsive symptoms. Eur Child Adolesc Psychiatry. (2008) 17:392–6. 10.1007/s00787-008-0681-0 18427866

[79] Ivarsson T Winge-Westholm C. Temperamental factors in children and adolescents with obsessive-compulsive disorder (OCD) and in normal controls. Eur Child Adolesc Psychiatry. (2004) 13:365–72. 10.1007/s00787-004-0411-1 15619049

[80] Comer JS Kendall PC Franklin ME Hudson JL Pimentel SS. Obsessing/worrying about the overlap between obsessive-compulsive disorder and generalized anxiety disorder in youth. Clin Psychol Rev. (2004) 24:663–83. 10.1016/j.cpr.2004.04.004 15385093

[81] Gillett CB Bilek EL Hanna GL Fitzgerald KD. Intolerance of uncertainty in youth with obsessive-compulsive disorder and generalized anxiety disorder: a transdiagnostic construct with implications for phenomenology and treatment. Clin Psychol Rev. (2018) 60:100–8. 10.1016/j.cpr.2018.01.007 29426573

[82] Yalin N Young AH. The age of onset of unipolar depression. In: de Girolamo G McGorry PD Sartorius N, editor. Age of Onset of Mental Disorders. Cham: Springer International Publishing (2019). p. 111–24. 10.1007/978-3-319-72619-9_6

[83] Piacentini J Bergman RL Keller M McCracken J. Functional impairment in children and adolescents with obsessive-compulsive disorder. J Child Adolesc Psychopharmacol. (2003) 13:S61–9. 10.1089/104454603322126359 12880501

[84] Bagheri MM Kerbeshian J Burd L. Recognition and management of tourette's syndrome and tic disorders. Am Fam Phys. (1999) 59:2263–2272. 10221310

[85] Postorino V Kerns CM Vivanti G Bradshaw J Siracusano M Mazzone L. Anxiety disorders and obsessive-compulsive disorder in individuals with autism spectrum disorder. Curr Psychiatry Rep. (2017) 19:92. 10.1007/s11920-017-0846-y 29082426 PMC5846200

[86] Lai M-C Kassee C Besney R Bonato S Hull L Mandy W . Prevalence of co-occurring mental health diagnoses in the autism population: a systematic review and meta-analysis. Lancet Psychiatry. (2019) 6:819–29. 10.1016/S2215-0366(19)30289-5 31447415

[87] Lebowitz ER Panza KE Bloch MH. Family accommodation in obsessive-compulsive and anxiety disorders: a five-year update. Expert Rev Neurother. (2016) 16:45–53. 10.1586/14737175.2016.1126181 26613396 PMC4895189

[88] Weidle B Skarphedinsson G. Treatment of a child with obsessive-compulsive disorder with limited motivation: course and outcome of cognitive-behavior therapy. J Clin Psychol. (2016) 72:1139–51. 10.1002/jclp.22394 27716951

[89] Legerstee JS Dierckx B Utens EMWJ Verhulst FC Zieldorff C Dieleman GC . The age of onset of anxiety disorders. In: de Girolamo G McGorry PD Sartorius N, editors. Age of Onset of Mental Disorders. Cham: Springer International Publishing (2019). p. 125–147. 10.1007/978-3-319-72619-9_7

[90] Kelly AB Weier M Hall WD. The age of onset of substance use disorders. In: de Girolamo G McGorry PD Sartorius N, editor. Age of Onset of Mental Disorders. Cham: Springer International Publishing (2018). p. 149–67. 10.1007/978-3-319-72619-9_8

[91] Lochner C Seedat S Hemmings SMJ Kinnear CJ Corfield VA Niehaus DJH . Dissociative experiences in obsessive-compulsive disorder and trichotillomania: clinical and genetic findings. Compr Psychiatry. (2004) 45:384–91. 10.1016/j.comppsych.2004.03.010 15332202

[92] Ojserkis R Boisseau CL Reddy MK Mancebo MC Eisen JL Rasmussen SA. The impact of lifetime PTSD on the seven-year course and clinical characteristics of OCD. Psychiatry Res. (2017) 258:78–82. 10.1016/j.psychres.2017.09.042 28988123 PMC5681424

[93] Lochner C Hemmings SMJ Kinnear CJ Moolman-Smook JC Corfield VA Knowles JA . Gender in obsessive-compulsive disorder: clinical and genetic findings. Eur Neuropsychopharmacol. (2004) 14:105–13. 10.1016/S0924-977X(03)00063-4 15013025

[94] Torresan RC Ramos-Cerqueira ATA Shavitt RG do Rosário MC de Mathis MA Miguel EC . Symptom dimensions, clinical course and comorbidity in men and women with obsessive-compulsive disorder. Psychiatry Res. (2013) 209:186–95. 10.1016/j.psychres.2012.12.006 23298952

[95] Torresan RC de Abreu Ramos-Cerqueira AT de Mathis MA Diniz JB Ferrão YA Miguel EC . Sex differences in the phenotypic expression of obsessive-compulsive disorder: an exploratory study from Brazil. Compr Psychiatry. (2009) 50:63–9. 10.1016/j.comppsych.2008.05.005 19059516

[96] Conelea CA Woods DW Zinner SH Budman C Murphy T Scahill LD . Exploring the impact of chronic tic disorders on youth: results from the tourette syndrome impact survey. Child Psychiatry Hum Dev. (2011) 42:219–42. 10.1007/s10578-010-0211-4 21046225

[97] Lewin AB Chang S McCracken J McQueen M Piacentini J. Comparison of clinical features among youth with tic disorders, obsessive-compulsive disorder (OCD), and both conditions. Psychiatry Res. (2010) 178:317–22. 10.1016/j.psychres.2009.11.013 20488548 PMC2902642

[98] Bloch MH Craiglow BG Landeros-Weisenberger A Dombrowski PA Panza KE Peterson BS . Predictors of early adult outcomes in pediatric-onset obsessive-compulsive disorder. Pediatrics. (2009) 124:1085–93. 10.1542/peds.2009-0015 19786445 PMC3974608

[99] Friborg O Martinussen M Kaiser S Øvergård KT Rosenvinge JH. Comorbidity of personality disorders in anxiety disorders: a meta-analysis of 30 years of research. J Affect Disord. (2013) 145:143–55. 10.1016/j.jad.2012.07.004 22999891

[100] Starcevic V Berle D Brakoulias V Sammut P Moses K Milicevic D . Obsessive-compulsive personality disorder co-occurring with obsessive-compulsive disorder: conceptual and clinical implications. Aust N Z J Psychiatry. (2013) 47:65–73. 10.1177/0004867412450645 22689335

[101] Melca IA Yücel M Mendlowicz MV de Oliveira-Souza R Fontenelle LF. The correlates of obsessive-compulsive, schizotypal, and borderline personality disorders in obsessive-compulsive disorder. J Anxiety Disord. (2015) 33:15–24. 10.1016/j.janxdis.2015.04.004 25956558

[102] Lee PH Anttila V Won H Feng Y-CA Rosenthal J Zhu Z . Genomic relationships, novel loci, and pleiotropic mechanisms across eight psychiatric disorders. Cell. (2019) 179:1469–82.e11. 10.1016/j.cell.2019.11.020 31835028 PMC7077032

[103] Youngstrom EA Choukas-Bradley S Calhoun CD Jensen-Doss A. Clinical guide to the evidence-based assessment approach to diagnosis and treatment. Cogn Behav Pract. (2015) 22:20–35. 10.1016/j.cbpra.2013.12.00527322595

[104] Berkson J. Limitations of the application of fourfold table analysis to hospital data. Biometrics. (1946) 2:47–53. 10.2307/3002000 21001024

