# Supplementary material for: Comorbidities in Obsessive-Compulsive Disorder Across the Lifespan: A Systematic Review and Meta-Analysis
Source: Front Psychiatry. 2021 Nov 11;12:703701. doi: 10.3389/fpsyt.2021.703701 (PMC8631971; doi:10.3389/fpsyt.2021.703701)
Supplement: Supplementary file 1 [file Data_Sheet_1.PDF]

OCD Comorbidity Meta-analysis: Across the Lifespan

Sharma E et al

26/07/2021

Supplementary tables describing studies included in the meta-analysis for each comorbidity

Abbreviations used:

OCD - Obsessive-Compulsive Disorder; AAO - Age at Onset of OCD; (C)YBOCS-T - (Childhood) Yale-Brown Obsessive-Compulsive Scale Total Score; MINI - Mini International Neuropsychiatric Interview; SCID - Structured Clinical Interview for DSM; ADIS - Anxiety Disorders Interview Schedule; SADS - Schedule for affective Disorders and Schizophrenia; K-SADS - Kidddie-SADS; DIS - Diagnostic Interview Schedule

Suppl Table 1. Any psychiatric comorbidity

Total number of studies that reported Any psychiatric comorbidity = 23

Number of pediatric studies that reported Any psychiatric comorbidity = 5

Total N of OCD cases in studies that reported Any psychiatric comorbidity = 6272

Total cases of Any psychiatric comorbidity = 4328

No	Study	Country	Total OCD Subjects	Mean Age	Mean AAO	Age Group	% Males	Mean (C)YBOCS-T	Diagnostic Instrument	Any_AxisI_Life	Any_AxisI_Life %
1	Shavitt 2014	Brazil	1001	34.9	21.8	Adult	43.2	25.5	SCID-I	921	92.0
2	Cameron 2019	Canada	355	33.5	17.8	Adult	44.3	24.2	SCID-I	277	78.0
3	Adam 2019	Germany	181	13.2	NA	Pediatric	50.3	NA	Other	70	38.7
4	Dell'Osso 2015	ICOCS	483	43.3	19.4	Adult	39.9	23.1	MINI	88	18.2
5	Arumugham 2014	India	802	29.4	21.7	Adult	62.0	23.5	MINI-Plus	482	60.1
6	Albert 2015	Italy	483	34.4	22.1	Adult	52.0	25.0	SCID-I	381	78.9
7	Albert 2019	Italy	251	37.4	22.2	Adult	53.8	24.1	SCID-I	185	73.7
8	Benatti 2020	Italy	229	41.1	22.4	Adult	50.7	24.2	SCID-I and II	148	64.6
9	Dell'Osso 2017	Italy	124	40.2	23.3	Adult	50.0	25.8	SCID-I and II	77	62.1
10	Ronchi 1992	Italy	131	34.7	22.1	Adult	45.8	NA	DIS-R	71	54.2
11	Eilertsen 2017	Norway	204	31.0	NA	Adult	29.4	25.7	MINI	76	37.3
12	Ortiz 2016	Spain	112	14.7	12.1	Pediatric	50.9	17.5	KSADS-PL	75	67.0
13	Sevilla-Cermeno 2019	Sweden	193	13.6	10.8	Pediatric	44.6	22.6	MINI-KID	93	48.2
14	Tukel 2004	Turkey	169	28.7	20.2	Adult	43.2	25.3	SCID	130	76.9
15	Sahmelikoglu Onur 2016	Turkey	146	37.9	21.6	Adult	76.0	NA	SCID-I	141	96.6
16	Yovel 2012	United States	127	31.9	NA	Adult	49.6	NA	SCID	76	59.8
17	Garner 2018	United States	222	31.0	13.0	Adult	57.0	NA	SCID-I	186	83.8
18	Falkenstein 2019	United States	305	30.2	13.0	Adult	54.4	25.3	SCID-I and II	260	85.2
19	Kugler 2013	United States	102	29.4	NA	Adult	52.0	NA	ADIS-IV	74	72.5
20	Mancebo 2008	United States	238	41.1	18.0	Adult	43.7	23.3	SCID-I and II	214	89.9
21	Piacentini 2003	United States	151	11.8	7.9	Pediatric	57.0	NA	ADIS-IV & K-SADS-PL	103	68.2
22	Leonard 2014	United States	126	15.5	NA	Pediatric	47.6	24.9	Other	114	90.5
23	Fals-Stewart 1993	United States	137	30.5	NA	Adult	42.3	NA	DIS	86	62.8

Suppl Table 2. Any mood disorder

Total number of studies that reported Any mood disorder = 21

Number of pediatric studies that reported Any mood disorder = 4

Total N of OCD cases in studies that reported Any mood disorder = 6187

Total cases of Any mood disorder = 2991

No	Study	Country	Total OCD Subjects	Mean Age	Mean AAO	Age Group	% Males	Mean (C)YBOCS-T	Diagnostic Instrument	Any_mood_Life	Any_mood_Life %
1	deMathis 2008	Brazil	330	32.9	14.0	Adult	54.9	24.0	SCID-I	233	70.6
2	Shavitt 2014	Brazil	1001	34.9	21.8	Adult	43.2	25.5	SCID-I	426	42.6
3	Vorstenbosch 2012	Canada	180	32.9	18.0	Adult	37.2	NA	SCID	68	37.8
4	Jaspers-Fayer 2017	Canada	136	13.0	9.4	Pediatric	54.0	21.2	ADIS-IV	18	13.2
5	Lennertz 2013	Germany	232	36.7	20.1	Adult	47.4	NA	SADS & SCID	136	58.6
6	Arumugham 2014	India	802	29.4	21.7	Adult	62.0	23.5	MINI-Plus	482	60.1
7	Albert 2015	Italy	483	34.4	22.1	Adult	52.0	25.0	SCID-I	304	62.9
8	Maina 2008	Italy	148	34.8	22.8	Adult	50.7	26.2	SCID-I and II	117	79.1
9	Albert 2019	Italy	251	37.4	22.2	Adult	53.8	24.1	SCID-I	128	51.0
10	Benatti 2020	Italy	229	41.1	22.4	Adult	50.7	24.2	SCID-I and II	76	33.2
11	Hofmeijer-Sevink 2013	Netherlands	382	36.4	18.0	Adult	43.0	21.2	SCID-I	244	63.9
12	Denys 2004	Netherlands	420	36.0	19.8	Adult	38.0	24.2	MINI	114	27.1
13	Holm 2019	Norway	104	30.3	NA	Adult	26.9	26.0	MINI	29	27.9
14	Ortiz 2016	Spain	112	14.7	12.1	Pediatric	50.9	17.5	KSADS-PL	13	11.6
15	Ricciardi 1995	United States	150	35.8	18.5	Adult	45.3	21.6	SCID	86	57.3
16	Nota 2020	United States	173	29.0	13.2	Adult	NA	14.7	SCID-5	112	64.7
17	Kwee 2019	Netherlands	110	NA	NA	Adult	NA	21.5	SCID-I	51	46.4
18	Mancebo 2009	United States	323	40.1	18.5	Adult	45.0	20.6	SCID-I and II	240	74.3
19	Rozenman 2019	United States	215	12.2	NA	Pediatric	57.0	24.9	ADIS-IV	19	8.8
20	Leonard 2014	United States	126	15.5	NA	Pediatric	47.6	24.9	Other	93	73.8
21	Calamari 2008	USA & Canada	280	34.9	NA	Adult	46.8	25.7	SCID	2	0.7

Suppl Table 3. Major Depressive Disorder

Total number of studies that reported Major Depressive Disorder = 43

Number of pediatric studies that reported Major Depressive Disorder = 9

Total N of OCD cases in studies that reported Major Depressive Disorder = 9909

Total cases of Major Depressive Disorder = 3774

No	Study	Country	Total OCD Subjects	Mean Age	Mean AAO	Age Group	% Males	Mean (C)YBOCS-T	Diagnostic Instrument	MDD_Life	MDD_Life %
1	Brakoulias 2014	Australia	154	45.5	NA	Adult	44.1	22.0	MINI	25	16.2
2	deMathis 2008	Brazil	330	32.9	14.0	Adult	54.9	24.0	SCID-I	200	60.6
3	dosSantos-Ribeiro 2016	Brazil	1001	34.8	12.5	Adult	43.1	23.6	SCID	565	56.4
4	Nissen 2018	Denmark	317	13.9	8.9	Pediatric	36.0	24.5	KSADS-PL	18	5.7
5	Delorme 2005	France	161	23.9	12.6	Adult	55.3	NA	DIGS & KSADS-PL	58	36.0
6	Ecker 2014	Germany	170	39.5	NA	Adult	NA	NA	Other	71	41.8
7	Adam 2019	Germany	181	13.2	NA	Pediatric	50.3	NA	Other	13	7.2
8	Dell'osso 2020	ICOCS	401	38.6	19.2	Adult	39.4	25.4	SCID-I	165	41.1
9	Bhattacharyya 2005	India	218	28.5	21.3	Adult	67.0	NA	OPCRIT	38	17.4
10	Viswanath 2012	India	545	29.3	21.8	Adult	60.9	23.3	MINI-Plus	165	30.3
11	Maina 2008	Italy	148	34.8	22.8	Adult	50.7	26.2	SCID-I and II	34	23.0
12	Ronchi 1992	Italy	131	34.7	22.1	Adult	45.8	NA	DIS-R	42	32.1
13	Yanagisawa 2015	Japan	256	30.1	22.5	Adult	44.1	27.6	SCID-IV	120	46.9
14	Matsunaga 2008	Japan	343	30.4	22.6	Adult	41.4	26.9	SCID-I	151	44.0
15	Koh 2015	Korea	355	30.5	18.8	Adult	65.9	22.2	SCID	97	27.3
16	Camarena 2007	Mexico	168	25.9	20.4	Adult	49.5	23.9	DIS	118	70.2
17	Camarena 2001	Mexico	122	34.7	21.4	Adult	51.6	NA	DIS	89	73.0
18	vanOudheusden 2020	Netherlands	419	36.6	18.5	Adult	44.2	19.9	SCID-I	237	56.6
19	Denys 2004	Netherlands	420	36.0	19.8	Adult	38.0	24.2	MINI	87	20.7
20	Eilertsen 2017	Norway	204	31.0	NA	Adult	29.4	25.7	MINI	34	16.7
21	Lochner 2004	South Africa	110	35.2	18.1	Adult	32.7	NA	SCID-I	59	53.6
22	Boloc 2019	Spain	101	14.6	11.9	Pediatric	49.5	18.8	KSADS-PL	1	1.0
23	Sevilla-Cermeno 2019	Sweden	193	13.6	10.8	Pediatric	44.6	22.6	MINI-KID	25	13.0
24	Tukel 2002	Turkey	147	28.5	20.2	Adult	43.5	25.2	SCID	58	39.5
25	Tukel 2005	Turkey	116	29.6	20.3	Adult	41.4	NA	SCID-I	49	42.2
26	Sahmelikoglu Onur 2016	Turkey	146	37.9	21.6	Adult	76.0	NA	SCID-I	70	47.9
27	Castle 1995	UK	219	33.8	24.3	Adult	42.0	NA	Other	50	22.8
28	Ricciardi 1995	United States	150	35.8	18.5	Adult	45.3	21.6	SCID	59	39.3
29	Geller 2000	United States	187	11.9	7.4	Pediatric	69.5	NA	K-SADS-PL	125	66.8
30	Geller 2001	United States	101	NA	8.2	Pediatric	65.3	23.0	K-SADS-E	52	51.5
31	Yovel 2012	United States	127	31.9	NA	Adult	49.6	NA	SCID	40	31.5
32	Garner 2018	United States	222	31.0	13.0	Adult	57.0	NA	SCID-I	98	44.1
33	Reid 2017	United States	288	31.0	16.3	Adult	56.0	25.4	SCID-IV	181	62.8
34	Klein 2020	United States	290	34.5	NA	Adult	52.8	NA	MINI	55	19.0
35	Deacon 2005	United States	100	35.8	16.7	Adult	51.0	24.0	MINI	31	31.0
36	Kugler 2013	United States	102	29.4	NA	Adult	52.0	NA	ADIS-IV	37	36.3
37	Mancebo 2009	United States	323	40.1	18.5	Adult	45.0	20.6	SCID-I and II	216	66.9
38	Peris 2017	United States	322	12.3	NA	Pediatric	53.0	25.2	ADIS-IV	23	7.1
39	Piacentini 2003	United States	151	11.8	7.9	Pediatric	57.0	NA	ADIS-IV & K-SADS-PL	19	12.6
40	Karayiorgou 1999	United States	103	28.3	14.4	Adult	45.6	NA	DIGS	64	62.1
41	Leonard 2014	United States	126	15.5	NA	Pediatric	47.6	24.9	Other	24	19.0
42	Fals-Stewart 1993	United States	137	30.5	NA	Adult	42.3	NA	DIS	54	39.4
43	Merrill 2011	USA	104	NA	NA	Adult	NA	NA		57	54.8

Suppl Table 4. Dysthymia

Total number of studies that reported Dysthymia = 17

Number of pediatric studies that reported Dysthymia = 2

Total N of OCD cases in studies that reported Dysthymia = 4924

Total cases of Dysthymia = 586

No	Study	Country	Total OCD Subjects	Mean Age	Mean AAO	Age Group	% Males	Mean (C)YBOCS-T	Diagnostic Instrument	Dysthymia_Life	Dysthymia_Life %
1	dosSantos-Ribeiro 2016	Brazil	1001	34.8	12.5	Adult	43.1	23.6	SCID	118	11.8
2	Lennertz 2013	Germany	232	36.7	20.1	Adult	47.4	NA	SADS & SCID	91	39.2
3	Bhattacharyya 2005	India	218	28.5	21.3	Adult	67.0	NA	OPCRIT	12	5.5
4	Viswanath 2012	India	545	29.3	21.8	Adult	60.9	23.3	MINI-Plus	89	16.3
5	Maina 2008	Italy	148	34.8	22.8	Adult	50.7	26.2	SCID-I and II	10	6.8
6	Ronchi 1992	Italy	131	34.7	22.1	Adult	45.8	NA	DIS-R	3	2.3
7	vanOudheusden 2020	Netherlands	419	36.6	18.5	Adult	44.2	19.9	SCID-I	22	5.3
8	Denys 2004	Netherlands	420	36.0	19.8	Adult	38.0	24.2	MINI	12	2.9
9	Tukel 2004	Turkey	169	28.7	20.2	Adult	43.2	25.3	SCID	31	18.3
10	Tukel 2005	Turkey	116	29.6	20.3	Adult	41.4	NA	SCID-I	14	12.1
11	Sahmelikoglu Onur 2016	Turkey	146	37.9	21.6	Adult	76.0	NA	SCID-I	14	9.6
12	Ricciardi 1995	United States	150	35.8	18.5	Adult	45.3	21.6	SCID	19	12.7
13	Geller 2000	United States	187	11.9	7.4	Pediatric	69.5	NA	K-SADS-PL	20	10.7
14	Klein 2020	United States	290	34.5	NA	Adult	52.8	NA	MINI	32	11.0
15	Pinto 2006	United States	293	40.5	18.5	Adult	45.0	20.3	SCID-I and II	23	7.8
16	Peris 2017	United States	322	12.3	NA	Pediatric	53.0	25.2	ADIS-IV	6	1.9
17	Fals-Stewart 1993	United States	137	30.5	NA	Adult	42.3	NA	DIS	70	51.1

Suppl Table 5. Bipolar Disorder

Total number of studies that reported Bipolar Disorder = 22

Number of pediatric studies that reported Bipolar Disorder = 3

Total N of OCD cases in studies that reported Bipolar Disorder = 6158

Total cases of Bipolar Disorder = 359

No	Study	Country	Total OCD Subjects	Mean Age	Mean AAO	Age Group	% Males	Mean (C)YBOCS-T	Diagnostic Instrument	Bipolar_Life	Bipolar_Life %
1	deMathis 2008	Brazil	330	32.9	14.0	Adult	54.9	24.0	SCID-I	51	15.5
2	dosSantos-Ribeiro 2016	Brazil	1001	34.8	12.5	Adult	43.1	23.6	SCID	72	7.2
3	Dell'osso 2020	ICOCS	401	38.6	19.2	Adult	39.4	25.4	SCID-I	32	8.0
4	Viswanath 2012	India	545	29.3	21.8	Adult	60.9	23.3	MINI-Plus	15	2.8
5	Balachander 2020	India	420	29.5	19.3	Adult	59.8	29.9	MINI	33	7.9
6	Albert 2013	Italy	104	35.9	19.7	Adult	59.6	25.2	SCID-I	18	17.3
7	Maina 2008	Italy	148	34.8	22.8	Adult	50.7	26.2	SCID-I and II	19	12.8
8	Ronchi 1992	Italy	131	34.7	22.1	Adult	45.8	NA	DIS-R	5	3.8
9	vanOudheusden 2020	Netherlands	419	36.6	18.5	Adult	44.2	19.9	SCID-I	13	3.1
10	Denys 2004	Netherlands	420	36.0	19.8	Adult	38.0	24.2	MINI	4	1.0
11	vanderVeld 2020	Netherlands	400	35.1	NA	Adult	35.5	NA	MINI	1	0.2
12	Holm 2019	Norway	104	30.3	NA	Adult	26.9	26.0	MINI	1	1.0
13	Lochner 2004	South Africa	110	35.2	18.1	Adult	32.7	NA	SCID-I	4	3.6
14	Sevilla-Cermeno 2019	Sweden	193	13.6	10.8	Pediatric	44.6	22.6	MINI-KID	1	0.5
15	Tukel 2002	Turkey	147	28.5	20.2	Adult	43.5	25.2	SCID	5	3.4
16	Tukel 2005	Turkey	116	29.6	20.3	Adult	41.4	NA	SCID-I	9	7.8
17	Sahmelikoglu Onur 2016	Turkey	146	37.9	21.6	Adult	76.0	NA	SCID-I	11	7.5
18	Geller 2000	United States	187	11.9	7.4	Pediatric	69.5	NA	K-SADS-PL	43	23.0
19	Yovel 2012	United States	127	31.9	NA	Adult	49.6	NA	SCID	4	3.1
20	Klein 2020	United States	290	34.5	NA	Adult	52.8	NA	MINI	8	2.8
21	Pinto 2006	United States	293	40.5	18.5	Adult	45.0	20.3	SCID-I and II	8	2.7
22	Leonard 2014	United States	126	15.5	NA	Pediatric	47.6	24.9	Other	2	1.6

Suppl Table 6. Schizophrenia/Any psychotic disorder

Total number of studies that reported Schizophrenia/Any psychotic disorder = 13

Number of pediatric studies that reported Schizophrenia/Any psychotic disorder = 2

Total N of OCD cases in studies that reported Schizophrenia/Any psychotic disorder = 4642

Total cases of Schizophrenia/Any psychotic disorder = 177

No	Study	Country	Total OCD Subjects	Mean Age	Mean AAO	Age Group	% Males	Mean (C)YBOCS-T	Diagnostic Instrument	Psychosis_Life	Psychosis_Life %
1	dosSantos-Ribeiro 2016	Brazil	1001	34.8	12.5	Adult	43.1	23.6	SCID	9	0.9
2	Garyfallos 2010	Greece	146	33.6	23.7	Adult	43.8	21.9	MINI	3	2.1
3	Viswanath 2011	India	164	NA	22.2	Adult	64.6	23.8	MINI	5	3.0
4	Balachander 2020	India	420	29.5	19.3	Adult	59.8	29.9	MINI	35	8.3
5	Maina 2008	Italy	148	34.8	22.8	Adult	50.7	26.2	SCID-I and II	12	8.1
6	vanOudheusden 2020	Netherlands	419	36.6	18.5	Adult	44.2	19.9	SCID-I	18	4.3
7	DeHaan 2009	Netherlands	757	NA	21.9	Adult	37.7	23.9	SCID	13	1.7
8	Denys 2004	Netherlands	420	36.0	19.8	Adult	38.0	24.2	MINI	14	3.3
9	vanderVeld 2020	Netherlands	400	35.1	NA	Adult	35.5	NA	MINI	9	2.2
10	Tukel 2004	Turkey	169	28.7	20.2	Adult	43.2	25.3	SCID	12	7.1
11	Geller 2000	United States	187	11.9	7.4	Pediatric	69.5	NA	K-SADS-PL	27	14.4
12	Geller 2001	United States	101	NA	8.2	Pediatric	65.3	23.0	K-SADS-E	11	10.9
13	Pardue 2014	United States	310	40.4	18.6	Adult	45.5	NA	SCID-I	9	2.9

Suppl Table 7. Any anxiety disorder

Total number of studies that reported Any anxiety disorder = 26

Number of pediatric studies that reported Any anxiety disorder = 9

Total N of OCD cases in studies that reported Any anxiety disorder = 7236

Total cases of Any anxiety disorder = 2622

No	Study	Country	Total OCD Subjects	Mean Age	Mean AAO	Age Group	% Males	Mean (C)YBOCS-T	Diagnostic Instrument	Any_Anx_Life	Any_Anx_Life %
1	deMathis 2008	Brazil	330	32.9	14.0	Adult	54.9	24.0	SCID-I	211	63.9
2	Shavitt 2014	Brazil	1001	34.9	21.8	Adult	43.2	25.5	SCID-I	651	65.0
3	Vorstenbosch 2012	Canada	180	32.9	18.0	Adult	37.2	NA	SCID	115	63.9
4	Jaspers-Fayer 2017	Canada	136	13.0	9.4	Pediatric	54.0	21.2	ADIS-IV	50	36.8
5	Nissen 2018	Denmark	317	13.9	8.9	Pediatric	36.0	24.5	KSADS-PL	72	22.7
6	Lennertz 2013	Germany	232	36.7	20.1	Adult	47.4	NA	SADS & SCID	93	40.1
7	Bhattacharyya 2005	India	218	28.5	21.3	Adult	67.0	NA	OPCRIT	15	6.9
8	Viswanath 2012	India	545	29.3	21.8	Adult	60.9	23.3	MINI-Plus	114	20.9
9	Albert 2015	Italy	483	34.4	22.1	Adult	52.0	25.0	SCID-I	123	25.5
10	Maina 2008	Italy	148	34.8	22.8	Adult	50.7	26.2	SCID-I and II	58	39.2
11	Albert 2019	Italy	251	37.4	22.2	Adult	53.8	24.1	SCID-I	64	25.5
12	Benatti 2020	Italy	229	41.1	22.4	Adult	50.7	24.2	SCID-I and II	25	10.9
13	Yanagisawa 2015	Japan	256	30.1	22.5	Adult	44.1	27.6	SCID-IV	64	25.0
14	Koh 2015	Korea	355	30.5	18.8	Adult	65.9	22.2	SCID	51	14.4
15	Hofmeijer-Sevink 2013	Netherlands	382	36.4	18.0	Adult	43.0	21.2	SCID-I	176	46.1
16	Denys 2004	Netherlands	420	36.0	19.8	Adult	38.0	24.2	MINI	54	12.9
17	Ortiz 2016	Spain	112	14.7	12.1	Pediatric	50.9	17.5	KSADS-PL	41	36.6
18	Boloc 2019	Spain	101	14.6	11.9	Pediatric	49.5	18.8	KSADS-PL	24	23.8
19	Sevilla-Cermeno 2019	Sweden	193	13.6	10.8	Pediatric	44.6	22.6	MINI-KID	14	7.3
20	Kwee 2019	Netherlands	110	NA	NA	Adult	NA	21.5	SCID-I	46	41.8
21	Deacon 2005	United States	100	35.8	16.7	Adult	51.0	24.0	MINI	22	22.0
22	Mancebo 2009	United States	323	40.1	18.5	Adult	45.0	20.6	SCID-I and II	182	56.3
23	Rozenman 2017	United States	215	12.2	NA	Pediatric	57.0	24.9	ADIS-IV	108	50.2
24	Peris 2017	United States	322	12.3	NA	Pediatric	53.0	25.2	ADIS-IV	161	50.0
25	Piacentini 2003	United States	151	11.8	7.9	Pediatric	57.0	NA	ADIS-IV & K-SADS-PL	58	38.4
26	Leonard 2014	United States	126	15.5	NA	Pediatric	47.6	24.9	Other	30	23.8

Suppl Table 8. Generalised Anxiety Disorder

Total number of studies that reported Generalised Anxiety Disorder = 29

Number of pediatric studies that reported Generalised Anxiety Disorder = 6

Total N of OCD cases in studies that reported Generalised Anxiety Disorder = 7658

Total cases of Generalised Anxiety Disorder = 1399

No	Study	Country	Total OCD Subjects	Mean Age	Mean AAO	Age Group	% Males	Mean (C)YBOCS-T	Diagnostic Instrument	GAD_Life	GAD_Life %
1	Brakoulias 2014	Australia	154	45.5	NA	Adult	44.1	22.0	MINI	49	31.8
2	deMathis 2008	Brazil	330	32.9	14.0	Adult	54.9	24.0	SCID-I	85	25.8
3	dosSantos-Ribeiro 2016	Brazil	1001	34.8	12.5	Adult	43.1	23.6	SCID	343	34.3
4	Selles 2020	Canada	243	13.6	11.7	Pediatric	42.4	NA	ADIS-IV	54	22.2
5	Nissen 2018	Denmark	317	13.9	8.9	Pediatric	36.0	24.5	KSADS-PL	72	22.7
6	Delorme 2005	France	161	23.9	12.6	Adult	55.3	NA	DIGS & KSADS-PL	21	13.0
7	Arumugham 2014	India	802	29.4	21.7	Adult	62.0	23.5	MINI-Plus	43	5.4
8	Maina 2008	Italy	148	34.8	22.8	Adult	50.7	26.2	SCID-I and II	32	21.6
9	Milanfranchi 1995	Italy	154	34.2	22.7	Adult	38.9	NA	SCID-IV	25	16.2
10	vanOudheusden 2020	Netherlands	419	36.6	18.5	Adult	44.2	19.9	SCID-I	38	9.1
11	Denys 2004	Netherlands	420	36.0	19.8	Adult	38.0	24.2	MINI	4	1.0
12	Eilertsen 2017	Norway	204	31.0	NA	Adult	29.4	25.7	MINI	21	10.3
13	Holm 2019	Norway	104	30.3	NA	Adult	26.9	26.0	MINI	24	23.1
14	Lochner 2004	South Africa	110	35.2	18.1	Adult	32.7	NA	SCID-I	11	10.0
15	Tukel 2004	Turkey	169	28.7	20.2	Adult	43.2	25.3	SCID	19	11.2
16	Tukel 2005	Turkey	116	29.6	20.3	Adult	41.4	NA	SCID-I	14	12.1
17	Sahmelikoglu Onur 2016	Turkey	146	37.9	21.6	Adult	76.0	NA	SCID-I	15	10.3
18	Geller 2000	United States	187	11.9	7.4	Pediatric	69.5	NA	K-SADS-PL	80	42.8
19	Garner 2018	United States	222	31.0	13.0	Adult	57.0	NA	SCID-I	13	5.9
20	Reid 2017	United States	288	31.0	16.3	Adult	56.0	25.4	SCID-IV	37	12.8
21	Falkenstein 2019	United States	305	30.2	13.0	Adult	54.4	25.3	SCID-I and II	24	7.9
22	Nota 2020	United States	173	29.0	13.2	Adult	NA	14.7	SCID-5	29	16.8
23	Klein 2020	United States	290	34.5	NA	Adult	52.8	NA	MINI	45	15.5
24	Kugler 2013	United States	102	29.4	NA	Adult	52.0	NA	ADIS-IV	39	38.2
25	Pinto 2006	United States	293	40.5	18.5	Adult	45.0	20.3	SCID-I and II	22	7.5
26	Rozenman 2017	United States	215	12.2	NA	Pediatric	57.0	24.9	ADIS-IV	69	32.1
27	Peris 2017	United States	322	12.3	NA	Pediatric	53.0	25.2	ADIS-IV	103	32.0
28	Leonard 2014	United States	126	15.5	NA	Pediatric	47.6	24.9	Other	14	11.1
29	Fals-Stewart 1993	United States	137	30.5	NA	Adult	42.3	NA	DIS	54	39.4

Suppl Table 9. Social Anxiety Disorder

Total number of studies that reported Social Anxiety Disorder = 28

Number of pediatric studies that reported Social Anxiety Disorder = 6

Total N of OCD cases in studies that reported Social Anxiety Disorder = 6716

Total cases of Social Anxiety Disorder = 1209

No	Study	Country	Total OCD Subjects	Mean Age	Mean AAO	Age Group	% Males	Mean (C)YBOCS-T	Diagnostic Instrument	SAD_Life	SAD_Life %
1	Brakoulias 2014	Australia	154	45.5	NA	Adult	44.1	22.0	MINI	25	16.2
2	deMathis 2008	Brazil	330	32.9	14.0	Adult	54.9	24.0	SCID-I	124	37.6
3	dosSantos-Ribeiro 2016	Brazil	1001	34.8	12.5	Adult	43.1	23.6	SCID	345	34.5
4	Selles 2020	Canada	243	13.6	11.7	Pediatric	42.4	NA	ADIS-IV	22	9.1
5	Delorme 2005	France	161	23.9	12.6	Adult	55.3	NA	DIGS & KSADS-PL	28	17.4
6	Viswanath 2011	India	164	NA	22.2	Adult	64.6	23.8	MINI	7	4.3
7	Maina 2008	Italy	148	34.8	22.8	Adult	50.7	26.2	SCID-I and II	11	7.4
8	vanOudheusden 2020	Netherlands	419	36.6	18.5	Adult	44.2	19.9	SCID-I	97	23.2
9	Denys 2004	Netherlands	420	36.0	19.8	Adult	38.0	24.2	MINI	15	3.6
10	Holm 2019	Norway	104	30.3	NA	Adult	26.9	26.0	MINI	7	6.7
11	Lochner 2004	South Africa	110	35.2	18.1	Adult	32.7	NA	SCID-I	13	11.8
12	Tukel 2004	Turkey	169	28.7	20.2	Adult	43.2	25.3	SCID	26	15.4
13	Tukel 2005	Turkey	116	29.6	20.3	Adult	41.4	NA	SCID-I	13	11.2
14	Sahmelikoglu Onur 2016	Turkey	146	37.9	21.6	Adult	76.0	NA	SCID-I	27	18.5
15	Geller 2000	United States	187	11.9	7.4	Pediatric	69.5	NA	K-SADS-PL	37	19.8
16	Geller 2001	United States	101	NA	8.2	Pediatric	65.3	23.0	K-SADS-E	14	13.9
17	Yovel 2012	United States	127	31.9	NA	Adult	49.6	NA	SCID	11	8.7
18	Garner 2018	United States	222	31.0	13.0	Adult	57.0	NA	SCID-I	18	8.1
19	Reid 2017	United States	288	31.0	16.3	Adult	56.0	25.4	SCID-IV	63	21.9
20	Falkenstein 2019	United States	305	30.2	13.0	Adult	54.4	25.3	SCID-I and II	27	8.9
21	Nota 2020	United States	173	29.0	13.2	Adult	NA	14.7	SCID-5	39	22.5

22	Klein 2020	United States	290	34.5	NA	Adult	52.8	NA	MINI	20	6.9
23	Kugler 2013	United States	102	29.4	NA	Adult	52.0	NA	ADIS-IV	32	31.4
24	Pinto 2006	United States	293	40.5	18.5	Adult	45.0	20.3	SCID-I and II	81	27.6
25	Rozenman 2017	United States	215	12.2	NA	Pediatric	57.0	24.9	ADIS-IV	34	15.8
26	Peris 2017	United States	322	12.3	NA	Pediatric	53.0	25.2	ADIS-IV	48	14.9
27	Leonard 2014	United States	126	15.5	NA	Pediatric	47.6	24.9	Other	11	8.7
28	Calamari 2008	USA & Canada	280	34.9	NA	Adult	46.8	25.7	SCID	14	5.0

————————————————————————-

Suppl Table 10. Panic Disorder

Total number of studies that reported Panic Disorder = 25

Number of pediatric studies that reported Panic Disorder = 5

Total N of OCD cases in studies that reported Panic Disorder = 6180

Total cases of Panic Disorder = 663

No	Study	Country	Total OCD Subjects	Mean Age	Mean AAO	Age Group	% Males	Mean (C)YBOCS-T	Diagnostic Instrument	Panic_Life	Panic_Life %
1	Brakoulias 2014	Australia	154	45.5	NA	Adult	44.1	22.0	MINI	21	13.6
2	deMathis 2008	Brazil	330	32.9	14.0	Adult	54.9	24.0	SCID-I	58	17.6
3	dosSantos-Ribeiro 2016	Brazil	1001	34.8	12.5	Adult	43.1	23.6	SCID	156	15.6
4	Delorme 2005	France	161	23.9	12.6	Adult	55.3	NA	DIGS & KSADS-PL	12	7.5
5	Viswanath 2012	India	545	29.3	21.8	Adult	60.9	23.3	MINI-Plus	10	1.8
6	Maina 2008	Italy	148	34.8	22.8	Adult	50.7	26.2	SCID-I and II	12	8.1
7	Ronchi 1992	Italy	131	34.7	22.1	Adult	45.8	NA	DIS-R	7	5.3
8	Milanfranchi 1995	Italy	154	34.2	22.7	Adult	38.9	NA	SCID-IV	18	11.7
9	vanOudheusden 2020	Netherlands	419	36.6	18.5	Adult	44.2	19.9	SCID-I	94	22.4
10	Denys 2004	Netherlands	420	36.0	19.8	Adult	38.0	24.2	MINI	17	4.0
11	Holm 2019	Norway	104	30.3	NA	Adult	26.9	26.0	MINI	12	11.5
12	Lochner 2004	South Africa	110	35.2	18.1	Adult	32.7	NA	SCID-I	20	18.2
13	Tukel 2004	Turkey	169	28.7	20.2	Adult	43.2	25.3	SCID	15	8.9
14	Tukel 2005	Turkey	116	29.6	20.3	Adult	41.4	NA	SCID-I	8	6.9
15	Sahmelikoglu Onur 2016	Turkey	146	37.9	21.6	Adult	76.0	NA	SCID-I	33	22.6
16	Geller 2000	United States	187	11.9	7.4	Pediatric	69.5	NA	K-SADS-PL	34	18.2
17	Geller 2001	United States	101	NA	8.2	Pediatric	65.3	23.0	K-SADS-E	21	20.8
18	Yovel 2012	United States	127	31.9	NA	Adult	49.6	NA	SCID	6	4.7
19	Klein 2020	United States	290	34.5	NA	Adult	52.8	NA	MINI	18	6.2
20	Pinto 2006	United States	293	40.5	18.5	Adult	45.0	20.3	SCID-I and II	54	18.4
21	Langley 2010	United States	209	11.8	NA	Pediatric	57.0	NA	ADIS-IV & K-SADS-PL	1	0.5
22	Peris 2017	United States	322	12.3	NA	Pediatric	53.0	25.2	ADIS-IV	2	0.6
23	Leonard 2014	United States	126	15.5	NA	Pediatric	47.6	24.9	Other	4	3.2
24	Fals-Stewart 1993	United States	137	30.5	NA	Adult	42.3	NA	DIS	24	17.5
25	Calamari 2008	USA & Canada	280	34.9	NA	Adult	46.8	25.7	SCID	6	2.1

————————————————————————-

Suppl Table 11. Agoraphobia

Total number of studies that reported Agoraphobia = 9

Number of pediatric studies that reported Agoraphobia = 2

Total N of OCD cases in studies that reported Agoraphobia = 3237

Total cases of Agoraphobia = 142

No	Study	Country	Total OCD Subjects	Mean Age	Mean AAO	Age Group	% Males	Mean (C)YBOCS-T	Diagnostic Instrument	Agoraph_Life	Agoraph_Life %
1	dosSantos-Ribeiro 2016	Brazil	1001	34.8	12.5	Adult	43.1	23.6	SCID	49	4.9
2	Selles 2020	Canada	243	13.6	11.7	Pediatric	42.4	NA	ADIS-IV	3	1.2
3	Viswanath 2012	India	545	29.3	21.8	Adult	60.9	23.3	MINI-Plus	4	0.7
4	Hofmeijer-Sevink 2013	Netherlands	382	36.4	18.0	Adult	43.0	21.2	SCID-I	10	2.6
5	Tukel 2004	Turkey	169	28.7	20.2	Adult	43.2	25.3	SCID	6	3.6
6	Geller 2000	United States	187	11.9	7.4	Pediatric	69.5	NA	K-SADS-PL	56	29.9
7	Yovel 2012	United States	127	31.9	NA	Adult	49.6	NA	SCID	1	0.8
8	Klein 2020	United States	290	34.5	NA	Adult	52.8	NA	MINI	9	3.1
9	Pinto 2006	United States	293	40.5	18.5	Adult	45.0	20.3	SCID-I and II	4	1.4

————————————————————————-

Suppl Table 12. Simple/Specific Phobia

Total number of studies that reported Simple/Specific Phobia = 18

Number of pediatric studies that reported Simple/Specific Phobia = 5

Total N of OCD cases in studies that reported Simple/Specific Phobia = 5244

Total cases of Simple/Specific Phobia = 914

No	Study	Country	Total OCD Subjects	Mean Age	Mean AAO	Age Group	% Males	Mean (C)YBOCS-T	Diagnostic Instrument	Phobia_Life	Phobia_Life %
1	Brakoulias 2014	Australia	154	45.5	NA	Adult	44.1	22.0	MINI	32	20.8
2	deMathis 2008	Brazil	330	32.9	14.0	Adult	54.9	24.0	SCID-I	86	26.1
3	dosSantos-Ribeiro 2016	Brazil	1001	34.8	12.5	Adult	43.1	23.6	SCID	314	31.4
4	Selles 2020	Canada	243	13.6	11.7	Pediatric	42.4	NA	ADIS-IV	11	4.5
5	Delorme 2005	France	161	23.9	12.6	Adult	55.3	NA	DIGS & KSADS-PL	36	22.4
6	Viswanath 2012	India	545	29.3	21.8	Adult	60.9	23.3	MINI-Plus	71	13.0
7	Maina 2008	Italy	148	34.8	22.8	Adult	50.7	26.2	SCID-I and II	21	14.2
8	vanOudheusden 2020	Netherlands	419	36.6	18.5	Adult	44.2	19.9	SCID-I	43	10.3
9	Denys 2004	Netherlands	420	36.0	19.8	Adult	38.0	24.2	MINI	4	1.0
10	Tukel 2004	Turkey	169	28.7	20.2	Adult	43.2	25.3	SCID	27	16.0
11	Tukel 2005	Turkey	116	29.6	20.3	Adult	41.4	NA	SCID-I	13	11.2
12	Sahmelikoglu Onur 2016	Turkey	146	37.9	21.6	Adult	76.0	NA	SCID-I	78	53.4
13	Geller 2000	United States	187	11.9	7.4	Pediatric	69.5	NA	K-SADS-PL	54	28.9
14	Geller 2001	United States	101	NA	8.2	Pediatric	65.3	23.0	K-SADS-E	28	27.7
15	Pinto 2006	United States	293	40.5	18.5	Adult	45.0	20.3	SCID-I and II	53	18.1
16	Langley 2010	United States	209	11.8	NA	Pediatric	57.0	NA	ADIS-IV & K-SADS-PL	8	3.8
17	Peris 2017	United States	322	12.3	NA	Pediatric	53.0	25.2	ADIS-IV	29	9.0
18	Calamari 2008	USA & Canada	280	34.9	NA	Adult	46.8	25.7	SCID	6	2.1

————————————————————————-

Suppl Table 13. Somatic symptom disorder/Somatoform/Somatization disorder

Total number of studies that reported Somatic symptom disorder/Somatoform/Somatization disorder = 11

Number of pediatric studies that reported Somatic symptom disorder/Somatoform/Somatization disorder = 0

Total N of OCD cases in studies that reported Somatic symptom disorder/Somatoform/Somatization disorder = 3839

Total cases of Somatic symptom disorder/Somatoform/Somatization disorder = 302

No	Study	Country	Total OCD Subjects	Mean Age	Mean AAO	Age Group	% Males	Mean (C)YBOCS-T	Diagnostic Instrument	Somat_Life	Somat_Life %
1	deMathis 2008	Brazil	330	32.9	14.0	Adult	54.9	24.0	SCID-I	6	1.8
2	Shavitt 2014	Brazil	1001	34.9	21.8	Adult	43.2	25.5	SCID-I	167	16.7
3	Ecker 2014	Germany	170	39.5	NA	Adult	NA	NA	Other	18	10.6
4	Lennertz 2013	Germany	232	36.7	20.1	Adult	47.4	NA	SADS & SCID	12	5.2
5	Benatti 2020	Italy	229	41.1	22.4	Adult	50.7	24.2	SCID-I and II	1	0.4
6	vanOudheusden 2020	Netherlands	419	36.6	18.5	Adult	44.2	19.9	SCID-I	22	5.3
7	Denys 2004	Netherlands	420	36.0	19.8	Adult	38.0	24.2	MINI	16	3.8
8	vanderVeld 2020	Netherlands	400	35.1	NA	Adult	35.5	NA	MINI	12	3.0
9	Tukel 2004	Turkey	169	28.7	20.2	Adult	43.2	25.3	SCID	2	1.2
10	Sahmelikoglu Onur 2016	Turkey	146	37.9	21.6	Adult	76.0	NA	SCID-I	18	12.3
11	Mancebo 2009	United States	323	40.1	18.5	Adult	45.0	20.6	SCID-I and II	28	8.7

————————————————————————-

Suppl Table 14. Hypochondriasis/Illness anxiety disorder

Total number of studies that reported Hypochondriasis/Illness anxiety disorder = 7

Number of pediatric studies that reported Hypochondriasis/Illness anxiety disorder = 0

Total N of OCD cases in studies that reported Hypochondriasis/Illness anxiety disorder = 1971

Total cases of Hypochondriasis/Illness anxiety disorder = 43

No	Study	Country	Total OCD Subjects	Mean Age	Mean AAO	Age Group	% Males	Mean (C)YBOCS-T	Diagnostic Instrument	Hypoch_Life	Hypoch_Life %
1	deMathis 2008	Brazil	330	32.9	14.0	Adult	54.9	24.0	SCID-I	9	2.7
2	Viswanath 2012	India	545	29.3	21.8	Adult	60.9	23.3	MINI-Plus	4	0.7
3	Denys 2004	Netherlands	420	36.0	19.8	Adult	38.0	24.2	MINI	12	2.9
4	Holm 2019	Norway	104	30.3	NA	Adult	26.9	26.0	MINI	1	1.0
5	Tukel 2004	Turkey	169	28.7	20.2	Adult	43.2	25.3	SCID	6	3.6
6	Kwee 2019	Netherlands	110	NA	NA	Adult	NA	21.5	SCID-I	7	6.4
7	Pinto 2006	United States	293	40.5	18.5	Adult	45.0	20.3	SCID-I and II	4	1.4

————————————————————————-

Suppl Table 15. Post-traumatic stress disorder (PTSD)

Total number of studies that reported Post-traumatic stress disorder (PTSD) = 16

Number of pediatric studies that reported Post-traumatic stress disorder (PTSD) = 2

Total N of OCD cases in studies that reported Post-traumatic stress disorder (PTSD) = 4167

Total cases of Post-traumatic stress disorder (PTSD) = 385

No	Study	Country	Total OCD Subjects	Mean Age	Mean AAO	Age Group	% Males	Mean (C)YBOCS-T	Diagnostic Instrument	PTSD_Life	PTSD_Life %
1	deMathis 2008	Brazil	330	32.9	14.0	Adult	54.9	24.0	SCID-I	15	4.5
2	dosSantos-Ribeiro 2016	Brazil	1001	34.8	12.5	Adult	43.1	23.6	SCID	190	19.0
3	Lennertz 2013	Germany	232	36.7	20.1	Adult	47.4	NA	SADS & SCID	19	8.2
4	vanOudheusden 2020	Netherlands	419	36.6	18.5	Adult	44.2	19.9	SCID-I	19	4.5
5	Denys 2004	Netherlands	420	36.0	19.8	Adult	38.0	24.2	MINI	7	1.7
6	Holm 2019	Norway	104	30.3	NA	Adult	26.9	26.0	MINI	1	1.0
7	Lochner 2004	South Africa	110	35.2	18.1	Adult	32.7	NA	SCID-I	5	4.5
8	Sahmelikoglu Onur 2016	Turkey	146	37.9	21.6	Adult	76.0	NA	SCID-I	5	3.4
9	Yovel 2012	United States	127	31.9	NA	Adult	49.6	NA	SCID	18	14.2
10	Nota 2020	United States	173	29.0	13.2	Adult	NA	14.7	SCID-5	16	9.2
11	Klein 2020	United States	290	34.5	NA	Adult	52.8	NA	MINI	8	2.8
12	Kwee 2019	Netherlands	110	NA	NA	Adult	NA	21.5	SCID-I	6	5.5
13	Ojserkis 2017	United States	266	39.2	16.8	Adult	44.0	23.6	SCID-I	21	7.9
14	Langley 2010	United States	209	11.8	NA	Pediatric	57.0	NA	ADIS-IV & K-SADS-PL	1	0.5
15	Leonard 2014	United States	126	15.5	NA	Pediatric	47.6	24.9	Other	2	1.6
16	Merrill 2011	USA	104	NA	NA	Adult	NA	NA		52	50.0

————————————————————————-

Suppl Table 16. Any obsessive compulsive related (spectrum) disorders

Total number of studies that reported Any obsessive compulsive related (spectrum) disorders = 5

Number of pediatric studies that reported Any obsessive compulsive related (spectrum) disorders = 1

Total N of OCD cases in studies that reported Any obsessive compulsive related (spectrum) disorders = 1624

Total cases of Any obsessive compulsive related (spectrum) disorders = 283

No	Study	Country	Total OCD Subjects	Mean Age	Mean AAO	Age Group	% Males	Mean (C)YBOCS-T	Diagnostic Instrument	Any_OCRD_Life	Any_OCRD_Life %
1	Velloso 2016	Brazil	548	34.3	21.0	Adult	46.0	NA	SCID-I	175	31.9
2	Lennertz 2013	Germany	232	36.7	20.1	Adult	47.4	NA	SADS & SCID	24	10.3
3	Viswanath 2012	India	545	29.3	21.8	Adult	60.9	23.3	MINI-Plus	33	6.1
4	Nota 2020	United States	173	29.0	13.2	Adult	NA	14.7	SCID-5	48	27.7
5	Leonard 2014	United States	126	15.5	NA	Pediatric	47.6	24.9	Other	3	2.4

————————————————————————-

Suppl Table 17. Body dysmorphic disorder

Total number of studies that reported Body dysmorphic disorder = 6

Number of pediatric studies that reported Body dysmorphic disorder = 1

Total N of OCD cases in studies that reported Body dysmorphic disorder = 1561

Total cases of Body dysmorphic disorder = 84

No	Study	Country	Total OCD Subjects	Mean Age	Mean AAO	Age Group	% Males	Mean (C)YBOCS-T	Diagnostic Instrument	BDD_Life	BDD_Life %
1	deMathis 2008	Brazil	330	32.9	14.0	Adult	54.9	24.0	SCID-I	48	14.5
2	Viswanath 2012	India	545	29.3	21.8	Adult	60.9	23.3	MINI-Plus	3	0.6
3	Lochner 2004	South Africa	110	35.2	18.1	Adult	32.7	NA	SCID-I	3	2.7
4	Yovel 2012	United States	127	31.9	NA	Adult	49.6	NA	SCID	5	3.9
5	Mancebo 2009	United States	323	40.1	18.5	Adult	45.0	20.6	SCID-I and II	23	7.1
6	Leonard 2014	United States	126	15.5	NA	Pediatric	47.6	24.9	Other	2	1.6

————————————————————————-

Suppl Table 18. Trichotillomania

Total number of studies that reported Trichotillomania = 5

Number of pediatric studies that reported Trichotillomania = 2

Total N of OCD cases in studies that reported Trichotillomania = 1102

Total cases of Trichotillomania = 47

No	Study	Country	Total OCD Subjects	Mean Age	Mean AAO	Age Group	% Males	Mean (C)YBOCS-T	Diagnostic Instrument	TTM_Life	TTM_Life %
1	deMathis 2008	Brazil	330	32.9	14.0	Adult	54.9	24.0	SCID-I	26	7.9
2	Selles 2020	Canada	243	13.6	11.7	Pediatric	42.4	NA	ADIS-IV	6	2.5
3	Lochner 2004	South Africa	110	35.2	18.1	Adult	32.7	NA	SCID-I	10	9.1
4	Grant 2006	United States	293	40.6	18.5	Adult	45.4	20.3	SCID-I and II	4	1.4
5	Leonard 2014	United States	126	15.5	NA	Pediatric	47.6	24.9	Other	1	0.8

————————————————————————-

Suppl Table 19. Any tic disorder

Total number of studies that reported Any tic disorder = 31

Number of pediatric studies that reported Any tic disorder = 12

Total N of OCD cases in studies that reported Any tic disorder = 7367

Total cases of Any tic disorder = 1094

No	Study	Country	Total OCD Subjects	Mean Age	Mean AAO	Age Group	% Males	Mean (C)YBOCS-T	Diagnostic Instrument	Any_Tic_Life	Any_Tic_Life %
1	Brakoulias 2014	Australia	154	45.5	NA	Adult	44.1	22.0	MINI	17	11.0
2	deMathis 2008	Brazil	330	32.9	14.0	Adult	54.9	24.0	SCID-I	91	27.6
3	Velloso 2016	Brazil	548	34.3	21.0	Adult	46.0	NA	SCID-I	148	27.0
4	Jaspers-Fayer 2017	Canada	136	13.0	9.4	Pediatric	54.0	21.2	ADIS-IV	31	22.8
5	Selles 2020	Canada	243	13.6	11.7	Pediatric	42.4	NA	ADIS-IV	18	7.4
6	Bo 2013	China	241	29.3	19.8	Adult	62.2	NA	SCID	13	5.4
7	Nissen 2018	Denmark	317	13.9	8.9	Pediatric	36.0	24.5	KSADS-PL	39	12.3
8	Adam 2019	Germany	181	13.2	NA	Pediatric	50.3	NA	Other	19	10.5
9	Lennertz 2013	Germany	232	36.7	20.1	Adult	47.4	NA	SADS & SCID	17	7.3
10	Dell'Osso 2016	ICOCS	431	43.1	19.2	Adult	42.0	20.6	SCID-I and II	67	15.5
11	Viswanath 2012	India	545	29.3	21.8	Adult	60.9	23.3	MINI-Plus	21	3.9
12	Albert 2015	Italy	483	34.4	22.1	Adult	52.0	25.0	SCID-I	36	7.5
13	Benatti 2020	Italy	229	41.1	22.4	Adult	50.7	24.2	SCID-I and II	12	5.2
14	Ronchi 1992	Italy	131	34.7	22.1	Adult	45.8	NA	DIS-R	16	12.2
15	Camarena 2007	Mexico	168	25.9	20.4	Adult	49.5	23.9	DIS	38	22.6
16	vanOudheusden 2020	Netherlands	419	36.6	18.5	Adult	44.2	19.9	SCID-I	113	27.0
17	Denys 2006	The Netherlands	159	36.0	17.7	Adult	37.0	24.9	MINI	9	5.7
18	Katerberg 2010	Netherlands	147	36.1	15.3	Adult	45.6	21.0	SCID-I	65	44.2
19	Lochner 2004	South Africa	110	35.2	18.1	Adult	32.7	NA	SCID-I	14	12.7
20	Lochner 2004	South Africa	220	36.4	18.9	Adult	48.6	20.2	SCID-I and II	28	12.7
21	Ortiz 2016	Spain	112	14.7	12.1	Pediatric	50.9	17.5	KSADS-PL	13	11.6
22	Boloc 2019	Spain	101	14.6	11.9	Pediatric	49.5	18.8	KSADS-PL	11	10.9
23	Sevilla-Cermeno 2019	Sweden	193	13.6	10.8	Pediatric	44.6	22.6	MINI-KID	12	6.2
24	Cervin 2020	Sweden	120	13.3	8.4	Pediatric	37.5	23.2	MINI-KID	33	27.5
25	Mancebo 2009	United States	323	40.1	18.5	Adult	45.0	20.6	SCID-I and II	49	15.2
26	Rozenman 2019	United States	215	12.2	NA	Pediatric	57.0	24.9	ADIS-IV	19	8.8
27	Peris 2017	United States	322	12.3	NA	Pediatric	53.0	25.2	ADIS-IV	35	10.9
28	Piacentini 2003	United States	151	11.8	7.9	Pediatric	57.0	NA	ADIS-IV & K-SADS-PL	19	12.6
29	Leckman 1994	United States	177	39.1	15.1	Adult	37.9	17.5	Other	56	31.6
30	Karayiorgou 1999	United States	103	28.3	14.4	Adult	45.6	NA	DIGS	24	23.3
31	Leonard 2014	United States	126	15.5	NA	Pediatric	47.6	24.9	Other	11	8.7

————————————————————————-

Suppl Table 20. Any eating disorder

Total number of studies that reported Any eating disorder = 17

Number of pediatric studies that reported Any eating disorder = 3

Total N of OCD cases in studies that reported Any eating disorder = 5298

Total cases of Any eating disorder = 296

No	Study	Country	Total OCD Subjects	Mean Age	Mean AAO	Age Group	% Males	Mean (C)YBOCS-T	Diagnostic Instrument	Any_Eating_Life	Any_Eating_Life %
1	deMathis 2008	Brazil	330	32.9	14.0	Adult	54.9	24.0	SCID-I	33	10.0
2	Shavitt 2014	Brazil	1001	34.9	21.8	Adult	43.2	25.5	SCID-I	71	7.1
3	Nissen 2018	Denmark	317	13.9	8.9	Pediatric	36.0	24.5	KSADS-PL	22	6.9
4	Lennertz 2013	Germany	232	36.7	20.1	Adult	47.4	NA	SADS & SCID	14	6.0
5	Garyfallos 2010	Greece	146	33.6	23.7	Adult	43.8	21.9	MINI	7	4.8
6	Viswanath 2012	India	545	29.3	21.8	Adult	60.9	23.3	MINI-Plus	1	0.2
7	Maina 2008	Italy	148	34.8	22.8	Adult	50.7	26.2	SCID-I and II	8	5.4
8	Albert 2019	Italy	251	37.4	22.2	Adult	53.8	24.1	SCID-I	10	4.0
9	Benatti 2020	Italy	229	41.1	22.4	Adult	50.7	24.2	SCID-I and II	5	2.2
10	vanOudheusden 2020	Netherlands	419	36.6	18.5	Adult	44.2	19.9	SCID-I	44	10.5
11	Denys 2004	Netherlands	420	36.0	19.8	Adult	38.0	24.2	MINI	10	2.4
12	vanderVeld 2020	Netherlands	400	35.1	NA	Adult	35.5	NA	MINI	13	3.2
13	Holm 2019	Norway	104	30.3	NA	Adult	26.9	26.0	MINI	3	2.9
14	Boloc 2019	Spain	101	14.6	11.9	Pediatric	49.5	18.8	KSADS-PL	9	8.9
15	Castle 1995	UK	219	33.8	24.3	Adult	42.0	NA	Other	9	4.1
16	Pardue 2014	United States	310	40.4	18.6	Adult	45.5	NA	SCID-I	29	9.4
17	Leonard 2014	United States	126	15.5	NA	Pediatric	47.6	24.9	Other	8	6.3

————————————————————————-

Suppl Table 21. Anorexia nervosa

Total number of studies that reported Anorexia nervosa = 9

Number of pediatric studies that reported Anorexia nervosa = 4

Total N of OCD cases in studies that reported Anorexia nervosa = 1790

Total cases of Anorexia nervosa = 65

No	Study	Country	Total OCD Subjects	Mean Age	Mean AAO	Age Group	% Males	Mean (C)YBOCS-T	Diagnostic Instrument	Anorexia_Life	Anorexia_Life %
1	deMathis 2008	Brazil	330	32.9	14.0	Adult	54.9	24.0	SCID-I	11	3.3
2	Selles 2020	Canada	243	13.6	11.7	Pediatric	42.4	NA	ADIS-IV	2	0.8
3	Maina 2008	Italy	148	34.8	22.8	Adult	50.7	26.2	SCID-I and II	2	1.4
4	Ronchi 1992	Italy	131	34.7	22.1	Adult	45.8	NA	DIS-R	5	3.8
5	Lochner 2004	South Africa	220	36.4	18.9	Adult	48.6	20.2	SCID-I and II	20	9.1
6	Ortiz 2016	Spain	112	14.7	12.1	Pediatric	50.9	17.5	KSADS-PL	11	9.8
7	Geller 2000	United States	187	11.9	7.4	Pediatric	69.5	NA	K-SADS-PL	1	0.5
8	Pinto 2006	United States	293	40.5	18.5	Adult	45.0	20.3	SCID-I and II	9	3.1
9	Leonard 2014	United States	126	15.5	NA	Pediatric	47.6	24.9	Other	4	3.2

————————————————————————-

Suppl Table 22. Bulimia nervosa

Total number of studies that reported Bulimia nervosa = 7

Number of pediatric studies that reported Bulimia nervosa = 1

Total N of OCD cases in studies that reported Bulimia nervosa = 1418

Total cases of Bulimia nervosa = 35

No	Study	Country	Total OCD Subjects	Mean Age	Mean AAO	Age Group	% Males	Mean (C)YBOCS-T	Diagnostic Instrument	Bulemia_Life	Bulemia_Life %
1	deMathis 2008	Brazil	330	32.9	14.0	Adult	54.9	24.0	SCID-I	9	2.7
2	Maina 2008	Italy	148	34.8	22.8	Adult	50.7	26.2	SCID-I and II	3	2.0
3	Lochner 2004	South Africa	220	36.4	18.9	Adult	48.6	20.2	SCID-I and II	10	4.5
4	Ortiz 2016	Spain	112	14.7	12.1	Pediatric	50.9	17.5	KSADS-PL	1	0.9
5	Tukel 2004	Turkey	169	28.7	20.2	Adult	43.2	25.3	SCID	1	0.6
6	Sahmelikoglu Onur 2016	Turkey	146	37.9	21.6	Adult	76.0	NA	SCID-I	2	1.4
7	Pinto 2006	United States	293	40.5	18.5	Adult	45.0	20.3	SCID-I and II	9	3.1

————————————————————————-

Suppl Table 23. Any substance use disorder

Total number of studies that reported Any substance use disorder = 17

Number of pediatric studies that reported Any substance use disorder = 1

Total N of OCD cases in studies that reported Any substance use disorder = 5334

Total cases of Any substance use disorder = 446

No	Study	Country	Total OCD Subjects	Mean Age	Mean AAO	Age Group	% Males	Mean (C)YBOCS-T	Diagnostic Instrument	Any_Subs_Life	Any_Subs_Life %
1	deMathis 2008	Brazil	330	32.9	14.0	Adult	54.9	24.0	SCID-I	52	15.8
2	Shavitt 2014	Brazil	1001	34.9	21.8	Adult	43.2	25.5	SCID-I	67	6.7
3	Vorstenbosch 2012	Canada	180	32.9	18.0	Adult	37.2	NA	SCID	9	5.0
4	Ecker 2014	Germany	170	39.5	NA	Adult	NA	NA	Other	13	7.6
5	Lennertz 2013	Germany	232	36.7	20.1	Adult	47.4	NA	SADS & SCID	19	8.2
6	Garyfallos 2010	Greece	146	33.6	23.7	Adult	43.8	21.9	MINI	7	4.8
7	Viswanath 2012	India	545	29.3	21.8	Adult	60.9	23.3	MINI-Plus	17	3.1
8	Maina 2008	Italy	148	34.8	22.8	Adult	50.7	26.2	SCID-I and II	6	4.1
9	Albert 2019	Italy	251	37.4	22.2	Adult	53.8	24.1	SCID-I	20	8.0
10	Benatti 2020	Italy	229	41.1	22.4	Adult	50.7	24.2	SCID-I and II	29	12.7
11	Dell'Osso 2017	Italy	124	40.2	23.3	Adult	50.0	25.8	SCID-I and II	14	11.3
12	vanOudheusden 2020	Netherlands	419	36.6	18.5	Adult	44.2	19.9	SCID-I	53	12.6
13	Denys 2004	Netherlands	420	36.0	19.8	Adult	38.0	24.2	MINI	18	4.3
14	vanderVeld	Netherlands	400	35.1	NA	Adult	35.5	NA	MINI	[illegible]	[illegible]

14	vanderveld 2020	Netherlands	400	35.1	NA	Adult	35.5	NA	MINI	13	3.2
15	Klein 2020	United States	290	34.5	NA	Adult	52.8	NA	MINI	21	7.2
16	Mancebo 2009	United States	323	40.1	18.5	Adult	45.0	20.6	SCID-I and II	87	26.9
17	Leonard 2014	United States	126	15.5	NA	Pediatric	47.6	24.9	Other	1	0.8

—————————————————————————-

Suppl Table 24. Autism spectrum disorder

Total number of studies that reported Autism spectrum disorder = 7

Number of pediatric studies that reported Autism spectrum disorder = 6

Total N of OCD cases in studies that reported Autism spectrum disorder = 1497

Total cases of Autism spectrum disorder = 114

No	Study	Country	Total OCD Subjects	Mean Age	Mean AAO	Age Group	% Males	Mean (C)YBOCS-T	Diagnostic Instrument	ASD_Life	ASD_Life %
1	Jaspers-Fayer 2017	Canada	136	13.0	9.4	Pediatric	54.0	21.2	ADIS-IV	8	5.9
2	Adam 2019	Germany	181	13.2	NA	Pediatric	50.3	NA	Other	9	5.0
3	vanOudheusden 2020	Netherlands	419	36.6	18.5	Adult	44.2	19.9	SCID-I	27	6.4
4	Sevilla-Cermeno 2019	Sweden	193	13.6	10.8	Pediatric	44.6	22.6	MINI-KID	38	19.7
5	Cervin 2020	Sweden	120	13.3	8.4	Pediatric	37.5	23.2	MINI-KID	8	6.7
6	Peris 2017	United States	322	12.3	NA	Pediatric	53.0	25.2	ADIS-IV	10	3.1
7	Leonard 2014	United States	126	15.5	NA	Pediatric	47.6	24.9	Other	14	11.1

—————————————————————————-

Suppl Table 25. Attention deficit hyperactivity disorder

Total number of studies that reported Attention deficit hyperactivity disorder = 19

Number of pediatric studies that reported Attention deficit hyperactivity disorder = 13

Total N of OCD cases in studies that reported Attention deficit hyperactivity disorder = 4761

Total cases of Attention deficit hyperactivity disorder = 815

No	Study	Country	Total OCD Subjects	Mean Age	Mean AAO	Age Group	% Males	Mean (C)YBOCS-T	Diagnostic Instrument	ADHD_Life	ADHD_Life %
1	deMathis 2008	Brazil	330	32.9	14.0	Adult	54.9	24.0	SCID-I	46	13.9
2	Shavitt 2014	Brazil	1001	34.9	21.8	Adult	43.2	25.5	SCID-I	127	12.7
3	Jaspers-Fayer 2017	Canada	136	13.0	9.4	Pediatric	54.0	21.2	ADIS-IV	12	8.8
4	Selles 2020	Canada	243	13.6	11.7	Pediatric	42.4	NA	ADIS-IV	49	20.2
5	Nissen 2018	Denmark	317	13.9	8.9	Pediatric	36.0	24.5	KSADS-PL	42	13.2
6	Delorme 2005	France	161	23.9	12.6	Adult	55.3	NA	DIGS & KSADS-PL	45	28.0
7	Adam 2019	Germany	181	13.2	NA	Pediatric	50.3	NA	Other	14	7.7
8	vanOudheusden 2020	Netherlands	419	36.6	18.5	Adult	44.2	19.9	SCID-I	90	21.5
9	Ortiz 2016	Spain	112	14.7	12.1	Pediatric	50.9	17.5	KSADS-PL	11	9.8
10	Boloc 2019	Spain	101	14.6	11.9	Pediatric	49.5	18.8	KSADS-PL	12	11.9
11	Sevilla-Cermeno 2019	Sweden	193	13.6	10.8	Pediatric	44.6	22.6	MINI-KID	45	23.3
12	Cervin 2020	Sweden	120	13.3	8.4	Pediatric	37.5	23.2	MINI-KID	20	16.7
13	Geller 2000	United States	187	11.9	7.4	Pediatric	69.5	NA	K-SADS-PL	99	52.9
14	Geller 2001	United States	101	NA	8.2	Pediatric	65.3	23.0	K-SADS-E	43	42.6
15	Nota 2020	United States	173	29.0	13.2	Adult	NA	14.7	SCID-5	27	15.6
16	Mancebo 2009	United States	323	40.1	18.5	Adult	45.0	20.6	SCID-I and II	34	10.5
17	Rozenman 2019	United States	215	12.2	NA	Pediatric	57.0	24.9	ADIS-IV	26	12.1
18	Peris 2017	United States	322	12.3	NA	Pediatric	53.0	25.2	ADIS-IV	42	13.0
19	Leonard 2014	United States	126	15.5	NA	Pediatric	47.6	24.9	Other	31	24.6

—————————————————————————-

Suppl Table 26. Oppositional defiant disorder

Total number of studies that reported Oppositional defiant disorder = 7

Number of pediatric studies that reported Oppositional defiant disorder = 7

Total N of OCD cases in studies that reported Oppositional defiant disorder = 1095

Total cases of Oppositional defiant disorder = 174

No	Study	Country	Total OCD Subjects	Mean Age	Mean AAO	Age Group	% Males	Mean (C)YBOCS-T	Diagnostic Instrument	ODD_Life	ODD_Life %
1	Jaspers-Fayer 2017	Canada	136	13.0	9.4	Pediatric	54.0	21.2	ADIS-IV	5	3.7
2	Selles 2020	Canada	243	13.6	11.7	Pediatric	42.4	NA	ADIS-IV	11	4.5
3	Ortiz 2016	Spain	112	14.7	12.1	Pediatric	50.9	17.5	KSADS-PL	6	5.4
4	Boloc 2019	Spain	101	14.6	11.9	Pediatric	49.5	18.8	KSADS-PL	1	1.0
5	Geller 2000	United States	187	11.9	7.4	Pediatric	69.5	NA	K-SADS-PL	91	48.7
6	Geller 2001	United States	101	NA	8.2	Pediatric	65.3	23.0	K-SADS-E	49	48.5
7	Rozenman 2019	United States	215	12.2	NA	Pediatric	57.0	24.9	ADIS-IV	11	5.1

—————————————————————————-

Suppl Table 27. Any personality disorder

Total number of studies that reported Any personality disorder = 7

Number of pediatric studies that reported Any personality disorder = 0

Total N of OCD cases in studies that reported Any personality disorder = 1970

Total cases of Any personality disorder = 716

No	Study	Country	Total OCD Subjects	Mean Age	Mean AAO	Age Group	% Males	Mean (C)YBOCS-T	Diagnostic Instrument	Any_AxisII	Any_AxisII %
1	Melca 2015	Brazil	110	39.4	NA	Adult	50.0	NA	SCID-I	58	52.7
2	Ecker 2014	Germany	170	39.5	NA	Adult	NA	NA	Other	41	24.1
3	Albert 2015	Italy	483	34.4	22.1	Adult	52.0	25.0	SCID-I	217	44.9
4	Bulli 2016	Italy	159	34.6	NA	Adult	54.1	25.8	MINI	33	20.8
5	Denys 2004	Netherlands	420	36.0	19.8	Adult	38.0	24.2	MINI	151	36.0
6	Falkenstein 2019	United States	305	30.2	13.0	Adult	54.4	25.3	SCID-I and II	87	28.5
7	Mancebo 2009	United States	323	40.1	18.5	Adult	45.0	20.6	SCID-I and II	129	39.9

—————————————————————————-

Suppl Table 28. Obsessive compulsive (Anankastic) personality disorder

Total number of studies that reported Obsessive compulsive (Anankastic) personality disorder = 12

Number of pediatric studies that reported Obsessive compulsive (Anankastic) personality disorder = 0

Total N of OCD cases in studies that reported Obsessive compulsive (Anankastic) personality disorder = 2518

Total cases of Obsessive compulsive (Anankastic) personality disorder = 437

No	Study	Country	Total OCD Subjects	Mean Age	Mean AAO	Age Group	% Males	Mean (C)YBOCS-T	Diagnostic Instrument	ObsCompPD	ObsCompPD %
1	Melca 2015	Brazil	110	39.4	NA	Adult	50.0	NA	SCID-I	23	20.9
2	Garyfallos 2010	Greece	146	33.6	23.7	Adult	43.8	21.9	MINI	45	30.8
3	Prabhu 2013	India	161	28.9	20.0	Adult	55.9	28.4	MINI-Plus	25	15.5
4	Balachander 2020	India	420	29.5	19.3	Adult	59.8	29.9	MINI	31	7.4
5	Maina 2008	Italy	148	34.8	22.8	Adult	50.7	26.2	SCID-I and II	43	29.1
6	Benatti 2020	Italy	229	41.1	22.4	Adult	50.7	24.2	SCID-I and II	36	15.7
7	Bulli 2016	Italy	159	34.6	NA	Adult	54.1	25.8	MINI	15	9.4
8	Dell'Osso 2017	Italy	124	40.2	23.3	Adult	50.0	25.8	SCID-I and II	27	21.8
9	Matsunaga 2010	Japan	168	30.3	22.4	Adult	34.5	27.7	SCID-I and II	15	8.9
10	Denys 2004	Netherlands	420	36.0	19.8	Adult	38.0	24.2	MINI	38	9.0
11	Lochner 2004	South Africa	110	35.2	18.1	Adult	32.7	NA	SCID-I	55	50.0
12	Mancebo 2009	United States	323	40.1	18.5	Adult	45.0	20.6	SCID-I and II	84	26.0

—————————————————————————-

Suppl Table 29. Schizoid personality disorder

Total number of studies that reported Schizoid personality disorder = 8

Number of pediatric studies that reported Schizoid personality disorder = 0

Total N of OCD cases in studies that reported Schizoid personality disorder = 1936

Total cases of Schizoid personality disorder = 101

No	Study	Country	Total OCD Subjects	Mean Age	Mean AAO	Age Group	% Males	Mean (C)YBOCS-T	Diagnostic Instrument	SchizoidPD	SchizoidPD %
1	Garyfallos 2010	Greece	146	33.6	23.7	Adult	43.8	21.9	MINI	0	0.0
2	Prabhu 2013	India	161	28.9	20.0	Adult	55.9	28.4	MINI-Plus	1	0.6
3	Balachander 2020	India	420	29.5	19.3	Adult	59.8	29.9	MINI	1	0.2
4	Maina 2008	Italy	148	34.8	22.8	Adult	50.7	26.2	SCID-I and II	12	8.1
5	Bulli 2016	Italy	159	34.6	NA	Adult	54.1	25.8	MINI	0	0.0
6	Denys 2004	Netherlands	420	36.0	19.8	Adult	38.0	24.2	MINI	0	0.0
7	Gordon 2013	UK	189	37.4	16.6	Adult	46.6	NA	SCID-I and II	85	45.0
8	Pinto 2006	United States	293	40.5	18.5	Adult	45.0	20.3	SCID-I and II	2	0.7

—————————————————————————-

Suppl Table 30. Schizotypal personality disorder

Total number of studies that reported Schizotypal personality disorder = 11

Number of pediatric studies that reported Schizotypal personality disorder = 0

Total N of OCD cases in studies that reported Schizotypal personality disorder = 2324

Total cases of Schizotypal personality disorder = 87

No	Study	Country	Total OCD Subjects	Mean Age	Mean AAO	Age Group	% Males	Mean (C)YBOCS-T	Diagnostic Instrument	SchizotypalPD	SchizotypalPD %
1	Melca 2015	Brazil	110	39.4	NA	Adult	50.0	NA	SCID-I	15	13.6
2	Garyfallos 2010	Greece	146	33.6	23.7	Adult	43.8	21.9	MINI	8	5.5
3	Prabhu 2013	India	161	28.9	20.0	Adult	55.9	28.4	MINI-Plus	2	1.2
4	Balachander 2020	India	420	29.5	19.3	Adult	59.8	29.9	MINI	16	3.8
5	Maina 2008	Italy	148	34.8	22.8	Adult	50.7	26.2	SCID-I and II	15	10.1
6	Bulli 2016	Italy	159	34.6	NA	Adult	54.1	25.8	MINI	1	0.6
7	Matsunaga 2010	Japan	168	30.3	22.4	Adult	34.5	27.7	SCID-I and II	9	5.4
8	Denys 2004	Netherlands	420	36.0	19.8	Adult	38.0	24.2	MINI	5	1.2
9	Lochner 2004	South Africa	110	35.2	18.1	Adult	32.7	NA	SCID-I	3	2.7
10	Gordon 2013	UK	189	37.4	16.6	Adult	46.6	NA	SCID-I and II	10	5.3
11	Pinto 2006	United States	293	40.5	18.5	Adult	45.0	20.3	SCID-I and II	3	1.0

—————————————————————————-

Suppl Table 31. Borderline personality disorder

Total number of studies that reported Borderline personality disorder = 9

Number of pediatric studies that reported Borderline personality disorder = 0

Total N of OCD cases in studies that reported Borderline personality disorder = 1997

Total cases of Borderline personality disorder = 168

No	Study	Country	Total OCD Subjects	Mean Age	Mean AAO	Age Group	% Males	Mean (C)YBOCS-T	Diagnostic Instrument	BorderlinePD	BorderlinePD %
1	Melca 2015	Brazil	110	39.4	NA	Adult	50.0	NA	SCID-I	24	21.8
2	Garyfallos 2010	Greece	146	33.6	23.7	Adult	43.8	21.9	MINI	21	14.4
3	Prabhu 2013	India	161	28.9	20.0	Adult	55.9	28.4	MINI-Plus	3	1.9
4	Balachander 2020	India	420	29.5	19.3	Adult	59.8	29.9	MINI	36	8.6
5	Maina 2008	Italy	148	34.8	22.8	Adult	50.7	26.2	SCID-I and II	20	13.5
6	Bulli 2016	Italy	159	34.6	NA	Adult	54.1	25.8	MINI	3	1.9
7	Denys 2004	Netherlands	420	36.0	19.8	Adult	38.0	24.2	MINI	24	5.7
8	Lochner 2004	South Africa	110	35.2	18.1	Adult	32.7	NA	SCID-I	16	14.5
9	Mancebo 2009	United States	323	40.1	18.5	Adult	45.0	20.6	SCID-I and II	21	6.5

—————————————————————————-

Suppl Table 32. Histrionic personality disorder

Total number of studies that reported Histrionic personality disorder = 7

Number of pediatric studies that reported Histrionic personality disorder = 0

Total N of OCD cases in studies that reported Histrionic personality disorder = 1775

Total cases of Histrionic personality disorder = 63

No	Study	Country	Total OCD Subjects	Mean Age	Mean AAO	Age Group	% Males	Mean (C)YBOCS-T	Diagnostic Instrument	HistrionicPD	HistrionicPD %
1	Garyfallos 2010	Greece	146	33.6	23.7	Adult	43.8	21.9	MINI	9	6.2
2	Balachander 2020	India	420	29.5	19.3	Adult	59.8	29.9	MINI	2	0.5
3	Maina 2008	Italy	148	34.8	22.8	Adult	50.7	26.2	SCID-I and II	9	6.1
4	Bulli 2016	Italy	159	34.6	NA	Adult	54.1	25.8	MINI	1	0.6
5	Denys 2004	Netherlands	420	36.0	19.8	Adult	38.0	24.2	MINI	6	1.4
6	Gordon 2013	UK	189	37.4	16.6	Adult	46.6	NA	SCID-I and II	34	18.0
7	Pinto 2006	United States	293	40.5	18.5	Adult	45.0	20.3	SCID-I and II	2	0.7

—————————————————————————-

Suppl Table 33. Antisocial personality disorder

Total number of studies that reported Antisocial personality disorder = 8

Number of pediatric studies that reported Antisocial personality disorder = 0

Total N of OCD cases in studies that reported Antisocial personality disorder = 1885

Total cases of Antisocial personality disorder = 22

No	Study	Country	Total OCD Subjects	Mean Age	Mean AAO	Age Group	% Males	Mean (C)YBOCS-T	Diagnostic Instrument	AntisocialPD	AntisocialPD %
1	Melca 2015	Brazil	110	39.4	NA	Adult	50.0	NA	SCID-I	3	2.7
2	Garyfallos 2010	Greece	146	33.6	23.7	Adult	43.8	21.9	MINI	1	0.7
3	Balachander 2020	India	420	29.5	19.3	Adult	59.8	29.9	MINI	2	0.5
4	Maina 2008	Italy	148	34.8	22.8	Adult	50.7	26.2	SCID-I and II	9	6.1
5	Bulli 2016	Italy	159	34.6	NA	Adult	54.1	25.8	MINI	0	0.0
6	Denys 2004	Netherlands	420	36.0	19.8	Adult	38.0	24.2	MINI	2	0.5
7	Gordon 2013	UK	189	37.4	16.6	Adult	46.6	NA	SCID-I and II	2	1.1
8	Pinto 2006	United States	293	40.5	18.5	Adult	45.0	20.3	SCID-I and II	3	1.0

—————————————————————————-

Suppl Table 34. Narcissistic personality disorder

Total number of studies that reported Narcissistic personality disorder = 7

Number of pediatric studies that reported Narcissistic personality disorder = 0

Total N of OCD cases in studies that reported Narcissistic personality disorder = 1775

Total cases of Narcissistic personality disorder = 42

No	Study	Country	Total OCD Subjects	Mean Age	Mean AAO	Age Group	% Males	Mean (C)YBOCS-T	Diagnostic Instrument	NarcicissticPD	NarcicissticPD %
1	Garyfallos 2010	Greece	146	33.6	23.7	Adult	43.8	21.9	MINI	3	2.1
2	Balachander 2020	India	420	29.5	19.3	Adult	59.8	29.9	MINI	1	0.2
3	Maina 2008	Italy	148	34.8	22.8	Adult	50.7	26.2	SCID-I and II	12	8.1
4	Bulli 2016	Italy	159	34.6	NA	Adult	54.1	25.8	MINI	10	6.3
5	Denys 2004	Netherlands	420	36.0	19.8	Adult	38.0	24.2	MINI	10	2.4
6	Gordon 2013	UK	189	37.4	16.6	Adult	46.6	NA	SCID-I and II	6	3.2
7	Pinto 2006	United States	293	40.5	18.5	Adult	45.0	20.3	SCID-I and II	0	0.0

—————————————————————————-

Suppl Table 35. Dependent personality disorder

Total number of studies that reported Dependent personality disorder = 8

Number of pediatric studies that reported Dependent personality disorder = 0

Total N of OCD cases in studies that reported Dependent personality disorder = 1936

Total cases of Dependent personality disorder = 99

No	Study	Country	Total OCD Subjects	Mean Age	Mean AAO	Age Group	% Males	Mean (C)YBOCS-T	Diagnostic Instrument	DependentPD	DependentPD %
1	Garyfallos 2010	Greece	146	33.6	23.7	Adult	43.8	21.9	MINI	15	10.3
2	Prabhu 2013	India	161	28.9	20.0	Adult	55.9	28.4	MINI-Plus	1	0.6
3	Balachander 2020	India	420	29.5	19.3	Adult	59.8	29.9	MINI	6	1.4
4	Maina 2008	Italy	148	34.8	22.8	Adult	50.7	26.2	SCID-I and II	25	16.9
5	Bulli 2016	Italy	159	34.6	NA	Adult	54.1	25.8	MINI	0	0.0
6	Denys 2004	Netherlands	420	36.0	19.8	Adult	38.0	24.2	MINI	32	7.6
7	Gordon 2013	UK	189	37.4	16.6	Adult	46.6	NA	SCID-I and II	17	9.0
8	Pinto 2006	United States	293	40.5	18.5	Adult	45.0	20.3	SCID-I and II	3	1.0

—————————————————————————-

Suppl Table 36. Anxious-avoidant personality disorder

Total number of studies that reported Anxious-avoidant personality disorder = 9

Number of pediatric studies that reported Anxious-avoidant personality disorder = 0

Total N of OCD cases in studies that reported Anxious-avoidant personality disorder = 2076

Total cases of Anxious-avoidant personality disorder = 187

No	Study	Country	Total OCD Subjects	Mean Age	Mean AAO	Age Group	% Males	Mean (C)YBOCS-T	Diagnostic Instrument	AnxAvoidPD	AnxAvoidPD %
1	Garyfallos 2010	Greece	146	33.6	23.7	Adult	43.8	21.9	MINI	26	17.8
2	Prabhu 2013	India	161	28.9	20.0	Adult	55.9	28.4	MINI-Plus	15	9.3
3	Balachander 2020	India	420	29.5	19.3	Adult	59.8	29.9	MINI	17	4.0
4	Maina 2008	Italy	148	34.8	22.8	Adult	50.7	26.2	SCID-I and II	31	20.9
5	Bulli 2016	Italy	159	34.6	NA	Adult	54.1	25.8	MINI	3	1.9
6	Denys 2004	Netherlands	420	36.0	19.8	Adult	38.0	24.2	MINI	19	4.5
7	Lochner 2004	South Africa	110	35.2	18.1	Adult	32.7	NA	SCID-I	9	8.2
8	Gordon 2013	UK	189	37.4	16.6	Adult	46.6	NA	SCID-I and II	18	9.5
9	Mancebo 2009	United States	323	40.1	18.5	Adult	45.0	20.6	SCID-I and II	49	15.2

—————————————————————————-

Complete references for the 91 studies included in the meta-analysis

1. Adam, J., Goletz, H., Mattausch, S.-K., Plück, J., Döpfner, M., 2019. Psychometric evaluation of a parent-rating and self-rating inventory for pediatric obsessive-compulsive disorder: German OCD Inventory for Children and Adolescents (OCD-CA). Child and Adolescent Psychiatry and Mental Health 13.
2. Albert, U., Aguglia, A., Chiarle, A., Bogetto, F., Maina, G., 2013. Metabolic syndrome and obsessive-compulsive disorder: A naturalistic Italian study. General Hospital Psychiatry 35, 154–159. https://doi.org/10.1016/j.genhosppsych.2012.10.004
3. Albert, U., Barbaro, F., Bramante, S., Rosso, G., De Ronchi, D., Maina, G., 2019. Duration of untreated illness and response to SRI treatment in Obsessive-Compulsive Disorder. European Psychiatry 58, 19–26. https://doi.org/10.1016/j.eurpsy.2019.01.017
4. Albert, U., Manchia, M., Tortorella, A., Volpe, U., Rosso, G., Carpiniello, B., Maina, G., 2015. Admixture analysis of age at symptom onset and age at disorder onset in a large sample of patients with obsessive-compulsive disorder. Journal of Affective Disorders 187, 188–196. https://doi.org/10.1016/j.jad.2015.07.045
5. Arumugham, S.S., Cherian, A.V., Baruah, U., Viswanath, B., Narayanaswamy, J.C., Math, S.B., Reddy, Y.C.J., 2014. Comparison of clinical characteristics of familial and sporadic obsessive-compulsive disorder. Comprehensive Psychiatry 55, 1520–1525. https://doi.org/10.1016/j.comppsych.2014.07.006
6. Balachander, S., Bajaj, A., Hazari, N., Kumar, A., Anand, N., Manjula, M., Sudhir, P.M., Cherian, A.V., Narayanaswamy, J.C., Jaisoorya, T.S., Bada Math, S., Kandavel, T., Arumugham, S.S., Reddy, Y.C.J., 2020. Long-term Outcomes of Intensive Inpatient Care for Severe, Resistant Obsessive-Compulsive Disorder. Canadian Journal of Psychiatry. https://doi.org/10.1177/0706743720927830
7. Benatti, B., Celebre, L., Girone, N., Priori, A., Bruno, A., Viganò, C., Hollander, E., Dell'Osso, B., 2020. Clinical characteristics and comorbidity associated with female gender in obsessive-compulsive disorder. Journal of Psychiatric Research 131, 209–214. https://doi.org/10.1016/j.jpsychires.2020.09.019
8. Bhattacharyya, S., Janardhan Reddy, Y.C., Khanna, S., 2005. Depressive and anxiety disorder comorbidity in obsessive compulsive disorder. Psychopathology 38, 315–319. https://doi.org/10.1159/000088920
9. Bo, Y.L., Liu, S., Yin, Y., Wang, Z., Cui, J., Zong, J., Zhang, X., Li, X., 2013. Association study between IL-1ß-511 C/T polymorphism and obsessive-compulsive disorder (OCD) in Chinese Han population. International Journal of Psychiatry in Medicine 46, 145–152. https://doi.org/10.2190/PM.46.2.b
10. Boloc, D., Mas, S., Rodriguez, N., Ortiz, A.E., Morer, A., Plana, M.T., Lafuente, A., Lazaro, L., Gassó, P., 2019. Genetic associations of serotoninergic and gabaergic genes in an extended collection of early-onset obsessive-compulsive disorder trios. Journal of Child and Adolescent Psychopharmacology 29, 152–157. https://doi.org/10.1089/cap.2018.0073
11. Brakoulias, V., Starcevic, V., Berle, D., Milicevic, D., Hannan, A., Martin, A., 2014. The relationships between obsessive-compulsive symptom dimensions and cognitions in obsessive-compulsive disorder. Psychiatric Quarterly 85, 133–142. https://doi.org/10.1007/s11126-013-9278-y
12. Bulli, F., Melli, G., Cavalletti, V., Stopani, E., Carraresi, C., 2016. Comorbid Personality Disorders in Obsessive–Compulsive Disorder and Its Symptom Dimensions. Psychiatric Quarterly 87, 365–376. https://doi.org/10.1007/s11126-015-9393-z
13. Calamari, J.E., Rector, N.A., Woodard, J.L., Cohen, R.J., Chik, H.M., 2008. Anxiety sensitivity and obsessive-compulsive disorder. Assessment 15, 351–363. https://doi.org/10.1177/1073191107312611
14. Camarena, B., Loyzaga, C., Aguilar, A., Weissbecker, K., Nicolini, H., 2007. Association study between the dopamine receptor D4 gene and obsessive-compulsive disorder. European Neuropsychopharmacology 17, 406–409. https://doi.org/10.1016/j.euroneuro.2006.08.001
15. Camarena, B., Rinetti, G., Cruz, C., Gomez, A., De La Fuente, J.R., Nicolini, H., 2001. Additional evidence that genetic variation of MAO-A gene supports a gender subtype in

obsessive-compulsive disorder. American Journal of Medical Genetics - Neuropsychiatric Genetics 105, 279–282. https://doi.org/10.1002/ajmg.1323
16. Cameron, D.H., Streiner, D.L., Summerfeldt, L.J., Rowa, K., McKinnon, M.C., McCabe, R.E., 2019. A comparison of cluster and factor analytic techniques for identifying symptom-based dimensions of obsessive-compulsive disorder. Psychiatry Research 278, 86–96. https://doi.org/10.1016/j.psychres.2019.05.040
17. Castle, D.J., Deale, A., Marks, I.M., 1995. Gender differences in obsessive compulsive disorder. Australian and New Zealand Journal of Psychiatry 29, 114–117. https://doi.org/10.3109/00048679509075899
18. Cervin, M., Perrin, S., Olsson, E., Claesdotter-Knutsson, E., Lindvall, M., 2020. Involvement of fear, incompleteness, and disgust during symptoms of pediatric obsessive–compulsive disorder. European Child and Adolescent Psychiatry. https://doi.org/10.1007/s00787-020-01514-7
19. De Haan, L., Dudek-Hodge, C., Verhoeven, Y., Denys, D., 2009. Prevalence of psychotic disorders in patients with obsessive-compulsive disorder. CNS Spectrums 14, 415–417.
20. de Mathis, M.A., do Rosario, M.C., Diniz, J.B., Torres, A.R., Shavitt, R.G., Ferrão, Y.A., Fossaluza, V., de Bragança Pereira, C.A., Miguel, E.C., 2008. Obsessive-compulsive disorder: Influence of age at onset on comorbidity patterns. European Psychiatry 23, 187–194. https://doi.org/10.1016/j.eurpsy.2008.01.002
21. Deacon, B.J., Abramowitz, J.S., 2005. The Yale-Brown Obsessive Compulsive Scale: Factor analysis, construct validity, and suggestions for refinement. Journal of Anxiety Disorders 19, 573–585. https://doi.org/10.1016/j.janxdis.2004.04.009
22. Dell'Osso, B., Benatti, B., Hollander, E., Altamura, A.C., 2017. Clinical features associated with increased severity of illness in tertiary clinic referred patients with obsessive compulsive disorder. International Journal of Psychiatry in Clinical Practice 21, 131–136. https://doi.org/10.1080/13651501.2016.1249891
23. Dell'Osso, B., Benatti, B., Hollander, E., Fineberg, N., Stein, D.J., Lochner, C., Nicolini, H., Lanzagorta, N., Palazzo, C., Altamura, A.C., Marazziti, D., Pallanti, S., Van Ameringen, M., Karamustafalioglu, O., Drummond, L.M., Hranov, L., Figee, M., Grant, J.E., Zohar, J., Denys, D., Menchon, J.M., 2016. Childhood, adolescent and adult age at onset and related clinical correlates in obsessive–compulsive disorder: a report from the International College of Obsessive–Compulsive Spectrum Disorders (ICOCS). International Journal of Psychiatry in Clinical Practice 20, 210–217. https://doi.org/10.1080/13651501.2016.1207087
24. Dell'Osso, B., Nicolini, H., Lanzagorta, N., Benatti, B., Spagnolin, G., Palazzo, M.C., Marazziti, D., Hollander, E., Fineberg, N., Stein, D.J., Pallanti, S., Van Ameringen, M., Lochner, C., Hranov, G., Karamustafalioglu, O., Hranov, L., Zohar, J., Denys, D., Altamura, A.C., Menchon, J.M., 2015. Cigarette smoking in patients with obsessive compulsive disorder: A report from the International College of Obsessive Compulsive Spectrum Disorders (ICOCS). CNS Spectrums 20, 469–473. https://doi.org/10.1017/S1092852915000565
25. Dell'osso, B., Vismara, M., Benatti, B., Cirnigliaro, G., Grancini, B., Fineberg, N.A., Van Ameringen, M., Hollander, E., Stein, D.J., Menchon, J.M., Rodriguez, C.I., Nicolini, H., Lanzagorta, N., Pallanti, S., Grassi, G., Lochner, C., Marazziti, D., Hranov, G., Karamustafalioglu, O., Hranov, L., Zohar, J., 2020. Lifetime bipolar disorder comorbidity and related clinical characteristics in patients with primary obsessive compulsive disorder: A report from the International College of Obsessive-Compulsive Spectrum Disorders (ICOCS). CNS Spectrums 25, 419–425. https://doi.org/10.1017/S1092852919001068
26. Delorme, R., Golmard, J.-L., Chabane, N., Millet, B., Krebs, M.-O., Mouren-Simeoni, M.C., Leboyer, M., 2005. Admixture analysis of age at onset in obsessive-compulsive disorder. Psychological Medicine 35, 237–243. https://doi.org/10.1017/S0033291704003253
27. Denys, D., Tenney, N., Van Megen, H.J.G.M., De Geus, F., Westenberg, H.G.M., 2004. Axis I and II comorbidity in a large sample of patients with obsessive-compulsive disorder. Journal of Affective Disorders 80, 155–162. https://doi.org/10.1016/S0165-0327(03)00056-9

28. Denys, D., Van Nieuwerburgh, F., Deforce, D., Westenberg, H., 2006. Association between the dopamine D2 receptor TaqI A2 allele and low activity COMT allele with obsessive-compulsive disorder in males. European Neuropsychopharmacology 16, 446–450. https://doi.org/10.1016/j.euroneuro.2005.12.001
29. dos Santos-Ribeiro, S., Lins-Martins, N.M., Frydman, I., Conceição do Rosário, M., Ferrão, Y.A., Shavitt, R.G., Yücel, M., Miguel, E.C., Fontenelle, L.F., 2016. Prevalence and correlates of electroconvulsive therapy delivery in 1001 obsessive-compulsive disorder outpatients. Psychiatry Research 239, 145–148. https://doi.org/10.1016/j.psychres.2016.03.020
30. Ecker, W., Kupfer, J., Gönner, S., 2014. Incompleteness and harm avoidance in OCD, anxiety and depressive disorders, and non-clinical controls. Journal of Obsessive-Compulsive and Related Disorders 3, 46–51. https://doi.org/10.1016/j.jocrd.2013.12.001
31. Eilertsen, T., Hansen, B., Kvale, G., Abramowitz, J.S., Holm, S.E.H., Solem, S., 2017. The dimensional obsessive-compulsive scale: Development and validation of a short form (DOCS-SF). Front. Psychol. 8, 8. https://doi.org/10.3389/fpsyg.2017.01503
32. Falkenstein, M.J., Nota, J.A., Krompinger, J.W., Schreck, M., Garner, L.E., Potluri, S., Van Kirk, N., Ponzini, G., Tifft, E., Brennan, B.P., Mathes, B., Cattie, J., Crosby, J.M., Elias, J.A., 2019. Empirically-derived response trajectories of intensive residential treatment in obsessive-compulsive disorder: A growth mixture modeling approach. Journal of Affective Disorders 245, 827–833. https://doi.org/10.1016/j.jad.2018.11.075
33. Fals-Stewart, W., Lucente, S., 1993. An MCMI cluster typology of obsessive-compulsives: A measure of personality characteristics and its relationship to treatment participation, compliance and outcome in behavior therapy. J. Psychiatr. Res. 27, 139–154. https://doi.org/10.1016/0022-3956(93)90002-J
34. Garner, L.E., Van Kirk, N., Tifft, E.D., Krompinger, J.W., Mathes, B.M., Fraire, M., Falkenstein, M.J., Brennan, B.P., Crosby, J.M., Elias, J.A., 2018. Validation of the distress tolerance scale-short form in obsessive compulsive disorder. Journal of clinical psychology 74, 916–925. https://doi.org/10.1002/jclp.22554
35. Garyfallos, G., Katsigiannopoulos, K., Adamopoulou, A., Papazisis, G., Karastergiou, A., Bozikas, V.P., 2010. Comorbidity of obsessive-compulsive disorder with obsessive-compulsive personality disorder: Does it imply a specific subtype of obsessive-compulsive disorder? Psychiatry Research 177, 156–160. https://doi.org/10.1016/j.psychres.2010.01.006
36. Geller, D., Biederman, J., Faraone, S.V., Frazier, J., Coffey, B.J., Kim, G., Bellordre, C.A., 2000. Clinical correlates of obsessive compulsive disorder in children and adolescents referred to specialized and non-specialized clinical settings. Depression and Anxiety 11, 163–168. https://doi.org/10.1002/1520-6394(2000)11:4<163::AID-DA3>3.0.CO;2-3
37. Geller, D.A., Biederman, J., Faraone, S., Agranat, A., Cradock, K., Hagermoser, L., Kim, G., Frazier, J., Coffey, B.J., 2001. Developmental aspects of obsessive compulsive disorder: Findings in children, adolescents, and adults. Journal of Nervous and Mental Disease 189, 471–477. https://doi.org/10.1097/00005053-200107000-00009
38. Gordon, O.M., Salkovskis, P.M., Oldfield, V.B., Carter, N., 2013. The association between obsessive compulsive disorder and obsessive compulsive personality disorder: prevalence and clinical presentation. The British journal of clinical psychology / the British Psychological Society 52, 300–315.
39. Grant, J.E., Mancebo, M.C., Pinto, A., Eisen, J.L., Rasmussen, S.A., 2006. Impulse control disorders in adults with obsessive compulsive disorder. Journal of Psychiatric Research 40, 494–501. https://doi.org/10.1016/j.jpsychires.2005.11.005
40. Hofmeijer-Sevink, M.K., Van Oppen, P., Van Megen, H.J., Batelaan, N.M., Cath, D.C., Van Der Wee, N.J.A., Van Den Hout, M.A., Van Balkom, A.J., 2013. Clinical relevance of comorbidity in obsessive compulsive disorder: The Netherlands OCD Association study. Journal of Affective Disorders 150, 847–854. https://doi.org/10.1016/j.jad.2013.03.014
41. Holm, S.E.H., Hansen, B., Kvale, G., Eilertsen, T., Hagen, K., Solem, S., 2019. Is sensory processing sensitivity related to treatment outcome in concentrated exposure and response prevention treatment for obsessive-compulsive disorder? Journal of

Obsessive-Compulsive and Related Disorders 23. https://doi.org/10.1016/j.jocrd.2019.100486
42. Jaspers-Fayer, F., Han, S.H.J., Chan, E., McKenney, K., Simpson, A., Boyle, A., Ellwyn, R., Stewart, S.E., 2017. Prevalence of Acute-Onset Subtypes in Pediatric Obsessive-Compulsive Disorder. Journal of Child and Adolescent Psychopharmacology 27, 332–341. https://doi.org/10.1089/cap.2016.0031
43. Karayiorgou, M., Sobin, C., Blundell, M.L., Galke, B.L., Malinova, L., Goldberg, P., Ott, J., Gogos, J.A., 1999. Family-based association studies support a sexually dimorphic effect of COMT and MAOA on genetic susceptibility to obsessive-compulsive disorder. Biological Psychiatry 45, 1178–1189. https://doi.org/10.1016/S0006-3223(98)00319-9
44. Katerberg, H., Cath, D.C., Denys, D.A.J.P., Heutink, P., Polman, A., Van Nieuwerburgh, F.C.W., Deforce, D.L.D., Bochdanovits, Z., Van Balkom, A.J.L.M., Den Boer, J.A., 2010. The role of the COMT Val158 met polymorphism in the phenotypic expression of obsessive-compulsive disorder. Am. J. Med. Genet. B Neuropsychiatr. Genet. 153, 167–176. https://doi.org/10.1002/ajmg.b.30971
45. Klein, K.P., Harris, E.K., Björgvinsson, T., Kertz, S.J., 2020. A network analysis of symptoms of obsessive compulsive disorder and depression in a clinical sample. Journal of Obsessive-Compulsive and Related Disorders 27. https://doi.org/10.1016/j.jocrd.2020.100556
46. Koh, M.J., Kim, W., Kang, J.I., Namkoong, K., Kim, S.J., 2015. Lack of association between oxytocin receptor (OXTR) gene polymorphisms and alexithymia: Evidence from patients with obsessive-compulsive disorder. PLoS ONE 10. https://doi.org/10.1371/journal.pone.0143168
47. Kugler, B.B., Lewin, A.B., Phares, V., Geffken, G.R., Murphy, T.K., Storch, E.A., 2013. Quality of life in obsessive-compulsive disorder: The role of mediating variables. Psychiatry Research 206, 43–49. https://doi.org/10.1016/j.psychres.2012.10.006
48. Kwee, C.M.B., van den Hout, M.A., 2019. Anxiety sensitivity does not predict treatment outcome or treatment length in obsessive-compulsive disorder and related anxiety disorders. Journal of Obsessive-Compulsive and Related Disorders 21, 18–25. https://doi.org/10.1016/j.jocrd.2018.11.003
49. Langley, A.K., Lewin, A.B., Bergman, R.L., Lee, J.C., Piacentini, J., 2010. Correlates of comorbid anxiety and externalizing disorders in childhood obsessive compulsive disorder. European Child and Adolescent Psychiatry 19, 637–645. https://doi.org/10.1007/s00787-010-0101-0
50. Leckman, J.F., Grice, D.E., Barr, L.C., de Vries, A.L., Martin, C., Cohen, D.J., McDougle, C.J., Goodman, W.K., Rasmussen, S.A., 1994. Tic-related vs. non-tic-related obsessive compulsive disorder. Anxiety 1, 208–215.
51. Lennertz, L., Franke, P.E., Grabe, H.J., Rampacher, F., Schulze-Rauschenbach, S., Guttenthaler, V., Ruhrmann, S., Pukrop, R., Klosterkötter, J., Falkai, P., Maier, W., Wagner, M., Mössner, R., 2013. The functional coding variant Asn107Ile of the neuropeptide S receptor gene (NPSR1) influences age at onset of obsessive-compulsive disorder. Int. J. Neuropsychopharmacol. 16, 1951–1958. https://doi.org/10.1017/S1461145713000382
52. Leonard, R.C., Jacobi, D.M., Riemann, B.C., Lake, P.M., Luhn, R., 2014. The effect of depression symptom severity on OCD treatment outcome in an adolescent residential sample. Journal of Obsessive-Compulsive and Related Disorders 3, 95–101. https://doi.org/10.1016/j.jocrd.2014.02.003
53. Lochner, C., Hemmings, S.M.J., Kinnear, C.J., Moolman-Smook, J.C., Corfield, V.A., Knowles, J.A., Niehaus, D.J.H., Stein, D.J., 2004a. Gender in obsessive-compulsive disorder: Clinical and genetic findings. European Neuropsychopharmacology 14, 105–113. https://doi.org/10.1016/S0924-977X(03)00063-4
54. Lochner, C., Seedat, S., Hemmings, S.M.J., Kinnear, C.J., Corfield, V.A., Niehaus, D.J.H., Moolman-Smook, J.C., Stein, D.J., 2004b. Dissociative experiences in obsessive-compulsive disorder and trichotillomania: Clinical and genetic findings. Comprehensive Psychiatry 45, 384–391. https://doi.org/10.1016/j.comppsych.2004.03.010
55. Maina, G., Albert, U., Salvi, V., Pessina, E., Bogetto, F., 2008. Early-onset obsessive-compulsive disorder and personality disorders in adulthood. Psychiatry Research 158, 217–225. https://doi.org/10.1016/j.psychres.2006.08.003

56. Mancebo, M.C., Grant, J.E., Pinto, A., Eisen, J.L., Rasmussen, S.A., 2009. Substance use disorders in an obsessive compulsive disorder clinical sample. Journal of Anxiety Disorders 23, 429–435. https://doi.org/10.1016/j.janxdis.2008.08.008
57. Mancebo, M.C., Greenberg, B., Grant, J.E., Pinto, A., Eisen, J.L., Dyck, I., Rasmussen, S.A., 2008. Correlates of occupational disability in a clinical sample of obsessive-compulsive disorder. Comprehensive Psychiatry 49, 43–50. https://doi.org/10.1016/j.comppsych.2007.05.016
58. Matsunaga, H., 2008. Symptom structure in Japanese patients with obsessive-compulsive disorder (American Journal of Psychiatry (2007) DOI: 10.1176/appi.ajp.2007.07020340). American Journal of Psychiatry 165, 266.
59. Matsunaga, H., Hayashida, K., Kiriike, N., Nagata, T., Stein, D.J., 2010. Clinical features and treatment characteristics of compulsive hoarding in japanese patients with obsessive-compulsive disorder. CNS Spectrums 15, 258–265. https://doi.org/10.1017/s1092852900000092
60. Melca, I.A., Yücel, M., Mendlowicz, M.V., de Oliveira-Souza, R., Fontenelle, L.F., 2015. The correlates of obsessive-compulsive, Schizotypal, And borderline personality disorders in obsessive-compulsive disorder. Journal of Anxiety Disorders 33, 15–24. https://doi.org/10.1016/j.janxdis.2015.04.004
61. Merrill, A., Gershuny, B., Baer, L., Jenike, M.A., 2011. Depression in comorbid obsessive-compulsive disorder and posttraumatic stress disorder. J. Clin. Psychol. 67, 624–628. https://doi.org/10.1002/jclp.20783
62. Milanfranchi, A., Marazziti, D., Pfanner, C., Presta, S., Lensi, P., Ravagli, S., Cassano, G.B., 1995. Comorbidity in obsessive-compulsive disorder: Focus on depression. European Psychiatry 10, 379–382. https://doi.org/10.1016/0924-9338(96)80341-5
63. Nissen, J.B., Parner, E., 2018. The importance of insight, avoidance behavior, not-just-right perception and personality traits in pediatric obsessive-compulsive disorder (OCD): a naturalistic clinical study. Nordic Journal of Psychiatry 72, 489–496. https://doi.org/10.1080/08039488.2018.1486454
64. Nota, J.A., Potluri, S., Kelley, K.N., Elias, J.A., Krompinger, J.W., 2020. Delayed Bedtimes Are Associated With More Severe Obsessive-Compulsive Symptoms in Intensive Residential Treatment. Behavior Therapy 51, 559–571. https://doi.org/10.1016/j.beth.2019.12.006
65. Ojserkis, R., Boisseau, C.L., Reddy, M.K., Mancebo, M.C., Eisen, J.L., Rasmussen, S.A., 2017. The impact of lifetime PTSD on the seven-year course and clinical characteristics of OCD. Psychiatry Research 258, 78–82. https://doi.org/10.1016/j.psychres.2017.09.042
66. Ortiz, A.E., Morer, A., Moreno, E., Plana, M.T., Cordovilla, C., Lázaro, L., 2016. Clinical significance of psychiatric comorbidity in children and adolescents with obsessive–compulsive disorder: subtyping a complex disorder. European Archives of Psychiatry and Clinical Neuroscience 266, 199–208. https://doi.org/10.1007/s00406-015-0642-9
67. Pardue, C.M., Sibrava, N.J., Boisseau, C.L., Mancebo, M.C., Eisen, J.L., Rasmussen, S.A., 2014. Differential parental influence in the familial aggregation of obsessive compulsive disorder. Journal of Obsessive-Compulsive and Related Disorders 3, 215–219. https://doi.org/10.1016/j.jocrd.2014.05.001
68. Peris, T.S., Rozenman, M., Bergman, R.L., Chang, S., O’Neill, J., Piacentini, J., 2017. Developmental and clinical predictors of comorbidity for youth with obsessive compulsive disorder. Journal of Psychiatric Research 93, 72–78. https://doi.org/10.1016/j.jpsychires.2017.05.002
69. Piacentini, J., Bergman, R.L., Keller, M., McCracken, J., 2003. Functional impairment in children and adolescents with obsessive-compulsive disorder. Journal of Child and Adolescent Psychopharmacology 13, S61–S69.
70. Pinto, A., Mancebo, M.C., Eisen, J.L., Pagano, M.E., Rasmussen, S.A., 2006. The Brown Longitudinal Obsessive Compulsive Study: Clinical features and symptoms of the sample at intake. J. Clin. Psychiatry 67, 703–711. https://doi.org/10.4088/JCP.v67n0503
71. Prabhu, L., Cherian, A.V., Viswanath, B., Kandavel, T., Bada Math, S., Janardhan Reddy, Y.C., 2013. Symptom dimensions in OCD and their association with clinical characteristics and comorbid disorders. Journal of Obsessive-Compulsive and Related Disorders 2, 14–21. https://doi.org/10.1016/j.jocrd.2012.10.002

72. Reid, A.M., Garner, L.E., Van Kirk, N., Gironda, C., Krompinger, J.W., Brennan, B.P., Mathes, B.M., Monaghan, S.C., Tifft, E.D., André, M.-C., Cattie, J., Crosby, J.M., Elias, J.A., 2017. How willing are you? Willingness as a predictor of change during treatment of adults with obsessive–compulsive disorder. Depression and Anxiety 34, 1057–1064. https://doi.org/10.1002/da.22672
73. Ricciardi, J.N., McNally, R., 1995. Depressed mood is related to obsessions, but not to compulsions, in obsessive-compulsive disorder. Journal of Anxiety Disorders 9, 249–256. https://doi.org/10.1016/0887-6185(95)00006-A
74. Ronchi, P., Abbruzzese, M., Erzegovesi, S., Diaferia, G., Sciuto, G., Bellodi, L., 1992. The epidemiology of obsessive-compulsive disorder in an Italian population. European Psychiatry 7, 53–59.
75. Rozenman, M., McGuire, J., Wu, M., Ricketts, E., Peris, T., O’Neill, J., Bergman, R.L., Chang, S., Piacentini, J., 2019. Hoarding Symptoms in Children and Adolescents With Obsessive-Compulsive Disorder: Clinical Features and Response to Cognitive-Behavioral Therapy. Journal of the American Academy of Child and Adolescent Psychiatry 58, 799–805. https://doi.org/10.1016/j.jaac.2019.01.017
76. Rozenman, M., Peris, T., Bergman, R.L., Chang, S., O’Neill, J., McCracken, J.T., Piacentini, J., 2017. Distinguishing Fear Versus Distress Symptomatology in Pediatric OCD. Child psychiatry and human development 48, 63–72. https://doi.org/10.1007/s10578-016-0653-4
77. Sahmelikoglu Onur, O., Tabo, A., Aydin, E., Tuna, O., Maner, A.F., Yildirim, E.A., Çarpar, E., 2016. Relationship between impulsivity and obsession types in obsessive-compulsive disorder. International Journal of Psychiatry in Clinical Practice 20, 218–223. https://doi.org/10.1080/13651501.2016.1220580
78. Selles, R.R., Best, J.R., Stewart, S.E., 2020. Family profiles in pediatric obsessive-compulsive disorder. Journal of Obsessive-Compulsive and Related Disorders 27. https://doi.org/10.1016/j.jocrd.2020.100588
79. Sevilla-Cermeño, L., Andrén, P., Hillborg, M., Silverberg-Morse, M., Mataix-Cols, D., Fernández de la Cruz, L., 2019. Insomnia in pediatric obsessive–compulsive disorder: prevalence and association with multimodal treatment outcomes in a naturalistic clinical setting. Sleep Medicine 56, 104–110. https://doi.org/10.1016/j.sleep.2018.12.024
80. Shavitt, R.G., de Mathis, M.A., Oki, F., Ferrao, Y.A., Fontenelle, L.F., Torres, A.R., Diniz, J.B., Costa, D.L.C., do Rosário, M.C., Hoexter, M.Q., Miguel, E.C., Simpson, H.B., 2014. Phenomenology of OCD: Lessons from a large multicenter study and implications for ICD-11. Journal of Psychiatric Research 57, 141–148. https://doi.org/10.1016/j.jpsychires.2014.06.010
81. Tükel, R., Ertekin, E., Batmaz, S., Alyanak, F., Sözen, A., Aslantaş, B., Atli, H., Özyildirim, I., 2005. Influence of age of onset on clinical features in obsessive-compulsive disorder. Depression and Anxiety 21, 112–117. https://doi.org/10.1002/da.20065
82. Tükel, R., Polat, A., Genç, A., Bozkurt, O., Atli, H., 2004. Gender-related differences among Turkish patients with obsessive-compulsive disorder. Comprehensive Psychiatry 45, 362–366. https://doi.org/10.1016/j.comppsych.2004.06.006
83. Tükel, R., Polat, A., Özdemir, Ö., Aksüt, D., Türksoy, N., 2002. Comorbid conditions in obsessive-compulsive disorder. Comprehensive Psychiatry 43, 204–209. https://doi.org/10.1053/comp.2002.32355
84. van der Veld, W.M., Duppen, L., Hendriks, G.-J., Abramowitz, J.S., Kampman, M., 2020. Psychometric properties of the Dutch Dimensional Obsessive-Compulsive Scale (DOCS). Eur. J. Psychol. Assess. https://doi.org/10.1027/1015-5759/a000600
85. van Oudheusden, L.J.B., van de Schoot, R., Hoogendoorn, A., van Oppen, P., Kaarsemaker, M., Meynen, G., van Balkom, A.J.L.M., 2020. Classification of comorbidity in obsessive–compulsive disorder: A latent class analysis. Brain and Behavior. https://doi.org/10.1002/brb3.1641
86. Velloso, P., Piccinato, C., Ferrão, Y., Aliende Perin, E., Cesar, R., Fontenelle, L., Hounie, A.G., do Rosário, M.C., 2016. The suicidality continuum in a large sample of obsessive–compulsive disorder (OCD) patients. European Psychiatry 38, 1–7. https://doi.org/10.1016/j.eurpsy.2016.05.003

87. Viswanath, B., Narayanaswamy, J.C., Cherian, A.V., Reddy, Y.C.J., Math, S.B., 2011. Is familial obsessive-compulsive disorder different from sporadic obsessive-compulsive disorder? A comparison of clinical characteristics, comorbidity and treatment response. Psychopathology 44, 83–89. https://doi.org/10.1159/000317776
88. Viswanath, B., Narayanaswamy, J.C., Rajkumar, R.P., Cherian, A.V., Kandavel, T., Math, S.B., Reddy, Y.C.J., 2012. Impact of depressive and anxiety disorder comorbidity on the clinical expression of obsessive-compulsive disorder. Comprehensive Psychiatry 53, 775–782. https://doi.org/10.1016/j.comppsych.2011.10.008
89. Vorstenbosch, V., Hood, H.K., Rogojanski, J., Antony, M.M., Summerfeldt, L.J., McCabe, R.E., 2012. Exploring the relationship between OCD symptom subtypes and domains of functional impairment. Journal of Obsessive-Compulsive and Related Disorders 1, 33–40. https://doi.org/10.1016/j.jocrd.2011.10.002
90. Yanagisawa, Y., Matsuura, N., Mukai, K., Nakajima, A., Motoyama, M., Yamanishi, K., Yamada, H., Hayashida, K., Matsunaga, H., 2015. Clinically related or predictive factors and impacts on long-term treatment outcomes of involvement behaviors in patients with obsessive-compulsive disorder. Comprehensive Psychiatry 60, 105–113. https://doi.org/10.1016/j.comppsych.2015.03.002
91. Yovel, I., Gershuny, B.S., Steketee, G., Buhlmann, U., Fama, J.M., Mitchell, J., Wilhelm, S., 2012. Idiosyncratic severity profiles of obsessive compulsive dysfunction: A detailed self report assessment of a multifaceted syndrome. Cognitive Therapy and Research 36, 694–713. https://doi.org/10.1007/s10608-011-9427-2
