# Supplementary material for: Comorbidities in Obsessive-Compulsive Disorder Across the Lifespan: A Systematic Review and Meta-Analysis
Source: Front Psychiatry. 2021 Nov 11;12:703701. doi: 10.3389/fpsyt.2021.703701 (PMC8631971; doi:10.3389/fpsyt.2021.703701)

OCD Comorbidity Meta-analysis: Across the Lifespan

Sharma E et al

26/07/2021

Quality Check (QC) for each disorder

The numbers in the Baujat plots indicate the Study No in the corresponding table for the particular co-morbidity in Supplementary File 1.

Studies marked in red in the influence plots indicate outliers that significantly influence the pooled estimate and are excluded from the final analysis.

Suppl Figure 1.1 QC Plots for Any psychiatric comorbidity

Consider excluding the following study(s), based on the Baujat & Influence Plots: None

Baujat Plot

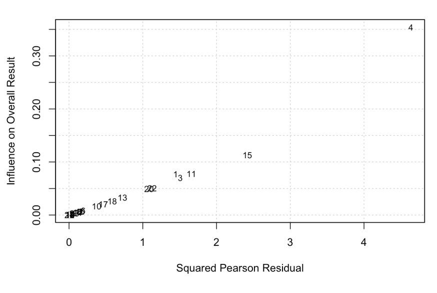

Influence Plots

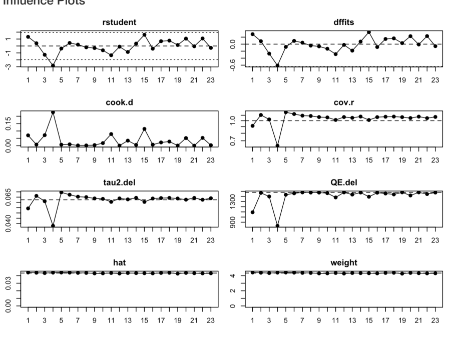

Suppl Figure 1.2 QC Plots for Any mood disorder

Consider excluding the following study(s), based on the Baujat & Influence Plots: Calamari 2008

Baujat Plot

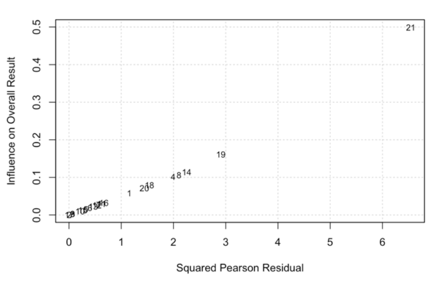

Influence Plots

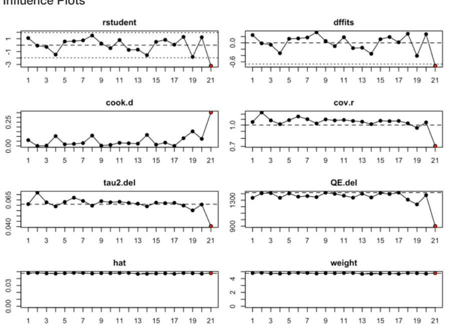

Suppl Figure 1.3 QC Plots for Major Depressive Disorder

Consider excluding the following study(s), based on the Baujat & Influence Plots: None

Baujat Plot

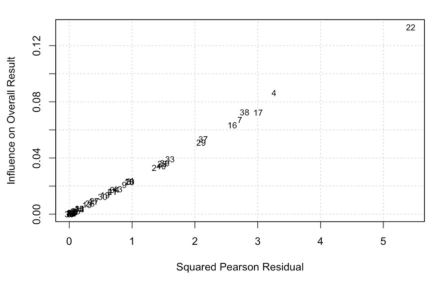

Influence Plots

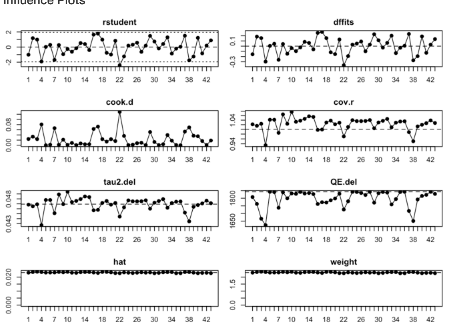

Suppl Figure 1.4 QC Plots for Dysthymia

Consider excluding the following study(s), based on the Baujat & Influence Plots: Fals-Stewart 1993

Baujat Plot

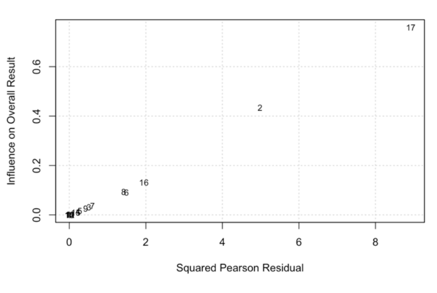

Influence Plots

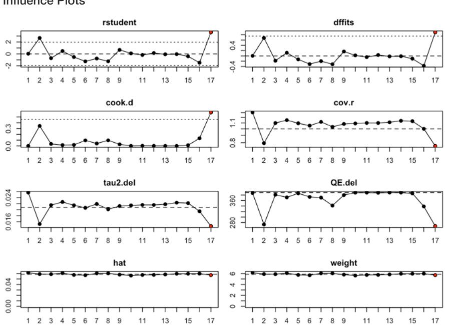

Suppl Figure 1.5 QC Plots for Bipolar Disorder

Consider excluding the following study(s), based on the Baujat & Influence Plots: None

Baujat Plot

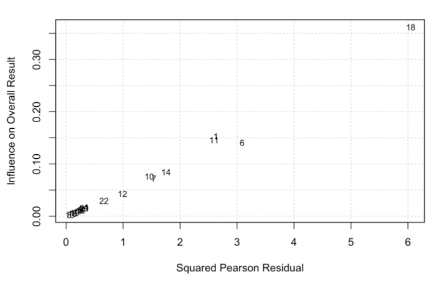

Influence Plots

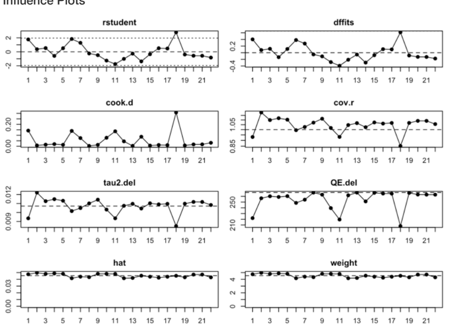

Suppl Figure 1.6 QC Plots for Schizophrenia/Any psychotic disorder

Consider excluding the following study(s), based on the Baujat & Influence Plots: None

Baujat Plot

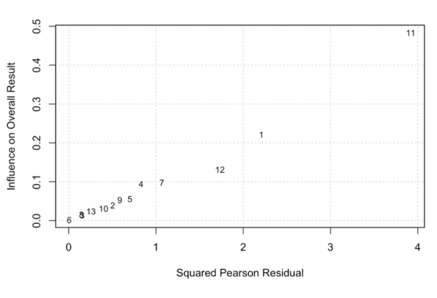

Influence Plots

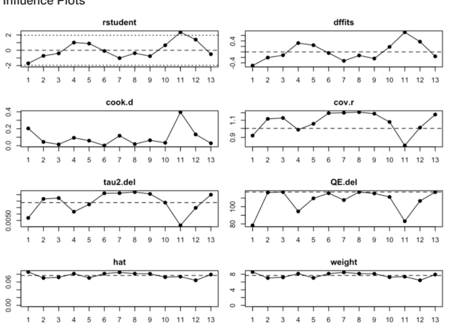

Suppl Figure 1.7 QC Plots for Any anxiety disorder

Consider excluding the following study(s), based on the Baujat & Influence Plots: None

Baujat Plot

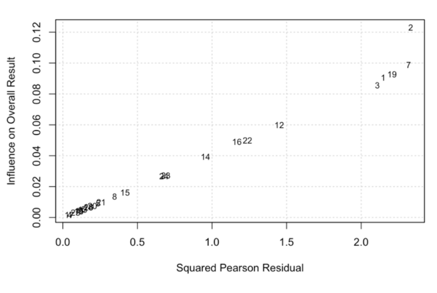

Influence Plots

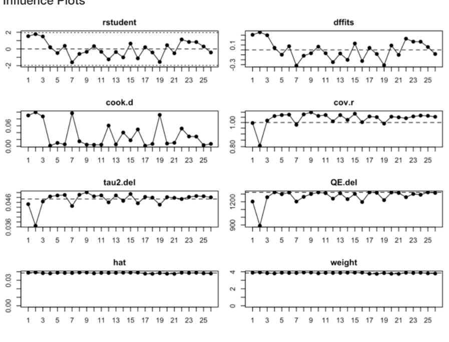

Suppl Figure 1.8 QC Plots for Generalised Anxiety Disorder

Consider excluding the following study(s), based on the Baujat & Influence Plots: None

Baujat Plot

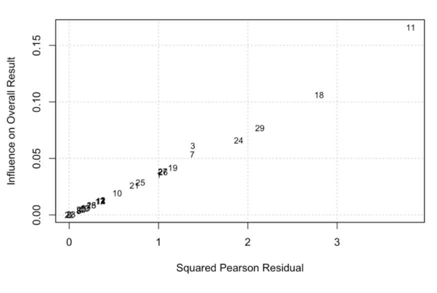

Influence Plots

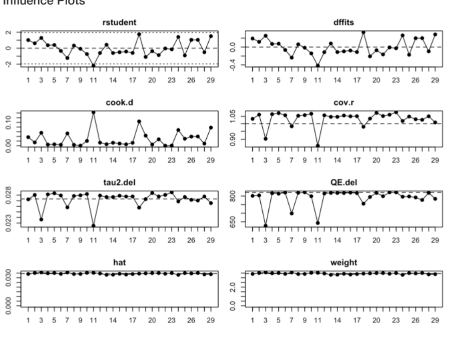

Suppl Figure 1.9 QC Plots for Social Anxiety Disorder

Consider excluding the following study(s), based on the Baujat & Influence Plots: None

Baujat Plot

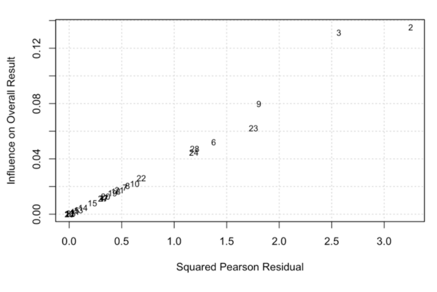

Influence Plots

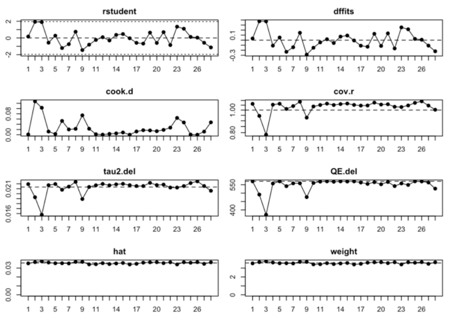

Suppl Figure 1.10 QC Plots for Panic Disorder

Consider excluding the following study(s), based on the Baujat & Influence Plots: None

Baujat Plot

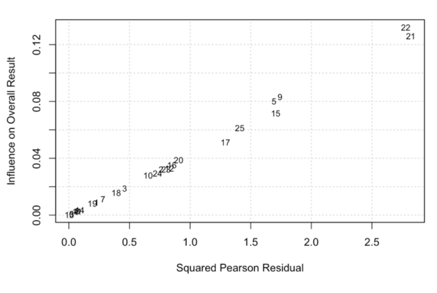

Influence Plots

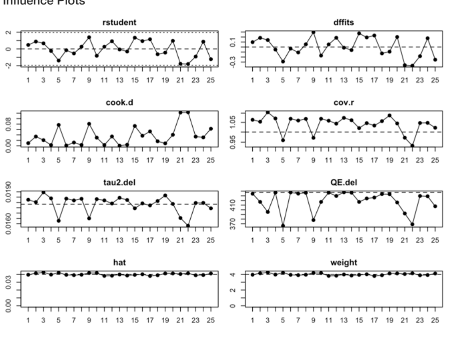

Suppl Figure 1.11 QC Plots for Agoraphobia

Consider excluding the following study(s), based on the Baujat & Influence Plots: Geller 2000

Baujat Plot

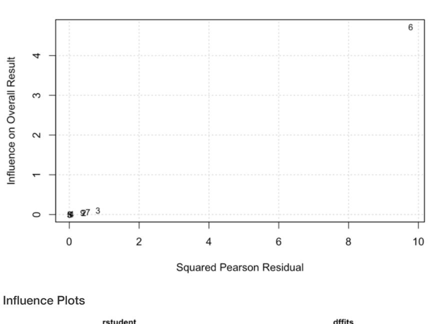

Influence Plots

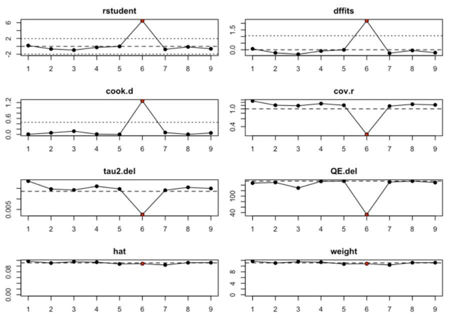

Suppl Figure 1.12 QC Plots for Simple/Specific Phobia

Consider excluding the following study(s), based on the Baujat & Influence Plots: [illegible]

Baujat Plot

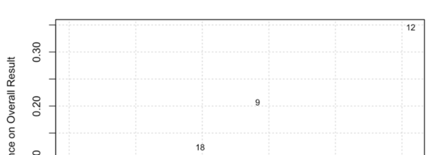

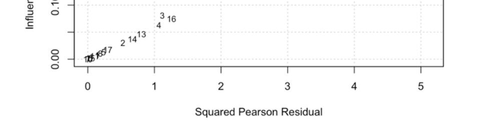

Influence Plots

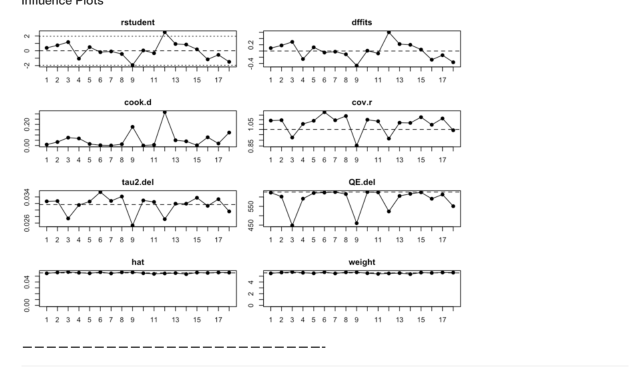

Suppl Figure 1.13 QC Plots for Somatic symptom disorder/Somatoform/Somatization disorder

Consider excluding the following study(s), based on the Baujat & Influence Plots: None

Baujat Plot

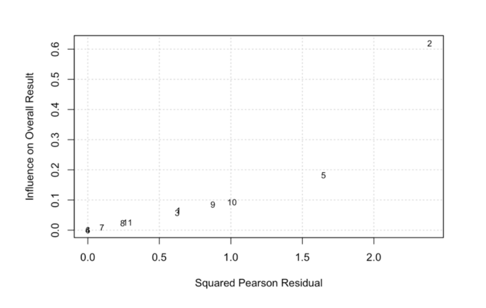

Influence Plots

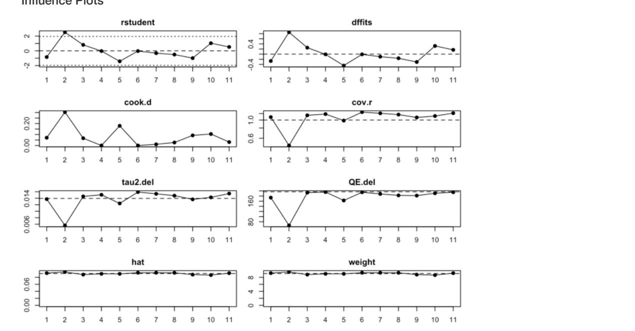

Suppl Figure 1.14 QC Plots for Hypochondriasis/Illness anxiety disorder

Consider excluding the following study(s), based on the Baujat & Influence Plots: None

Baujat Plot

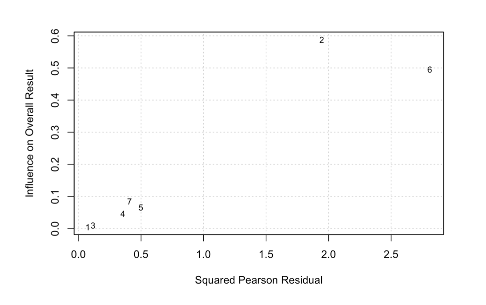

Influence Plots

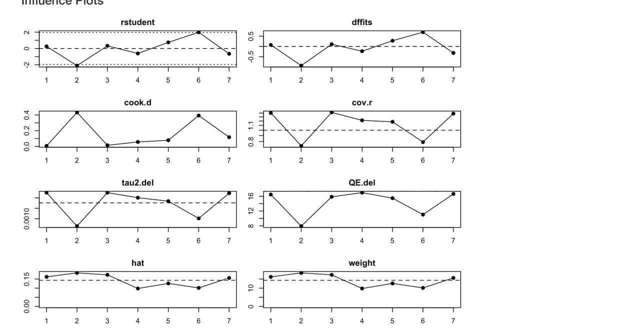

Suppl Figure 1.15 QC Plots for Post-traumatic stress disorder (PTSD)

Consider excluding the following study(s), based on the Baujat & Influence Plots: Merrill 2011

Baujat Plot

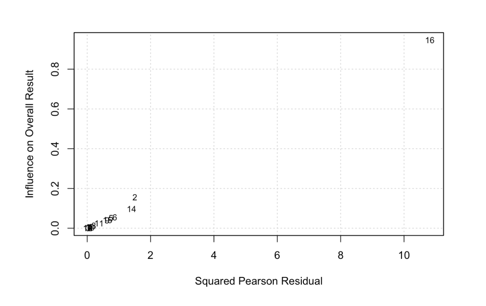

Influence Plots

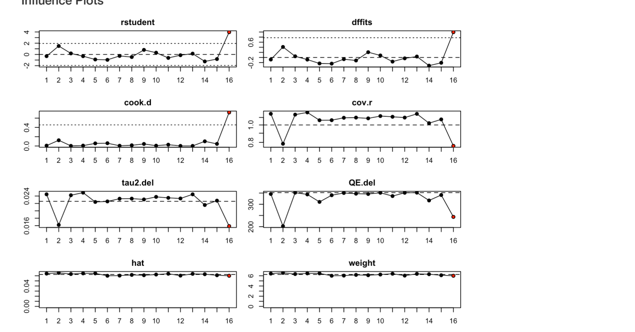

Suppl Figure 1.16 QC Plots for Any obsessive compulsive related (spectrum) disorders

Consider excluding the following study(s), based on the Baujat & Influence Plots: None

Baujat Plot

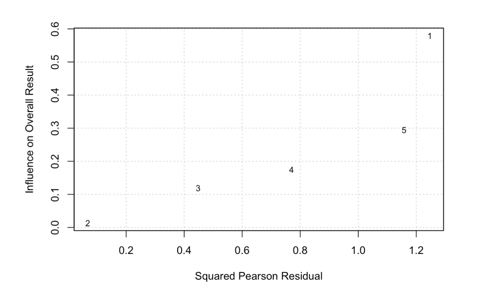

Influence Plots

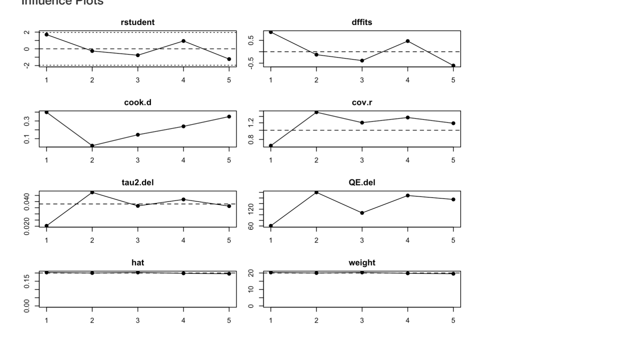

Suppl Figure 1.17 QC Plots for Body dysmorphic disorder

Consider excluding the following study(s), based on the Baujat & Influence Plots: deMathis 2008

Baujat Plot

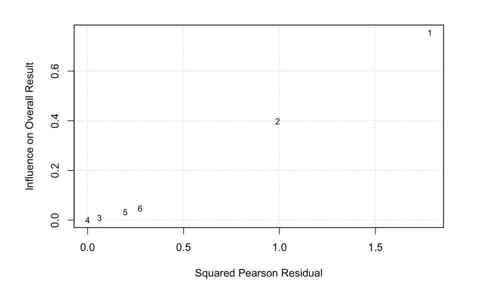

Influence Plots

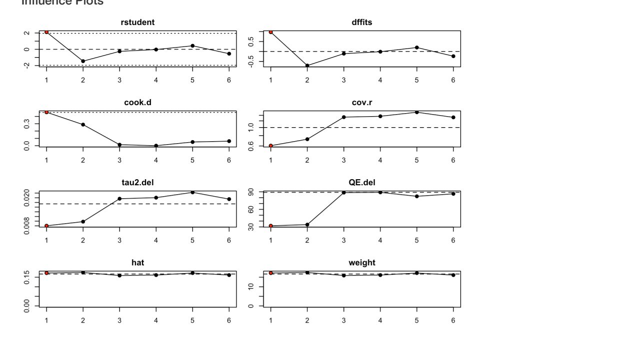

Suppl Figure 1.18 QC Plots for Trichotillomania

Consider excluding the following study(s), based on the Baujat & Influence Plots: None

Baujat Plot

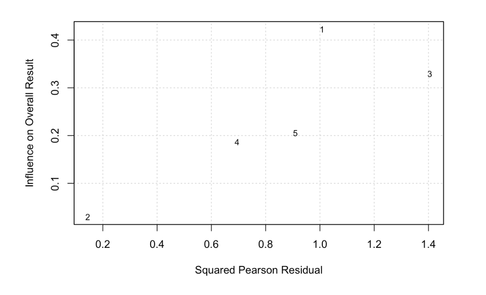

Influence Plots

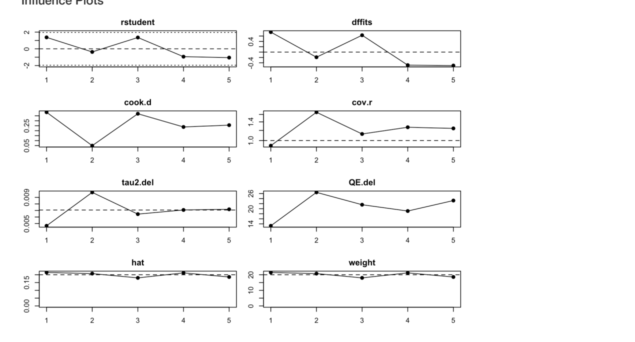

Suppl Figure 1.19 QC Plots for Any tic disorder

Consider excluding the following study(s), based on the Baujat & Influence Plots: None

Baujat Plot

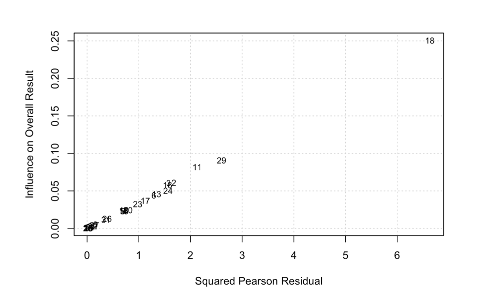

Influence Plots

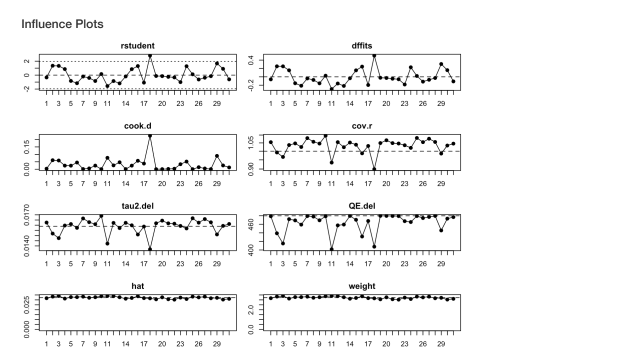

Suppl Figure 1.20 QC Plots for Any eating disorder

Consider excluding the following study(s), based on the Baujat & Influence Plots: Viswanath 2012

Baujat Plot

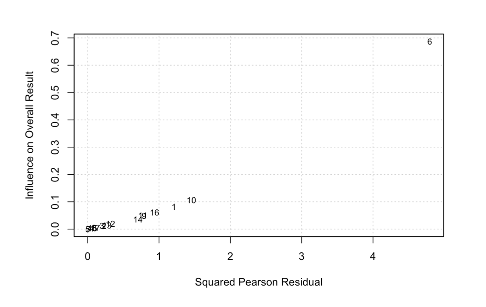

Influence Plots

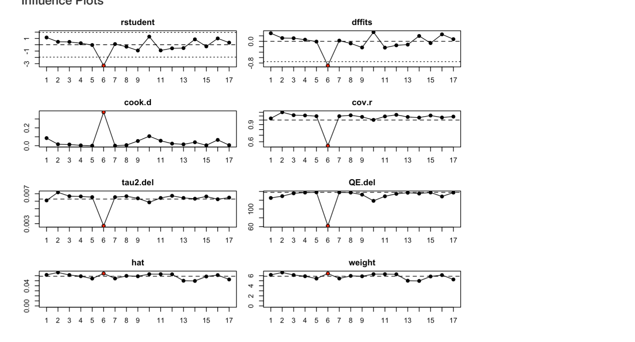

Suppl Figure 1.21 QC Plots for Anorexia nervosa

Consider excluding the following study(s), based on the Baujat & Influence Plots: None

Baujat Plot

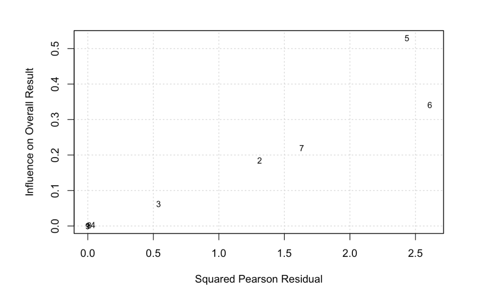

Influence Plots

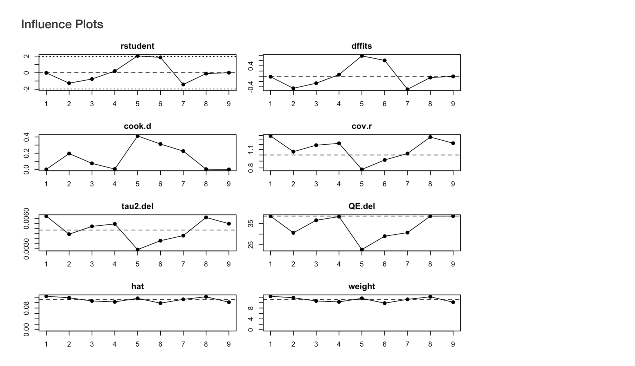

Suppl Figure 1.22 QC Plots for Bulimia nervosa

Consider excluding the following study(s), based on the Baujat & Influence Plots: Tukel 2004

Baujat Plot

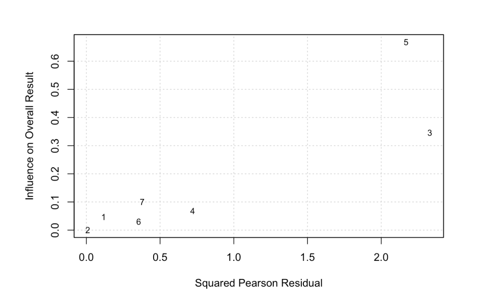

Influence Plots

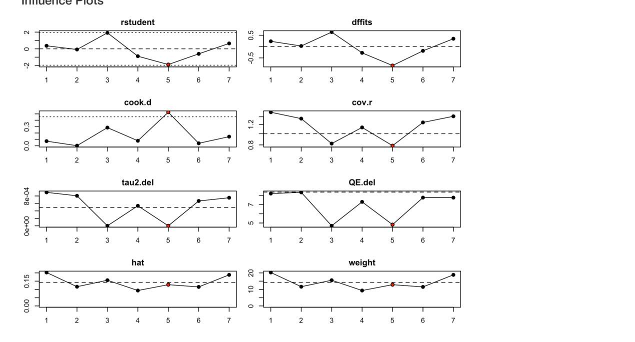

Suppl Figure 1.23 QC Plots for Any substance use disorder

Consider excluding the following study(s), based on the Baujat & Influence Plots: Mancebo 2009

Baujat Plot

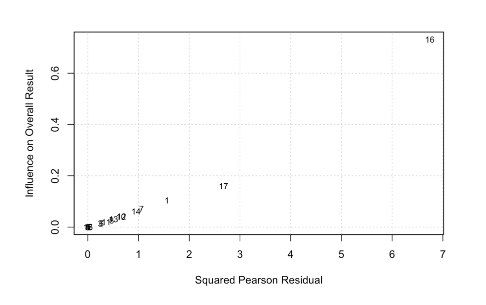

Influence Plots

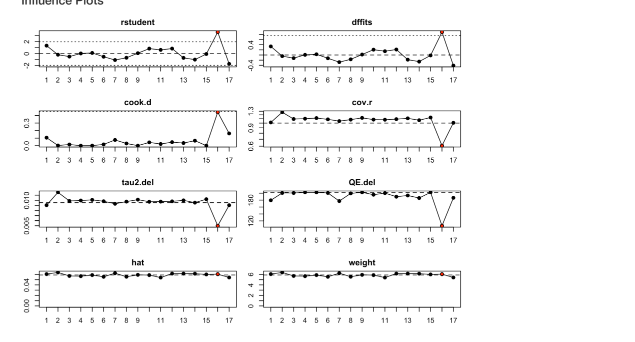

Suppl Figure 1.24 QC Plots for Autism spectrum disorder

Consider excluding the following study(s), based on the Baujat & Influence Plots: Sevilla-Cermeno 2019

Baujat Plot

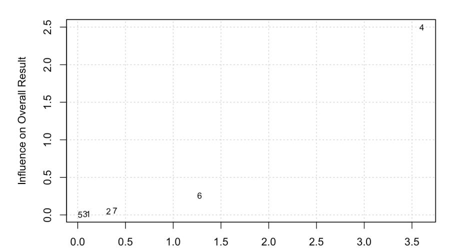

Influence Plots

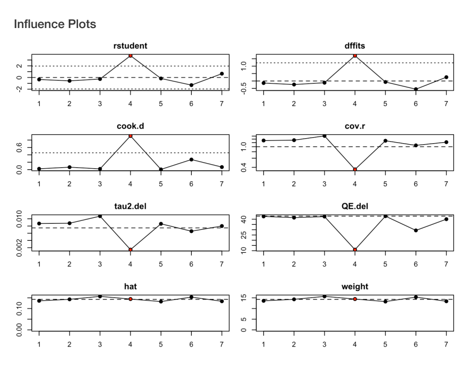

Suppl Figure 1.25 QC Plots for Attention deficit hyperactivity disorder

Consider excluding the following study(s), based on the Baujat & Influence Plots: Geller 2000

Baujat Plot

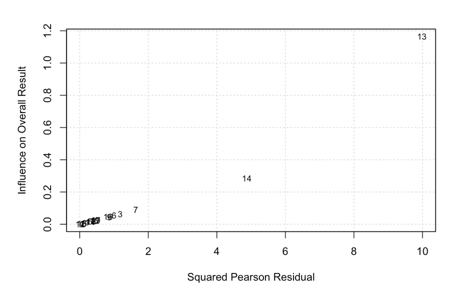

Influence Plots

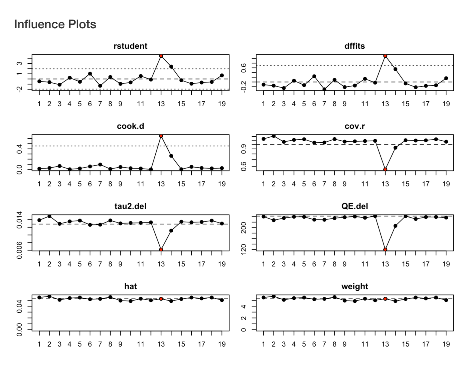

Suppl Figure 1.26 QC Plots for Oppositional defiant disorder

Consider excluding the following study(s), based on the Baujat & Influence Plots: None

Baujat Plot

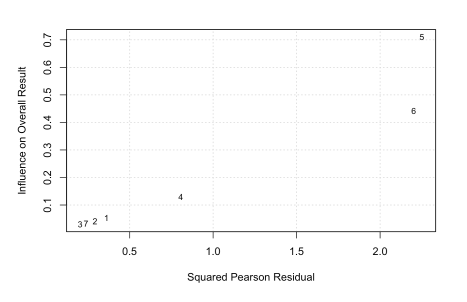

Influence Plots

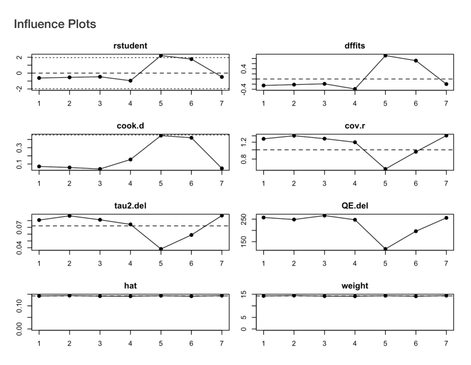

Suppl Figure 1.27 QC Plots for Any personality disorder

Consider excluding the following study(s), based on the Baujat & Influence Plots: None

Baujat Plot

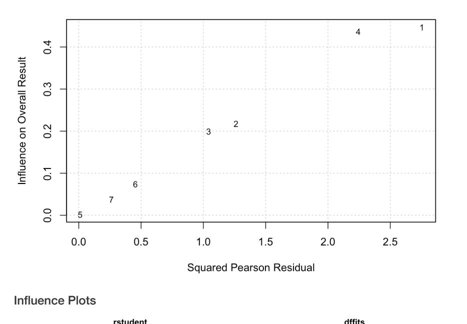

Influence Plots

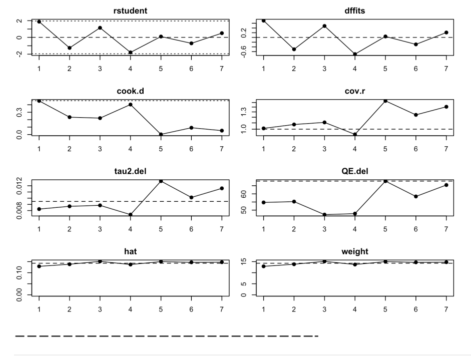

Suppl Figure 1.28 QC Plots for Obsessive compulsive (Anankastic) personality disorder

Consider excluding the following study(s), based on the Baujat & Influence Plots: Lochner 2004

Baujat Plot

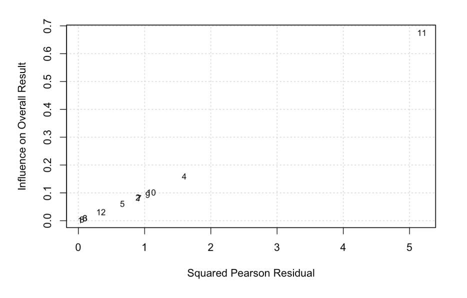

Influence Plots

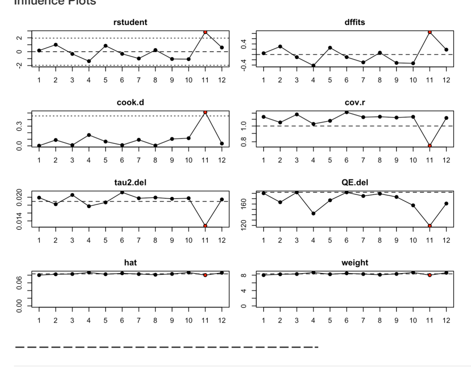

Suppl Figure 1.29 QC Plots for Schizoid personality disorder

Consider excluding the following study(s), based on the Baujat & Influence Plots: Gordon 2013

Baujat Plot

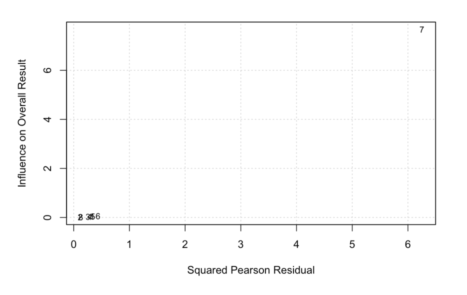

Influence Plots

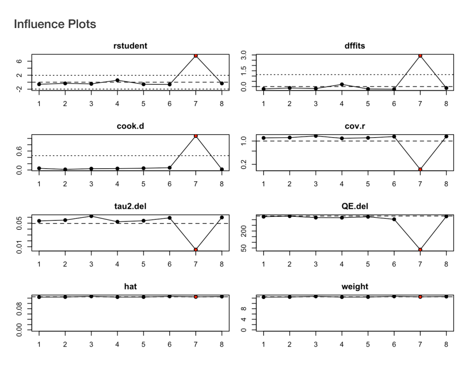

Suppl Figure 1.30 QC Plots for Schizotypal personality disorder

Consider excluding the following study(s), based on the Baujat & Influence Plots: None

Baujat Plot

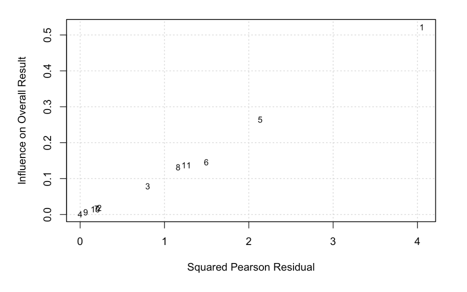

Influence Plots

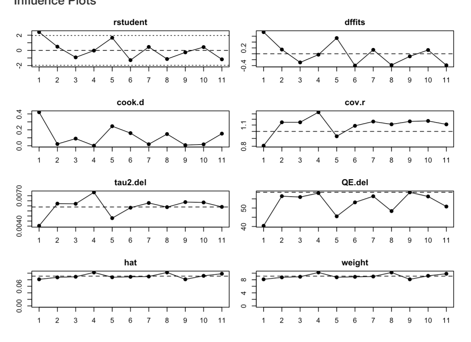

Suppl Figure 1.31 QC Plots for Borderline personality disorder

Consider excluding the following study(s), based on the Baujat & Influence Plots: None

Baujat Plot

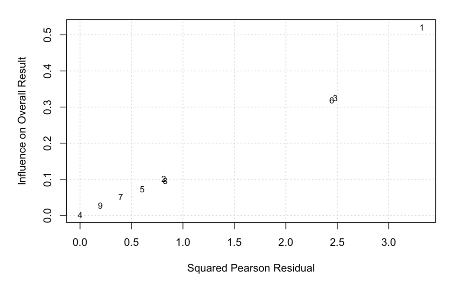

Influence Plots

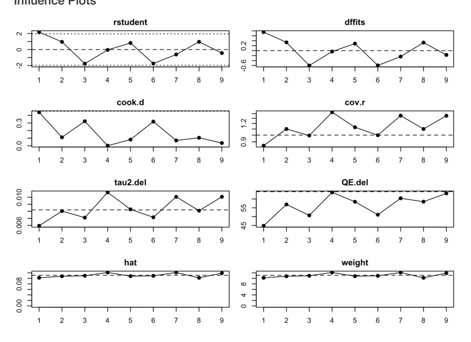

Suppl Figure 1.32 QC Plots for Histrionic personality disorder

Consider excluding the following study(s), based on the Baujat & Influence Plots: Gordon 2013

Baujat Plot

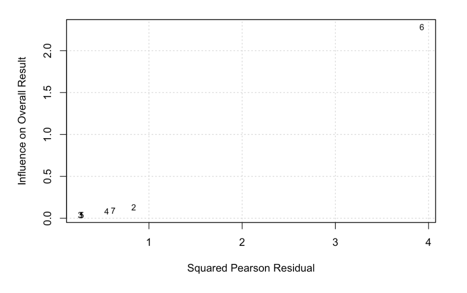

Influence Plots

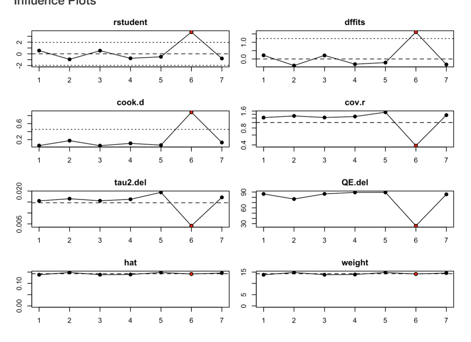

Suppl Figure 1.33 QC Plots for Antisocial personality disorder

Consider excluding the following study(s), based on the Baujat & Influence Plots: Maina 2008

Baujat Plot

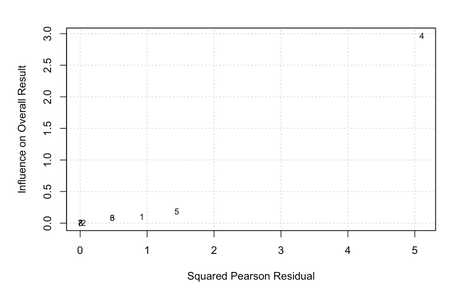

Influence Plots

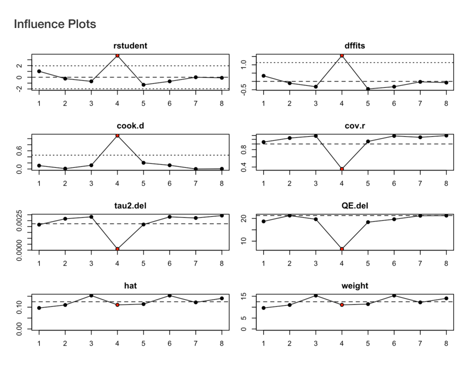

Suppl Figure 1.34 QC Plots for Narcissistic personality disorder

Consider excluding the following study(s), based on the Baujat & Influence Plots: None

Baujat Plot

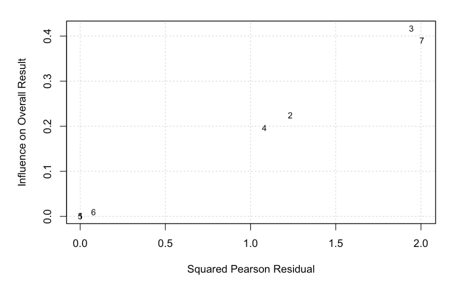

Influence Plots

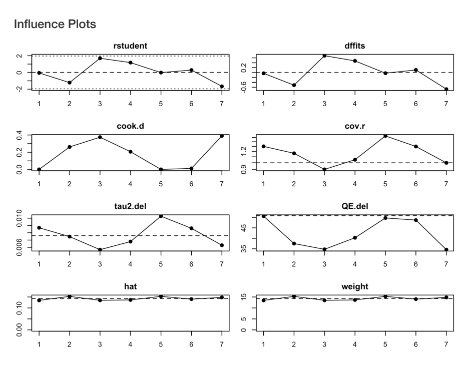

Suppl Figure 1.35 QC Plots for Dependent personality disorder

Consider excluding the following study(s), based on the Baujat & Influence Plots: None

Baujat Plot

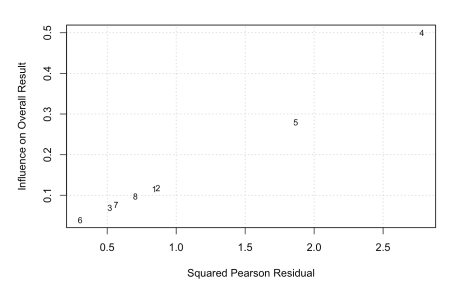

Influence Plots

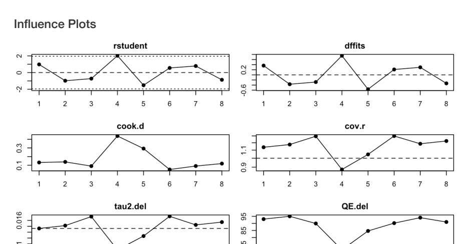

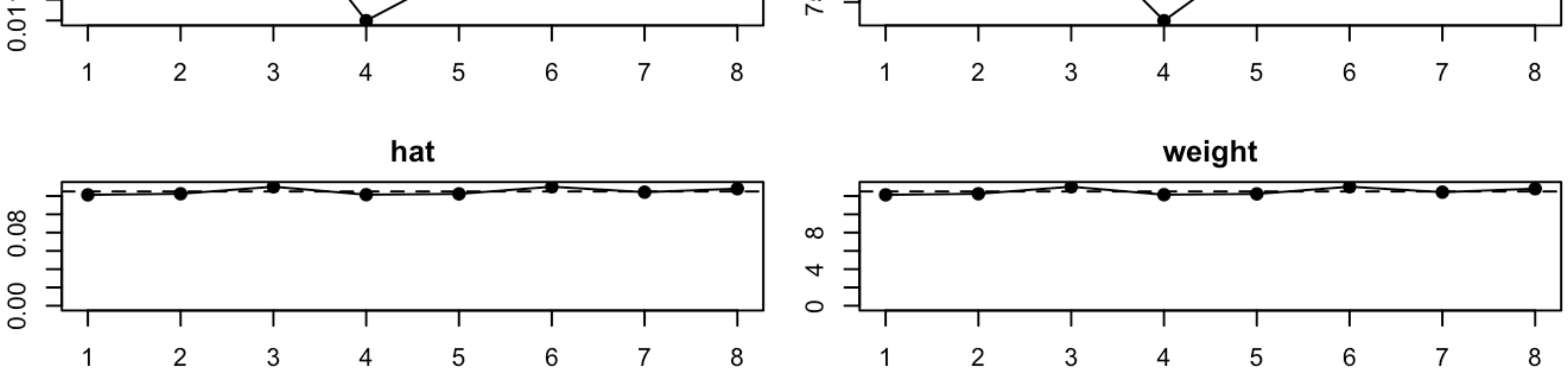

Suppl Figure 1.36 QC Plots for Anxious-avoidant personality disorder

Consider excluding the following study(s), based on the Baujat & Influence Plots: None

Baujat Plot

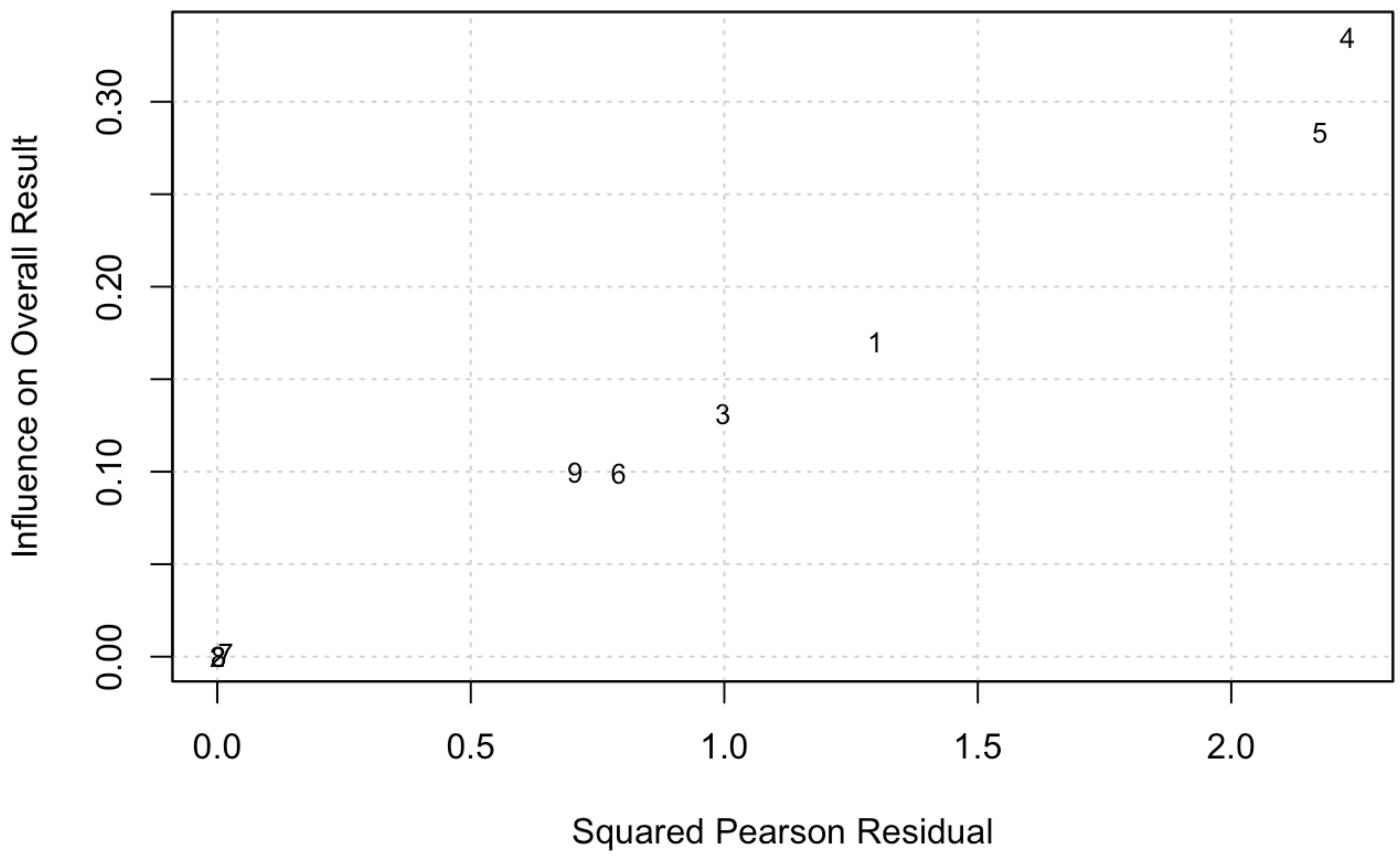

Influence Plots

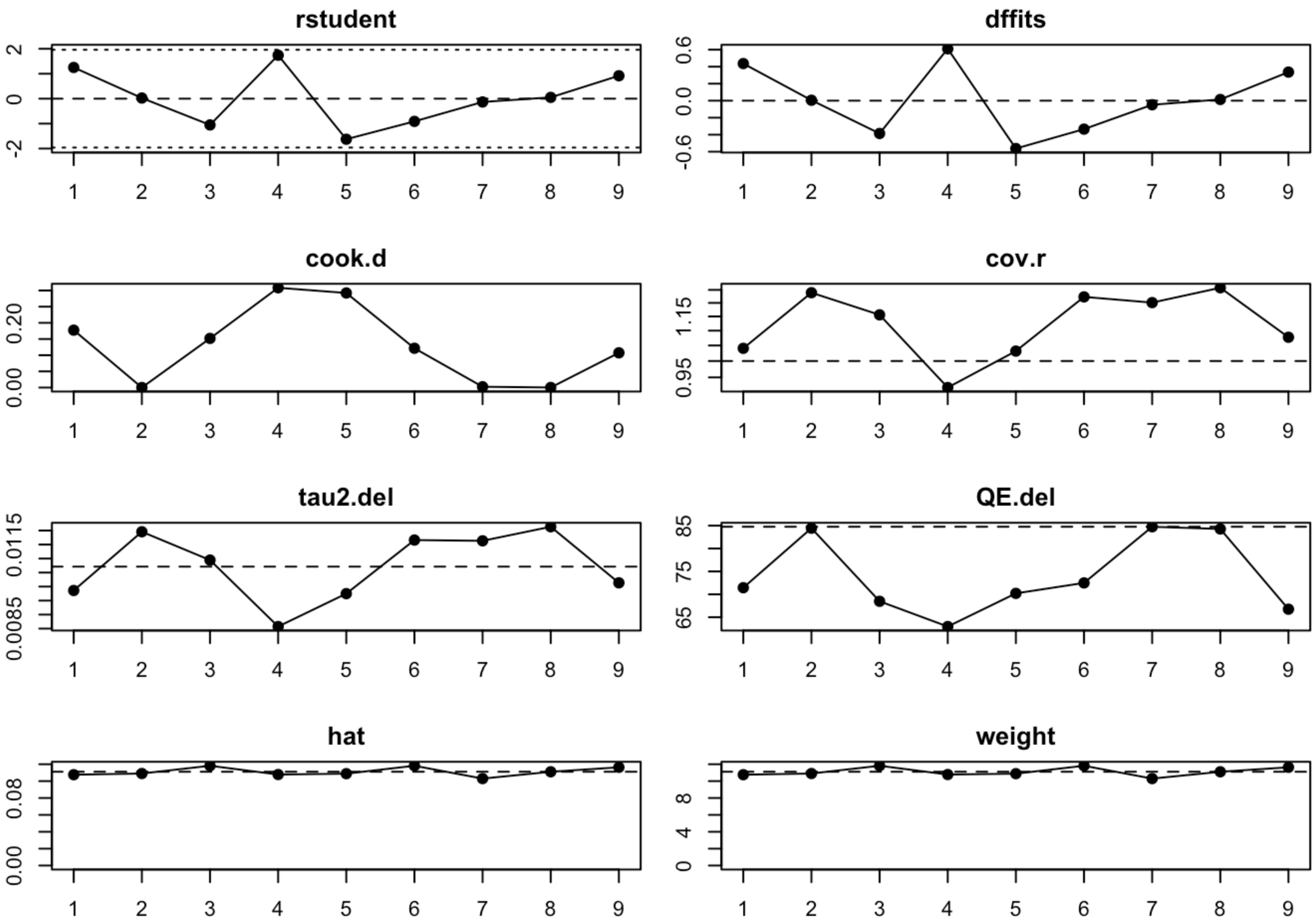

Supplement: Supplementary file 2 [file Data_Sheet_2.PDF]
