# Supplementary material for: Comorbidities in Obsessive-Compulsive Disorder Across the Lifespan: A Systematic Review and Meta-Analysis
Source: Front Psychiatry. 2021 Nov 11;12:703701. doi: 10.3389/fpsyt.2021.703701 (PMC8631971; doi:10.3389/fpsyt.2021.703701)

OCD Comorbidity Meta-analysis: Across the Lifespan

Sharma E et al.

26/07/2021

Forest plots for each disorder

Method: Random-effects meta-analysis of proportions with double-arcsine transformation

Suppl Figure 2.1.1 Forest Plot for Any psychiatric comorbidity

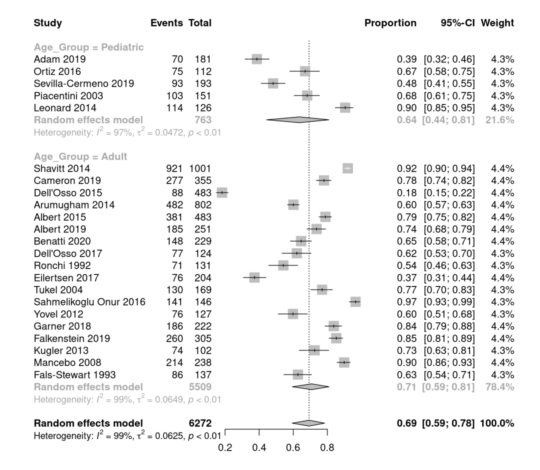

Suppl Figure 2.1.2 Funnel Plot for Any psychiatric comorbidity

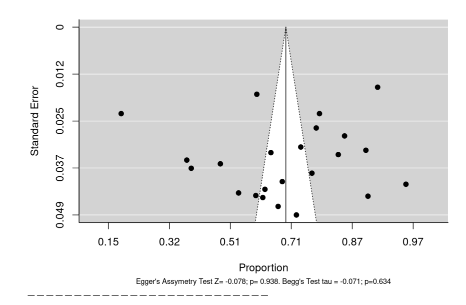

Suppl Figure 2.2.1 Forest Plot for Any mood disorder

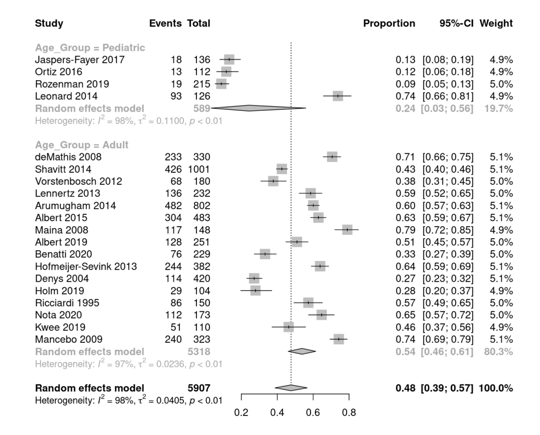

Suppl Figure 2.2.2 Funnel Plot for Any mood disorder

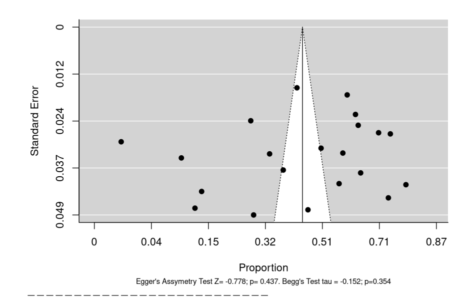

Suppl Figure 2.3.1 Forest Plot for Major Depressive Disorder

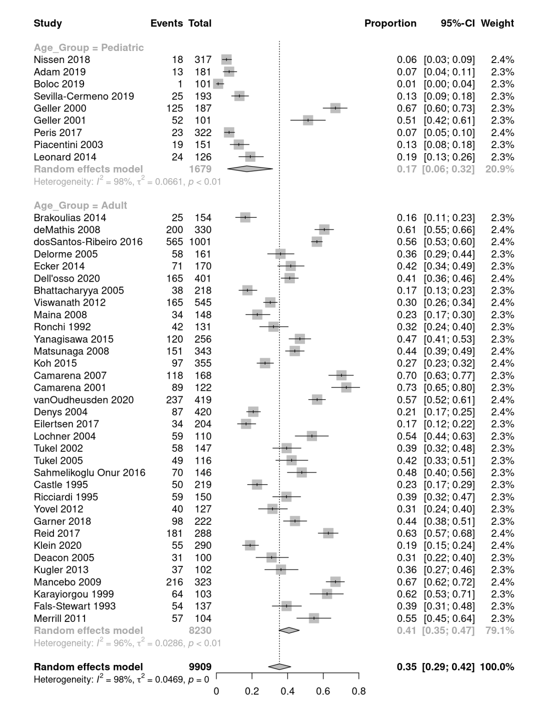

Suppl Figure 2.3.2 Funnel Plot for Major Depressive Disorder

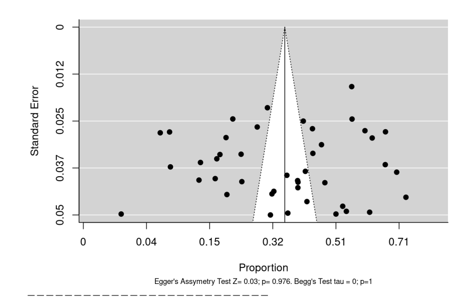

Suppl Figure 2.4.1 Forest Plot for Dysthymia

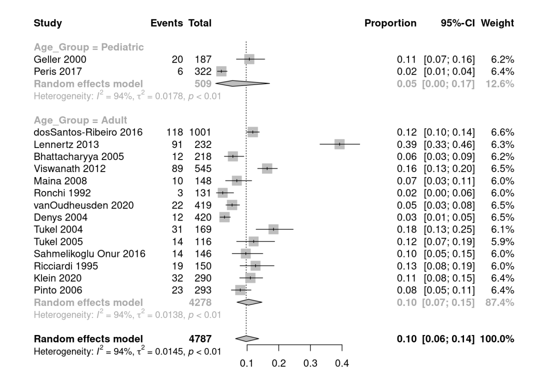

Suppl Figure 2.4.2 Funnel Plot for Dysthymia

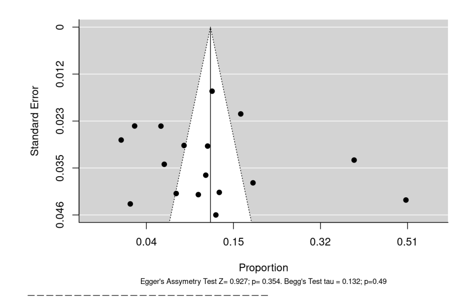

Suppl Figure 2.5.1 Forest Plot for Bipolar Disorder

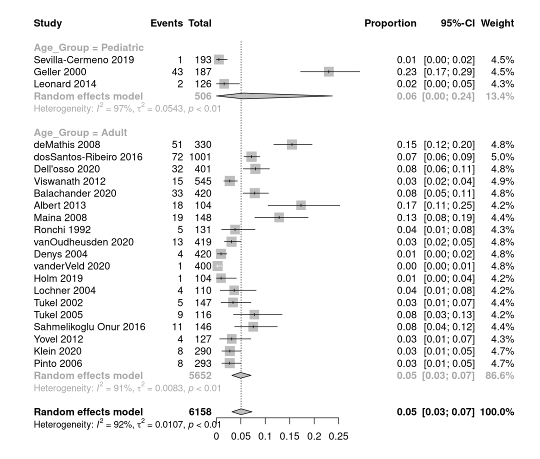

Suppl Figure 2.5.2 Funnel Plot for Bipolar Disorder

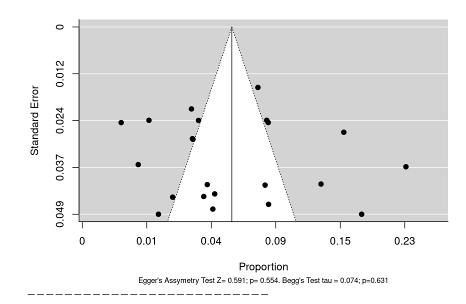

Suppl Figure 2.6.1 Forest Plot for Schizophrenia/Any psychotic disorder

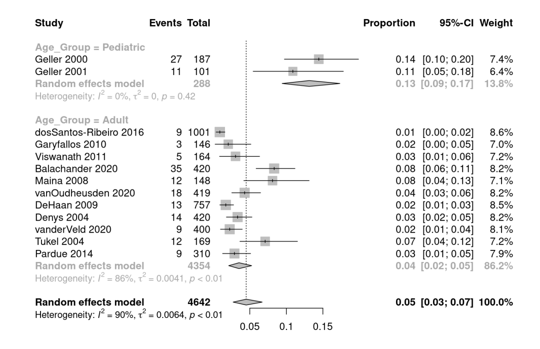

Suppl Figure 2.6.2 Funnel Plot for Schizophrenia/Any psychotic disorder

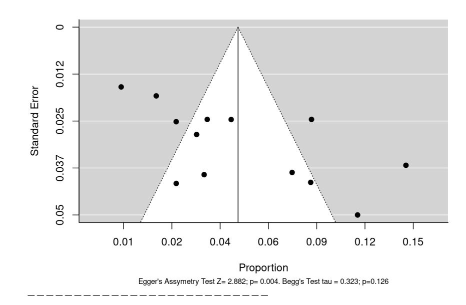

Suppl Figure 2.7.1 Forest Plot for Any anxiety disorder

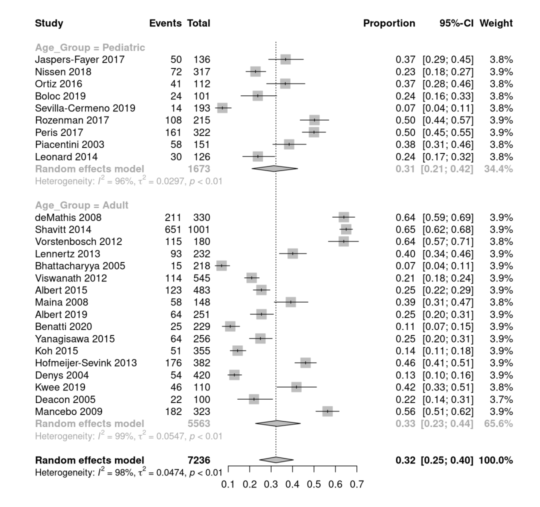

Suppl Figure 2.7.2 Funnel Plot for Any anxiety disorder

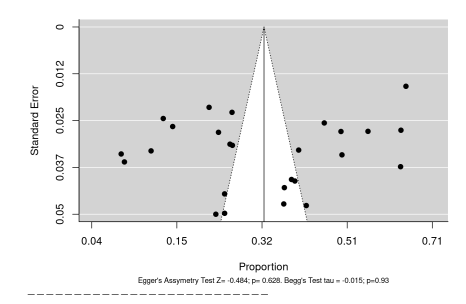

Suppl Figure 2.8.1 Forest Plot for Generalised Anxiety Disorder

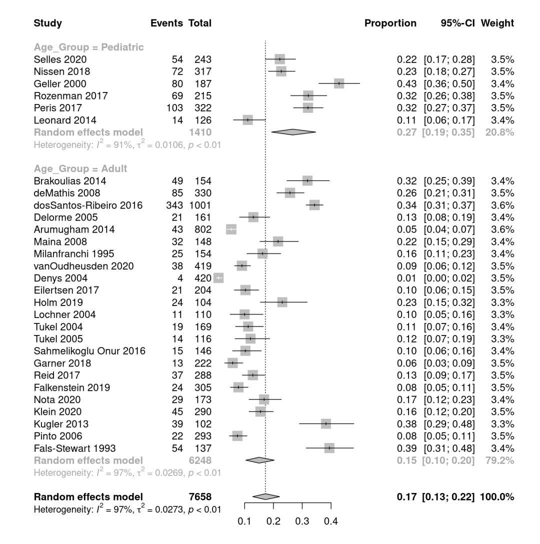

Suppl Figure 2.8.2 Funnel Plot for Generalised Anxiety Disorder

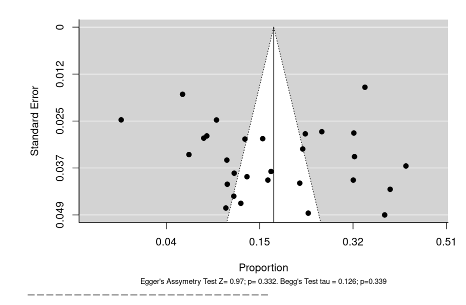

Suppl Figure 2.9.1 Forest Plot for Social Anxiety Disorder

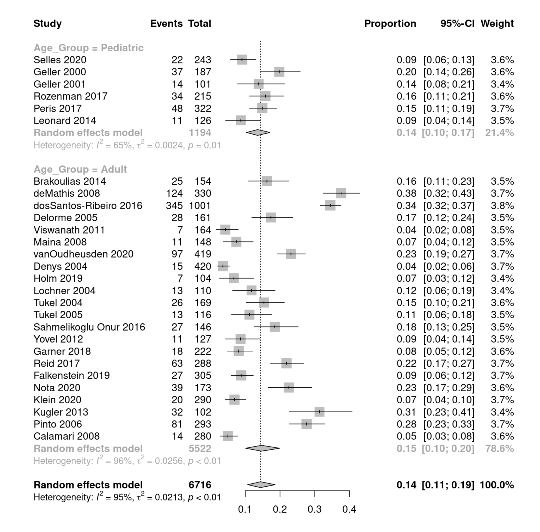

Suppl Figure 2.9.2 Funnel Plot for Social Anxiety Disorder

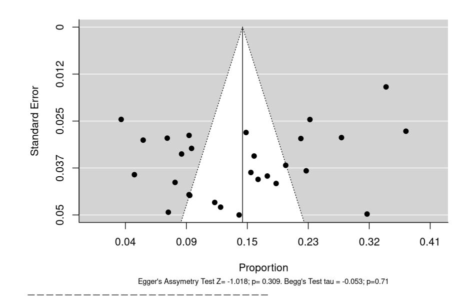

Suppl Figure 2.10.1 Forest Plot for Panic Disorder

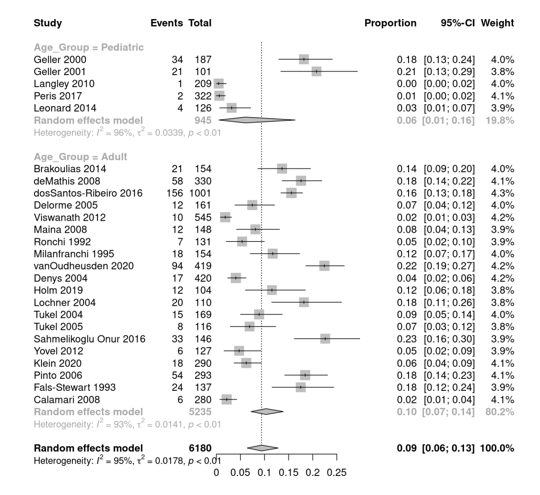

Suppl Figure 2.10.2 Funnel Plot for Panic Disorder

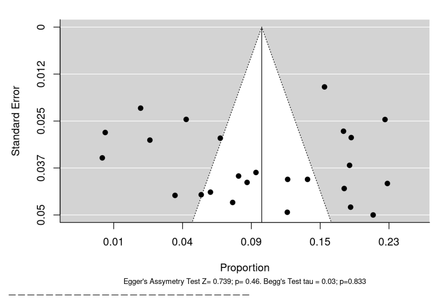

Suppl Figure 2.11.1 Forest Plot for Agoraphobia

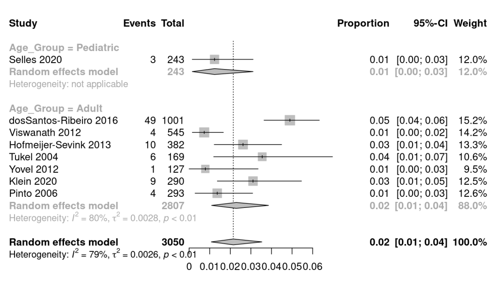

Suppl Figure 2.11.2 Funnel Plot for Agoraphobia

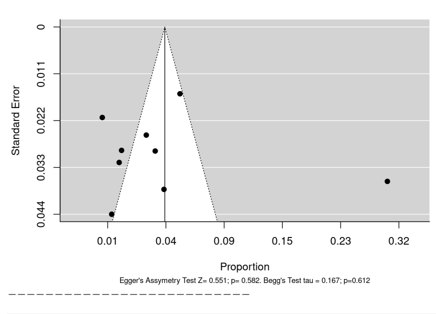

Suppl Figure 2.12.1 Forest Plot for Simple/Specific Phobia

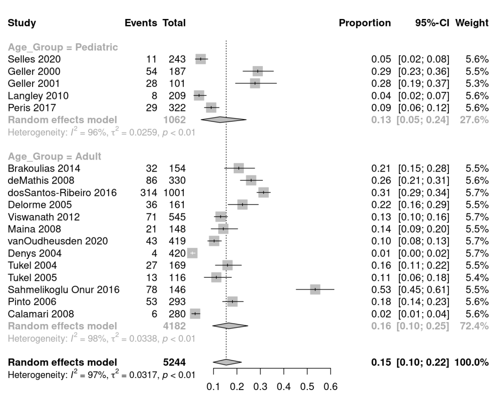

Suppl Figure 2.12.2 Funnel Plot for Simple/Specific Phobia

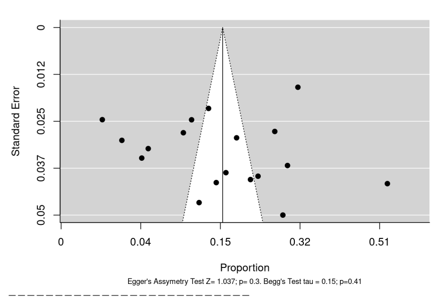

Suppl Figure 2.13.1 Forest Plot for Somatic symptom disorder/Somatoform/Somatization disorder

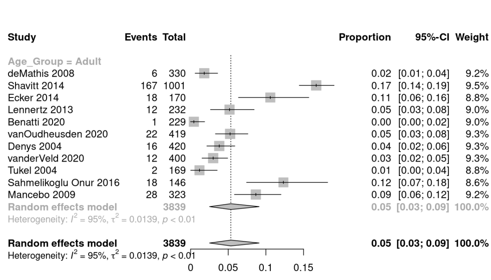

Suppl Figure 2.13.2 Funnel Plot for Somatic symptom disorder/Somatoform/Somatization disorder

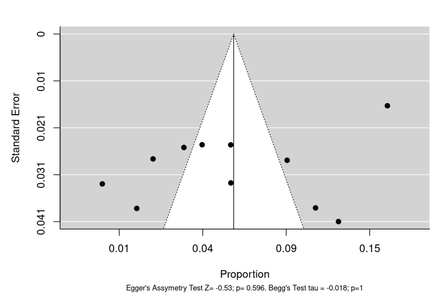

Suppl Figure 2.14.1 Forest Plot for Hypochondriasis/Illness anxiety disorder

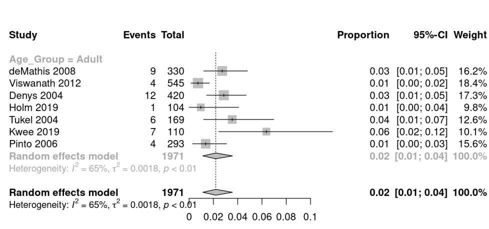

Suppl Figure 2.14.2 Funnel Plot for Hypochondriasis/Illness anxiety disorder

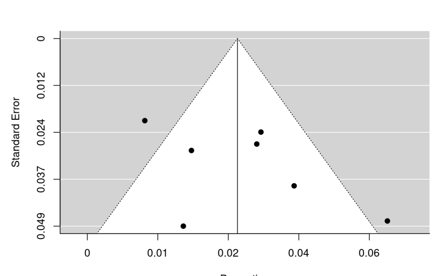

Suppl Figure 2.15.1 Forest Plot for Post-traumatic stress disorder (PTSD)

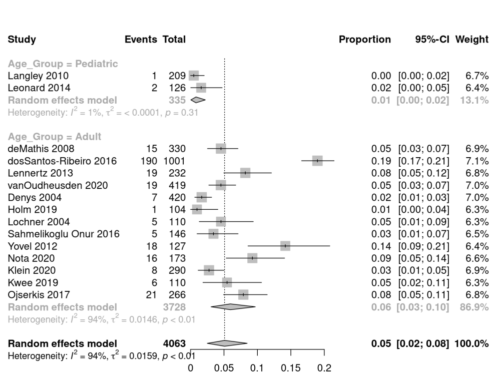

Suppl Figure 2.15.2 Funnel Plot for Post-traumatic stress disorder (PTSD)

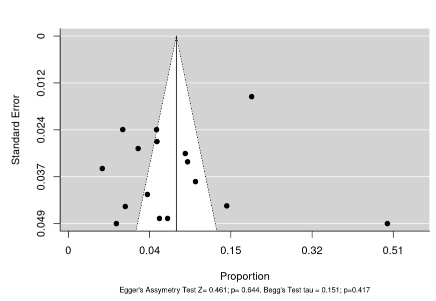

Suppl Figure 2.16.1 Forest Plot for Any obsessive compulsive related (spectrum) disorders

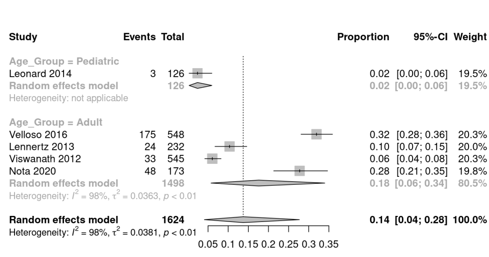

Suppl Figure 2.16.2 Funnel Plot for Any obsessive compulsive related (spectrum) disorders

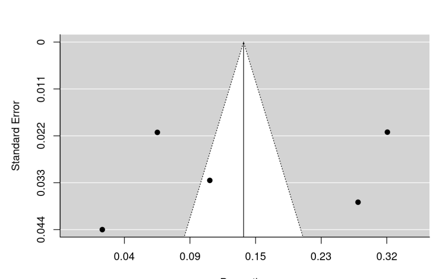

Suppl Figure 2.17.1 Forest Plot for Body dysmorphic disorder

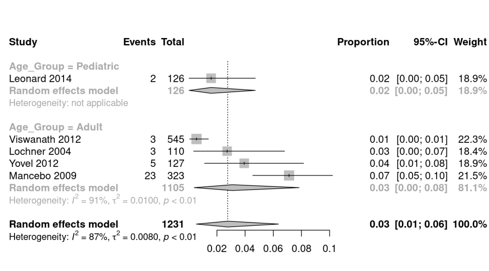

Suppl Figure 2.17.2 Funnel Plot for Body dysmorphic disorder

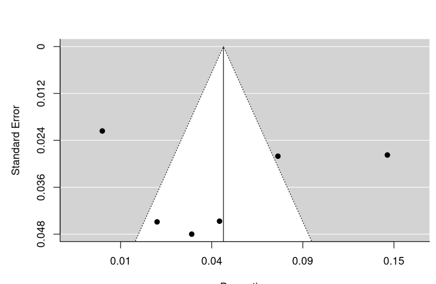

Suppl Figure 2.18.1 Forest Plot for Trichotillomania

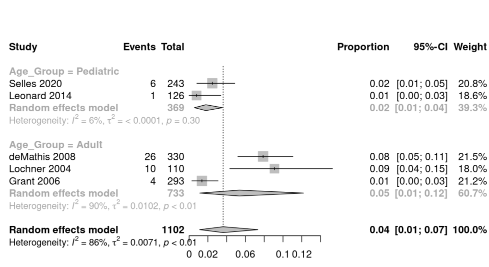

Suppl Figure 2.18.2 Funnel Plot for Trichotillomania

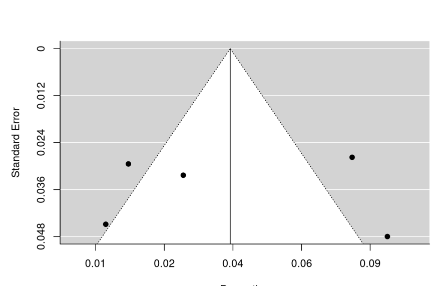

Suppl Figure 2.19.1 Forest Plot for Any tic disorder

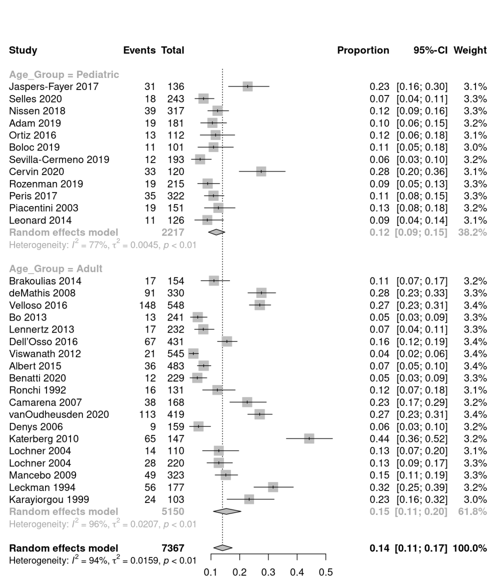

Suppl Figure 2.19.2 Funnel Plot for Any tic disorder

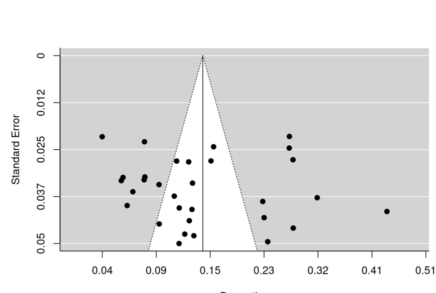

Suppl Figure 2.20.1 Forest Plot for Any eating disorder

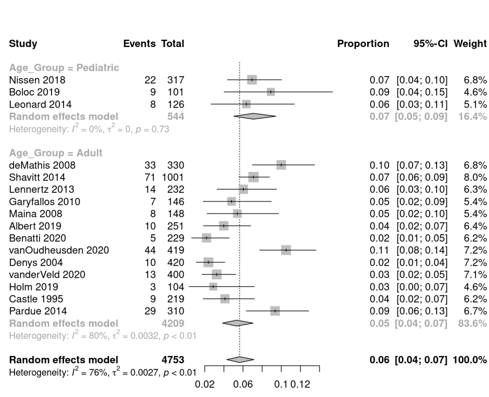

Suppl Figure 2.20.2 Funnel Plot for Any eating disorder

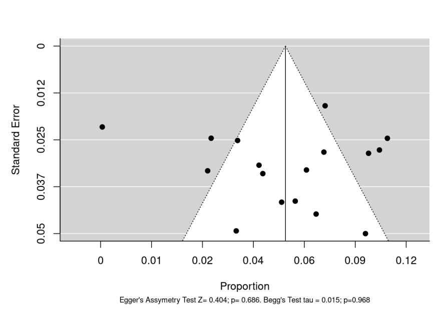

Suppl Figure 2.21.1 Forest Plot for Anorexia nervosa

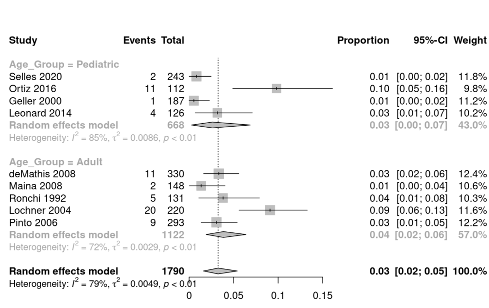

Suppl Figure 2.21.2 Funnel Plot for Anorexia nervosa

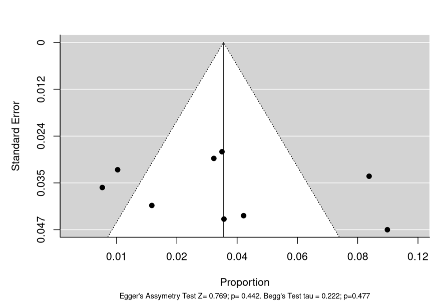

Suppl Figure 2.22.1 Forest Plot for Bulimia nervosa

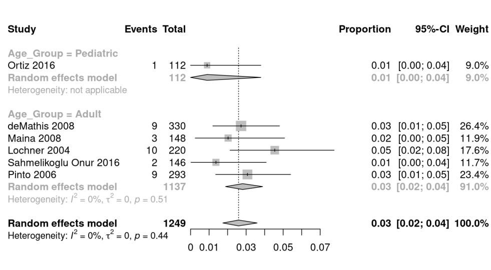

Suppl Figure 2.22.2 Funnel Plot for Bulimia nervosa

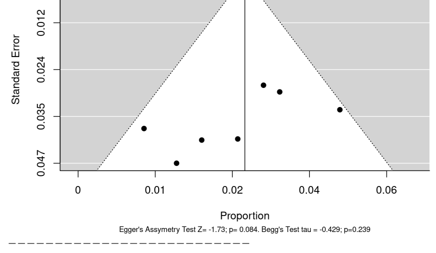

Suppl Figure 2.23.1 Forest Plot for Any substance use disorder

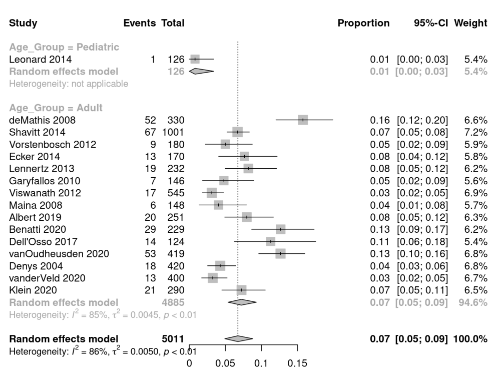

Suppl Figure 2.23.2 Funnel Plot for Any substance use disorder

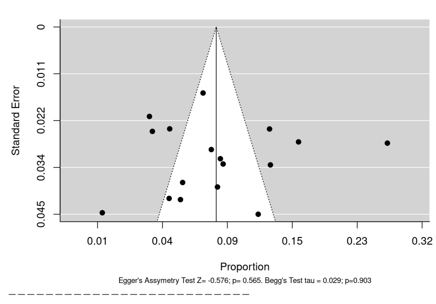

Suppl Figure 2.24.1 Forest Plot for Autism spectrum disorder

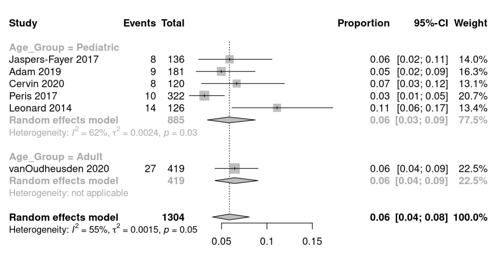

Suppl Figure 2.24.2 Funnel Plot for Autism spectrum disorder

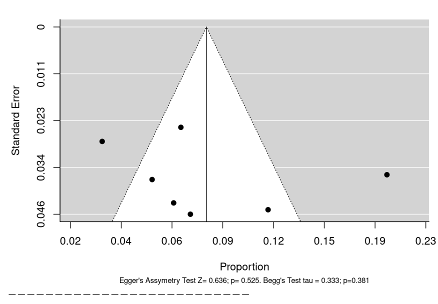

Suppl Figure 2.25.1 Forest Plot for Attention deficit hyperactivity disorder

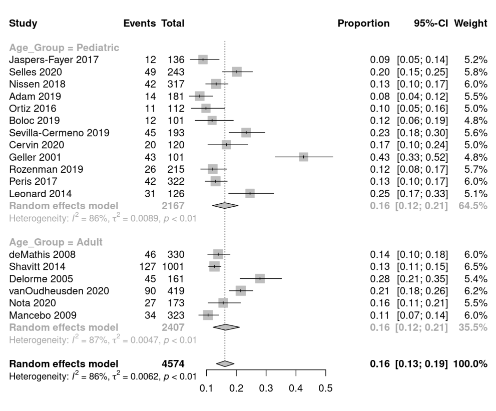

Suppl Figure 2.25.2 Funnel Plot for Attention deficit hyperactivity disorder

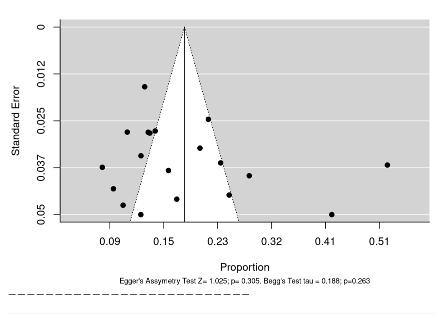

Suppl Figure 2.26.1 Forest Plot for Oppositional defiant disorder

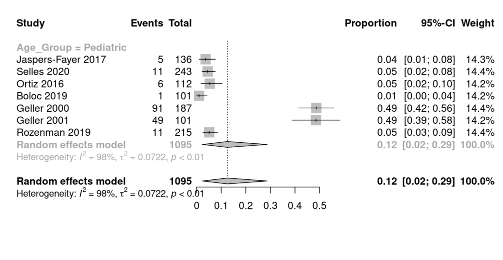

Suppl Figure 2.26.2 Funnel Plot for Oppositional defiant disorder

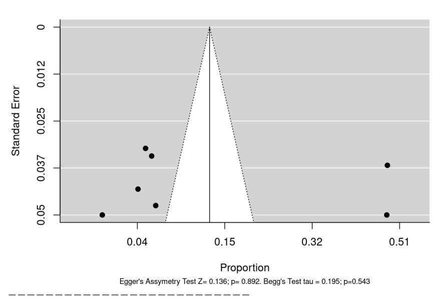

Suppl Figure 2.27.1 Forest Plot for Any personality disorder

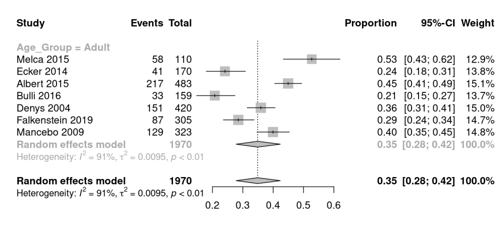

Suppl Figure 2.27.2 Funnel Plot for Any personality disorder

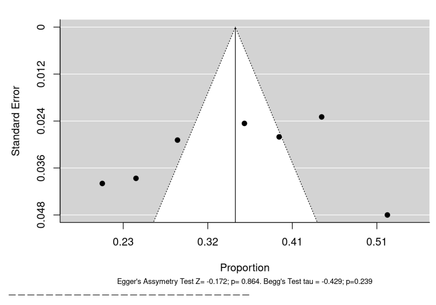

Suppl Figure 2.28.1 Forest Plot for Obsessive compulsive (Anankastic) personality disorder

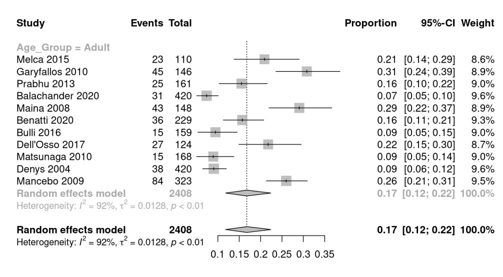

Suppl Figure 2.28.2 Funnel Plot for Obsessive compulsive (Anankastic) personality disorder

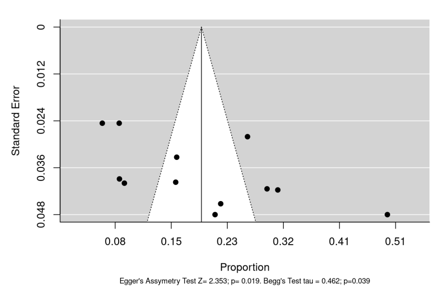

Suppl Figure 2.29.1 Forest Plot for Schizoid personality disorder

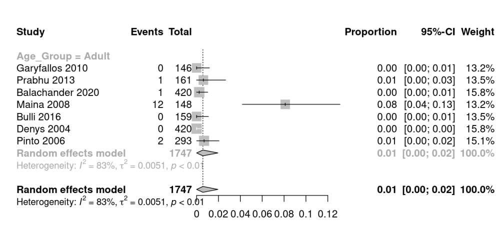

Suppl Figure 2.29.2 Funnel Plot for Schizoid personality disorder

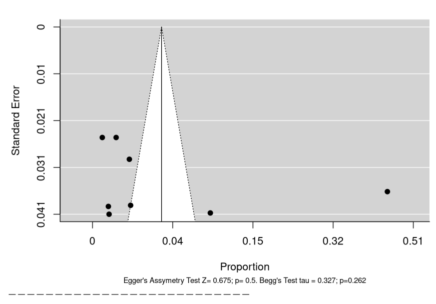

Suppl Figure 2.30.1 Forest Plot for Schizotypal personality disorder

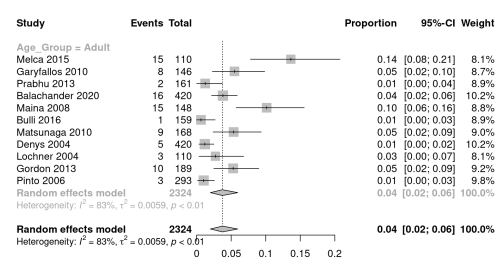

Suppl Figure 2.30.2 Funnel Plot for Schizotypal personality disorder

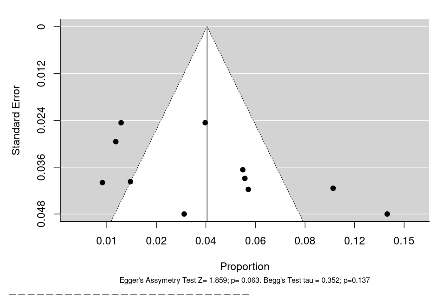

Suppl Figure 2.31.1 Forest Plot for Borderline personality disorder

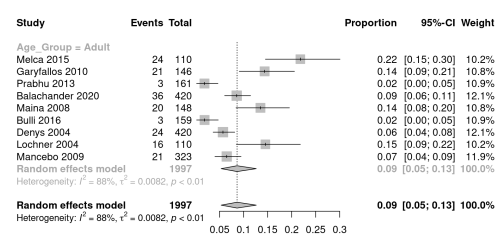

Suppl Figure 2.31.2 Funnel Plot for Borderline personality disorder

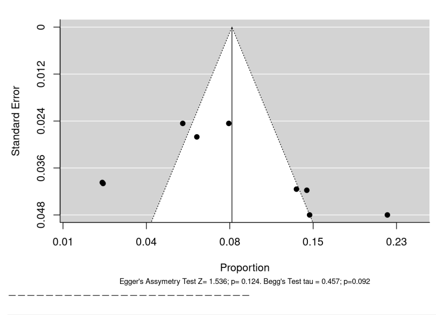

Suppl Figure 2.32.1 Forest Plot for Histrionic personality disorder

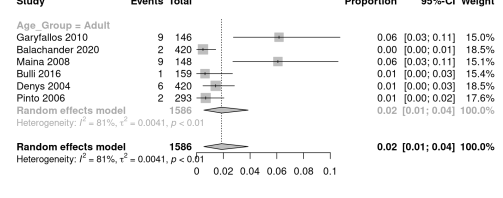

Suppl Figure 2.32.2 Funnel Plot for Histrionic personality disorder

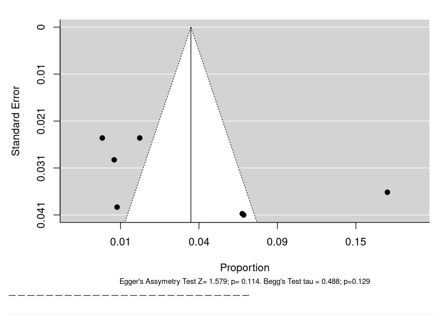

Suppl Figure 2.33.1 Forest Plot for Antisocial personality disorder

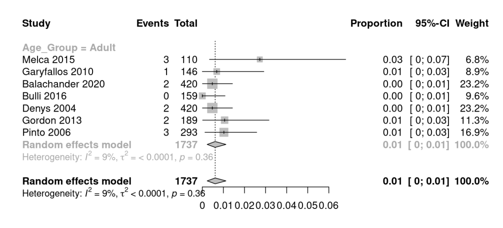

Suppl Figure 2.33.2 Funnel Plot for Antisocial personality disorder

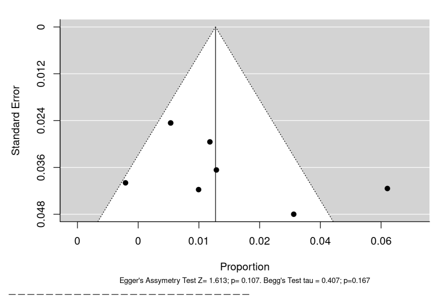

Suppl Figure 2.34.1 Forest Plot for Narcissistic personality disorder

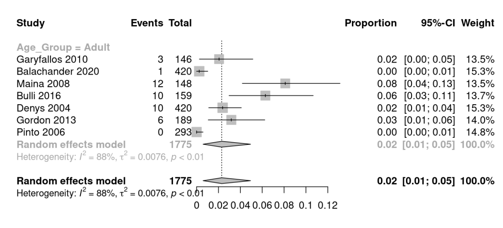

Suppl Figure 2.34.2 Funnel Plot for Narcissistic personality disorder

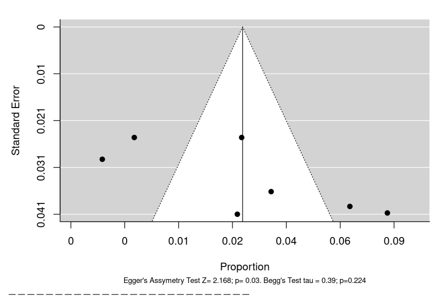

Suppl Figure 2.35.1 Forest Plot for Dependent personality disorder

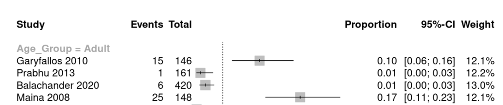

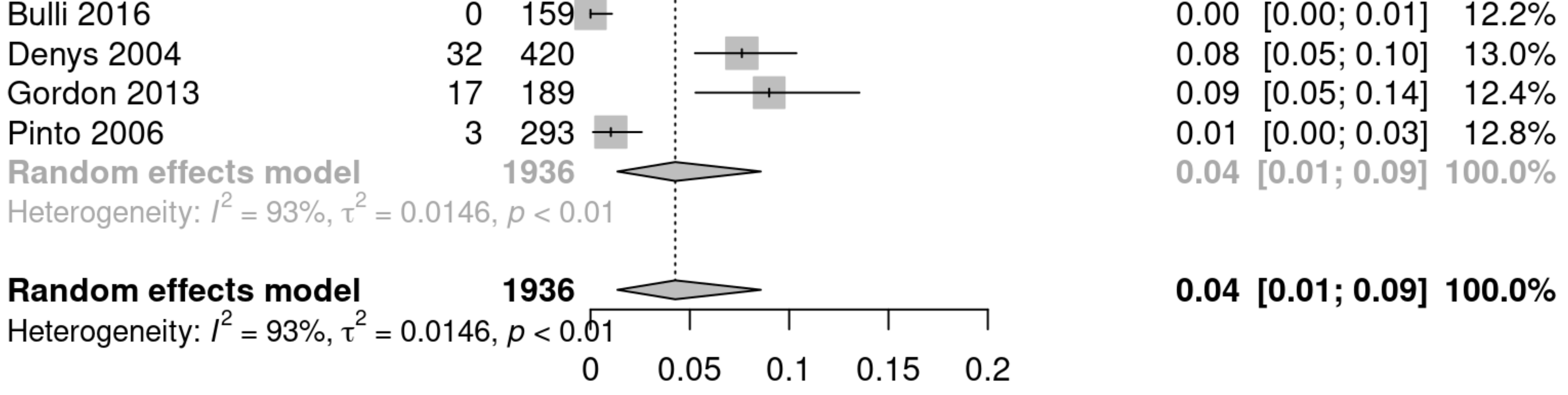

Suppl Figure 2.35.2 Funnel Plot for Dependent personality disorder

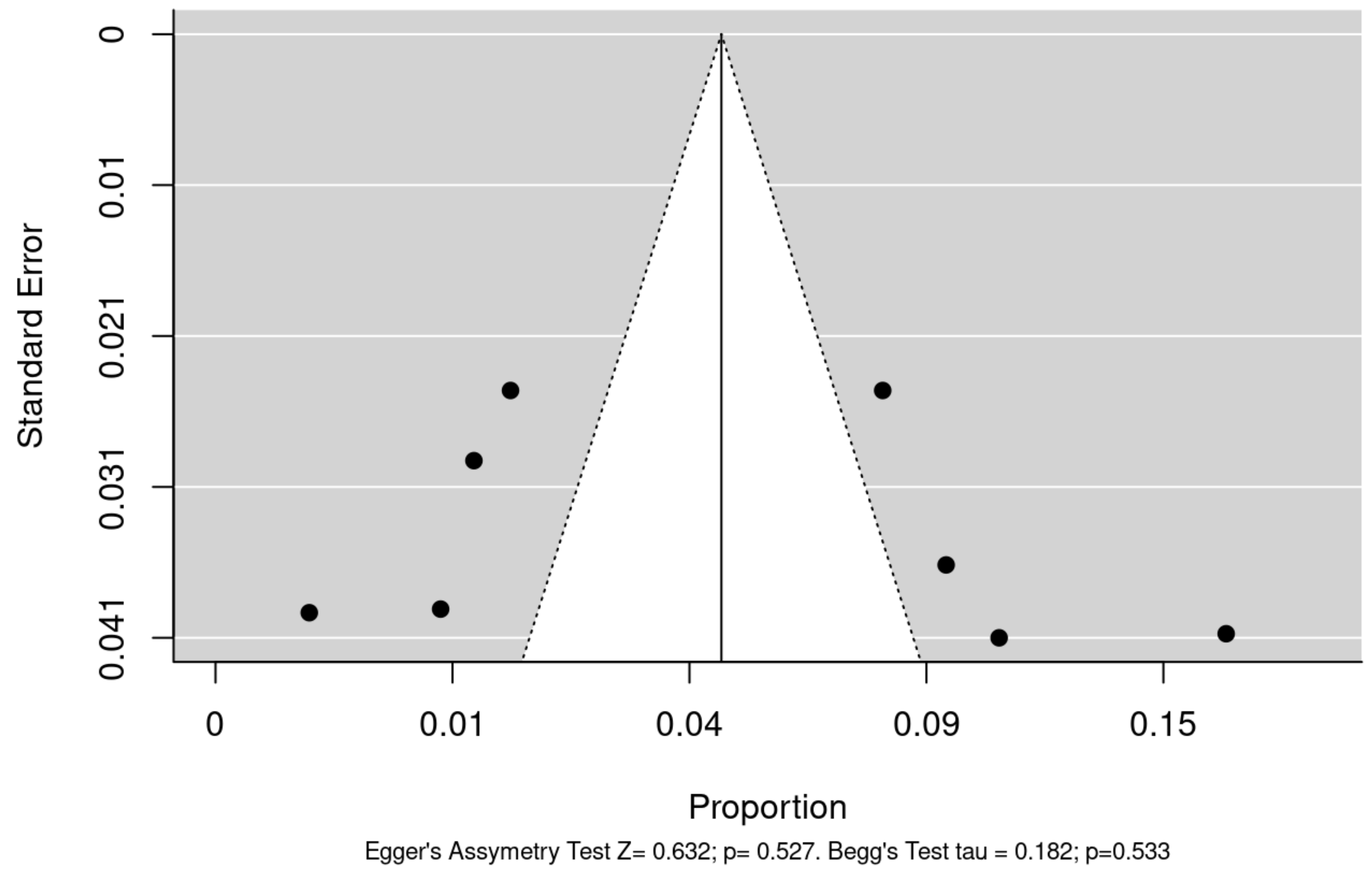

Suppl Figure 2.36.1 Forest Plot for Anxious-avoidant personality disorder

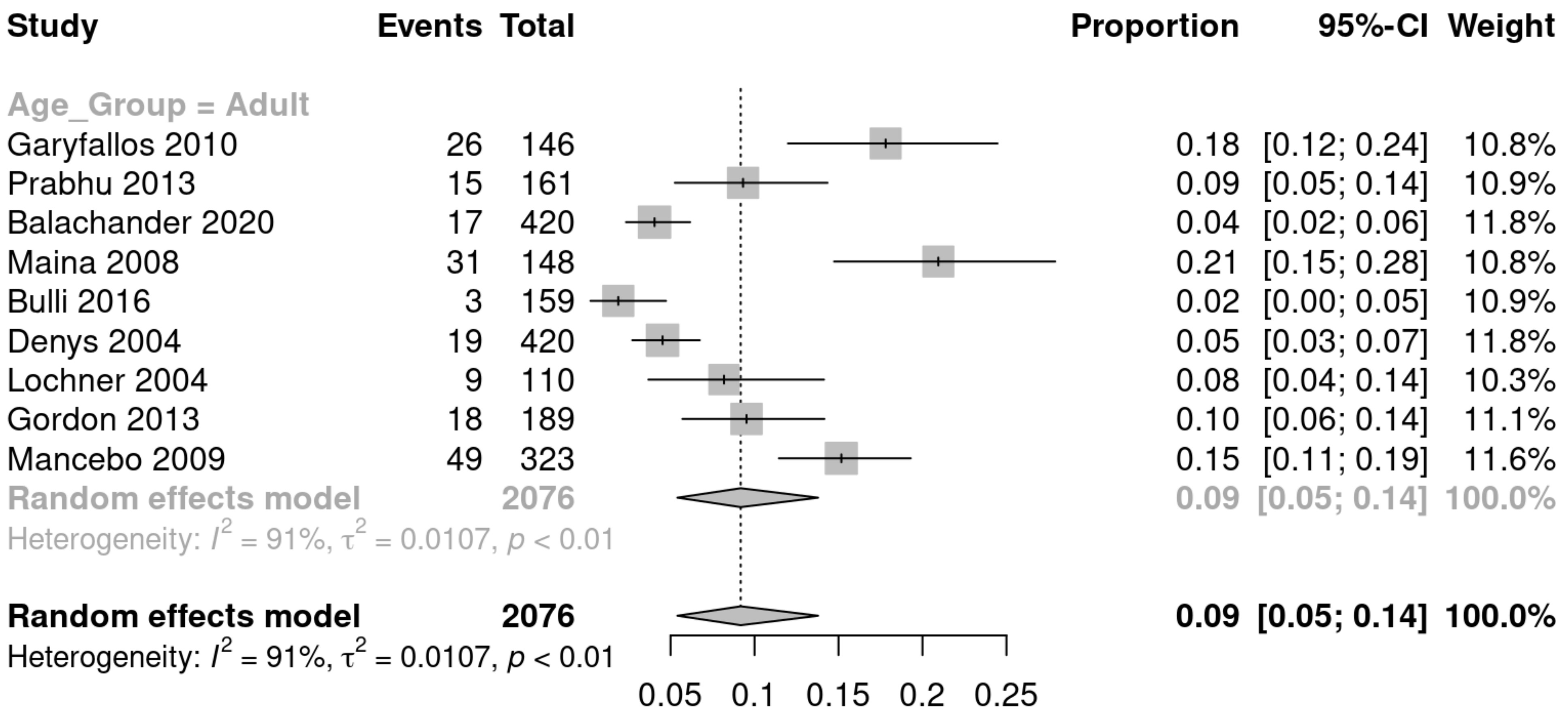

Suppl Figure 2.36.2 Funnel Plot for Anxious-avoidant personality disorder

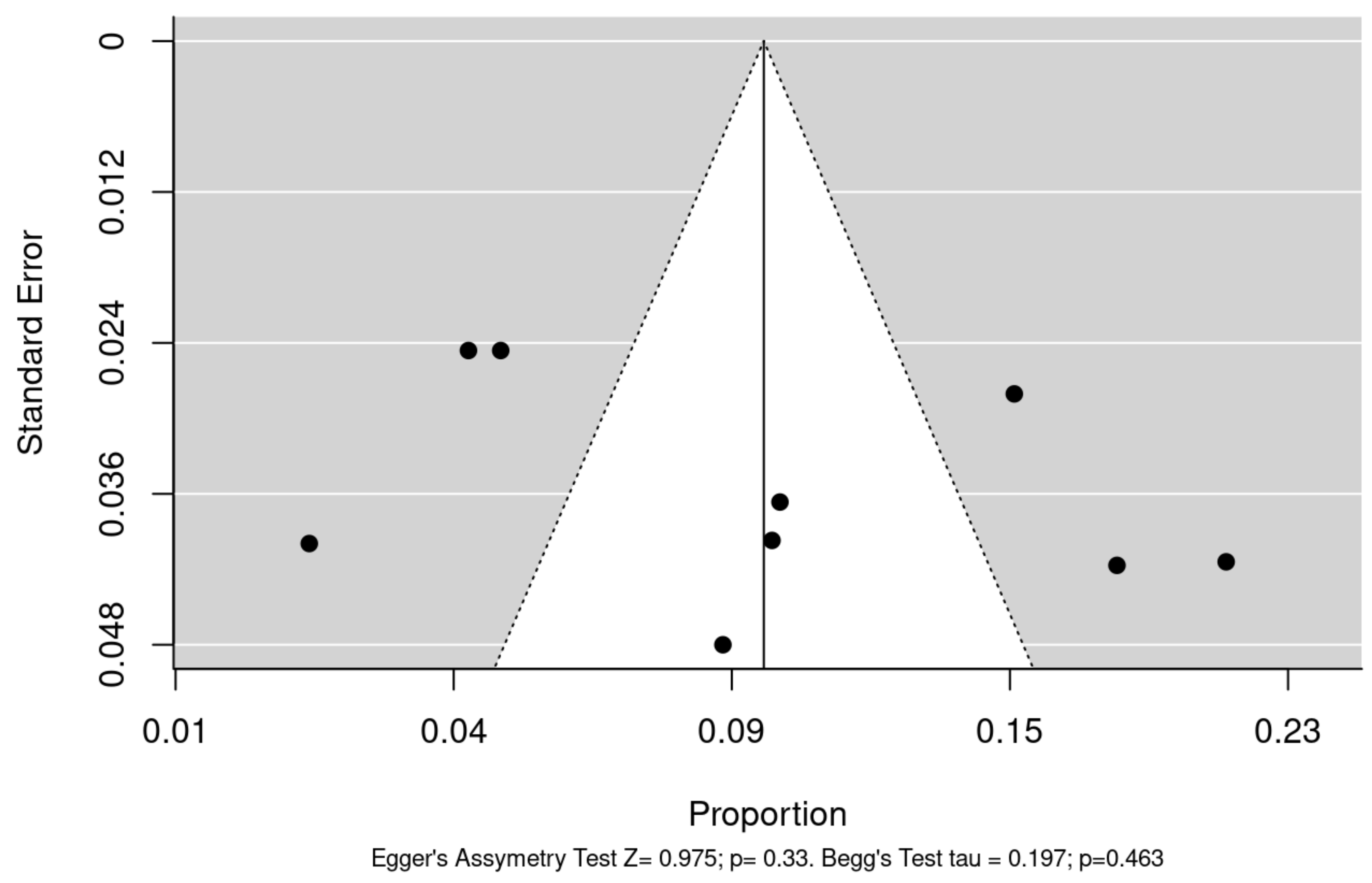

Supplement: Supplementary file 3 [file Data_Sheet_3.PDF]
