# Supplementary material for: Comorbidities in Obsessive-Compulsive Disorder Across the Lifespan: A Systematic Review and Meta-Analysis
Source: Front Psychiatry. 2021 Nov 11;12:703701. doi: 10.3389/fpsyt.2021.703701 (PMC8631971; doi:10.3389/fpsyt.2021.703701)

OCD Comorbidity Meta-analysis: Across the Lifespan

Sharma E et al.

26/07/2021

Meta-regression plots for each disorder

Each circle represents a study sample. Blue indicates sample drawn from an adult OCD study; red indicates a pediatric OCD sample.

The size of the circle is proportional to the sample size of that study

The solid line is the meta-regression line,the dotted lines are the 95% confidence intervals

The QM statistic (& its p-value) is the statistical test for heterogeneity explained by the meta-regressor

---

Suppl Figure 3.1 Meta-regression plots for Any psychiatric comorbidity

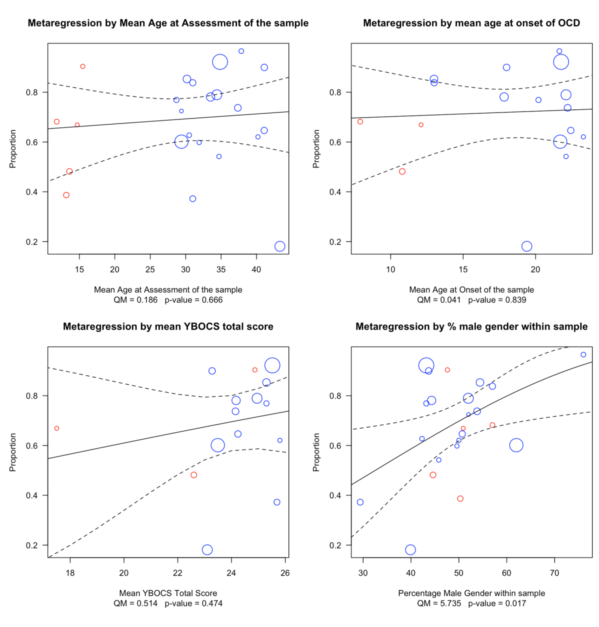

Suppl Figure 3.2 Meta-regression plots for Any mood disorder

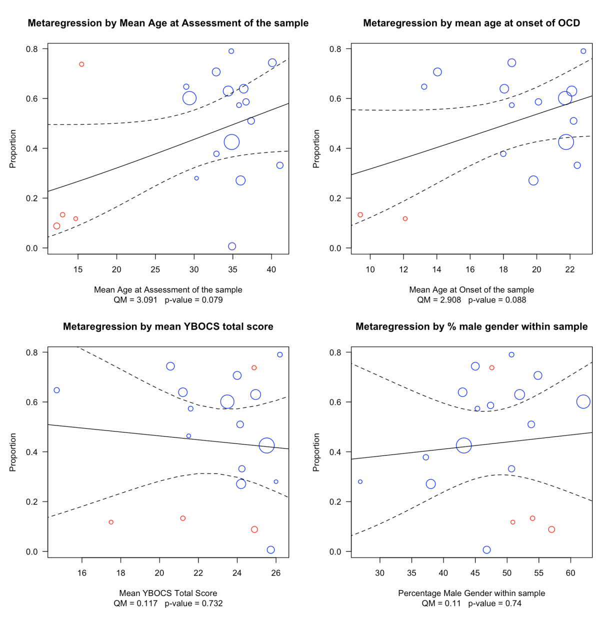

Suppl Figure 3.3 Meta-regression plots for Major Depressive Disorder

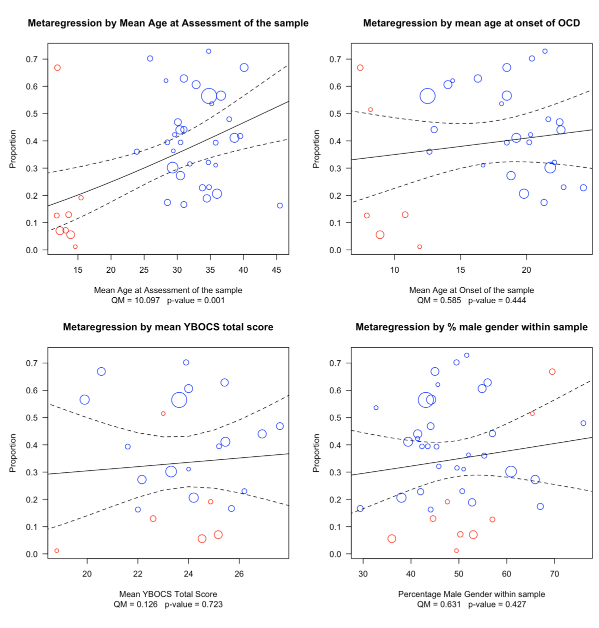

Suppl Figure 3.4 Meta-regression plots for Dysthymia

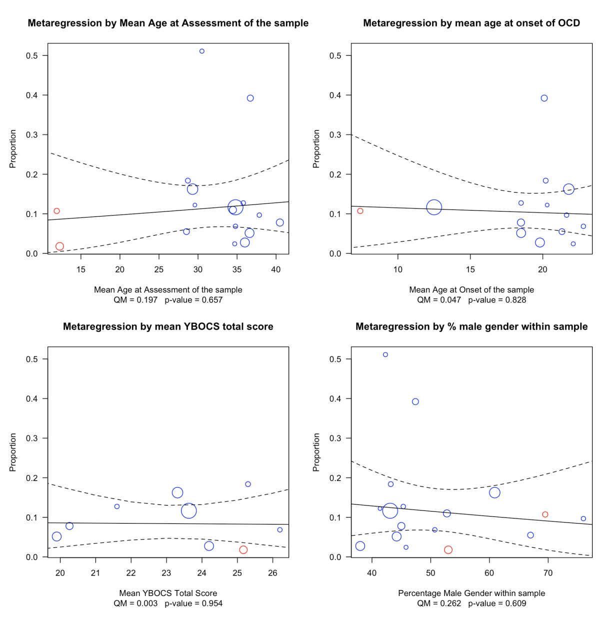

Suppl Figure 3.5 Meta-regression plots for Bipolar Disorder

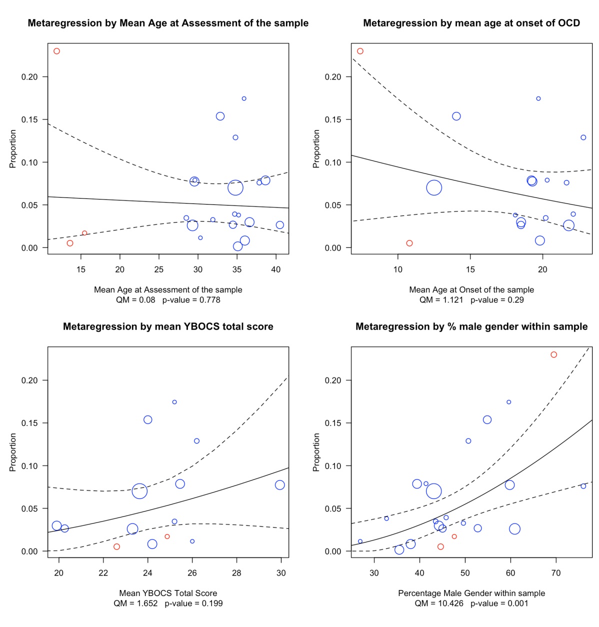

Suppl Figure 3.6 Meta-regression plots for Schizophrenia/Any psychotic disorder

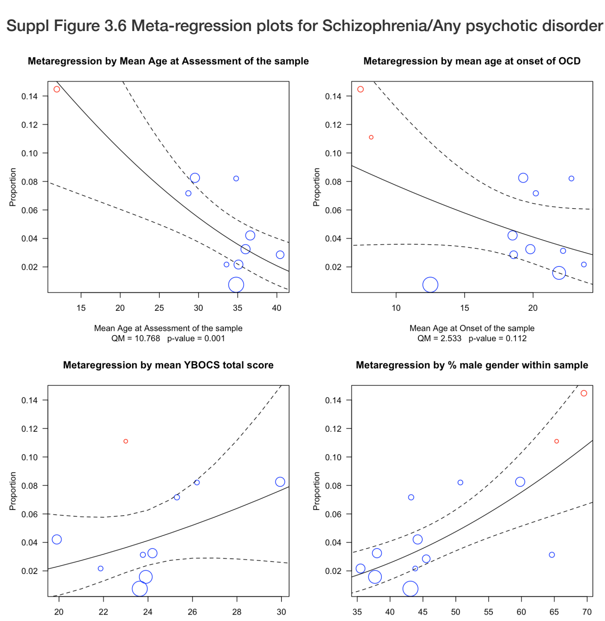

Suppl Figure 3.7 Meta-regression plots for Any anxiety disorder

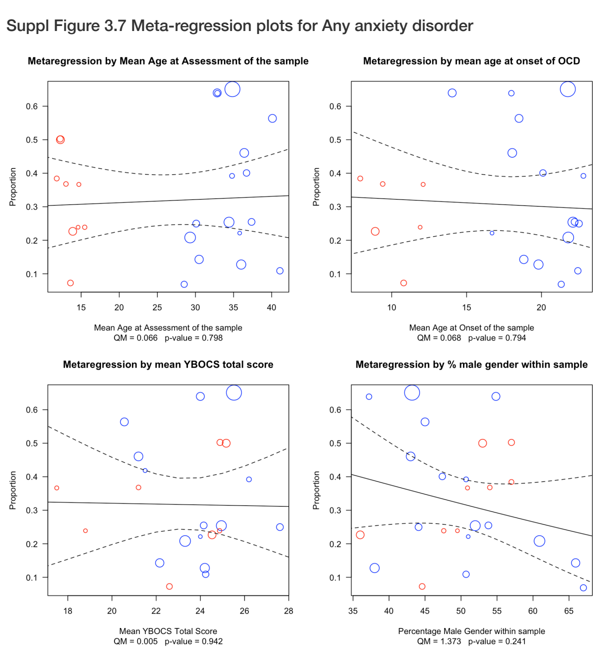

Suppl Figure 3.8 Meta-regression plots for Generalised Anxiety Disorder

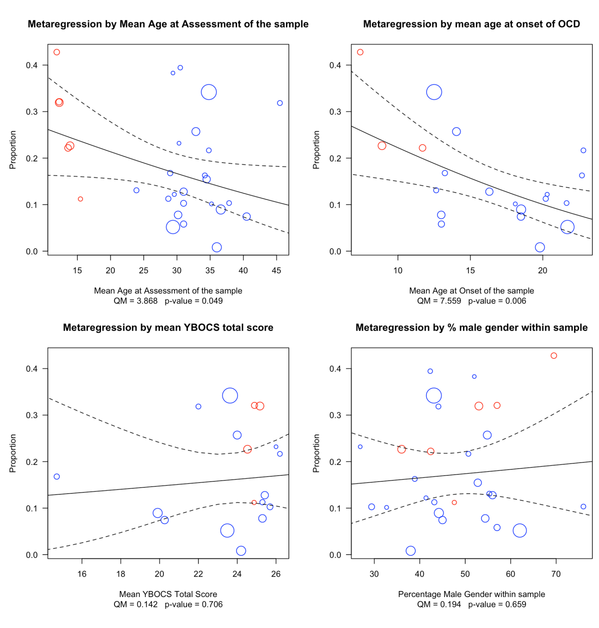

Suppl Figure 3.9 Meta-regression plots for Social Anxiety Disorder

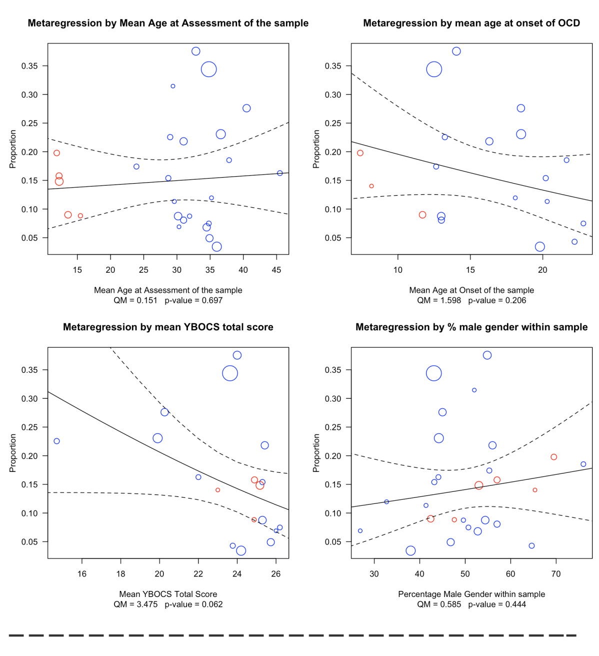

Suppl Figure 3.10 Meta-regression plots for Panic Disorder

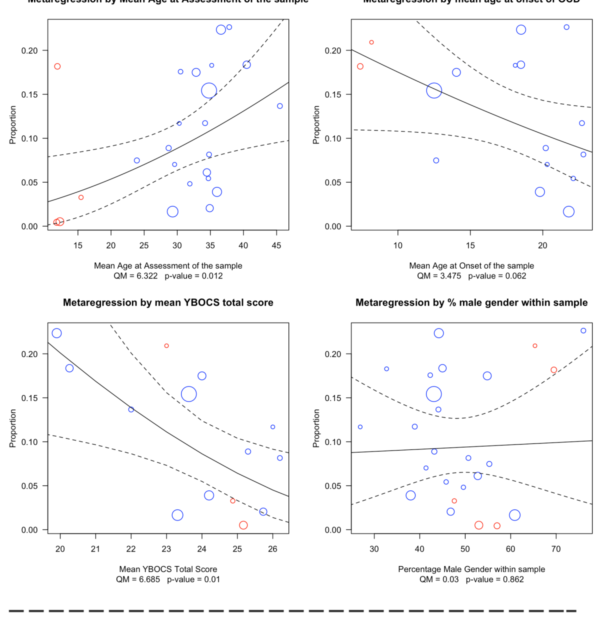

Suppl Figure 3.11 Meta-regression plots for Agoraphobia

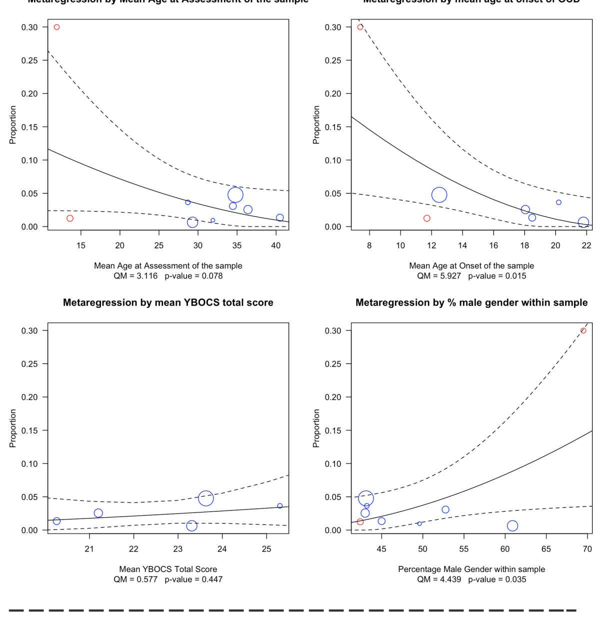

Suppl Figure 3.12 Meta-regression plots for Simple/Specific Phobia

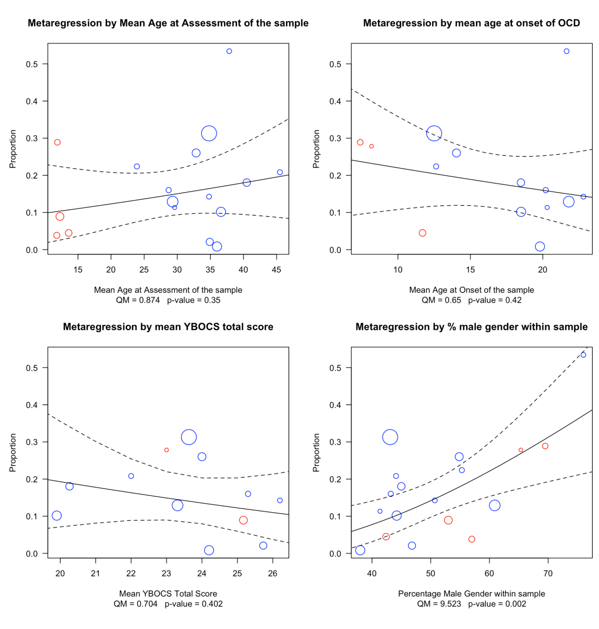

Suppl Figure 3.13 Meta-regression plots for Somatic symptom disorder/Somatoform/Somatization disorder

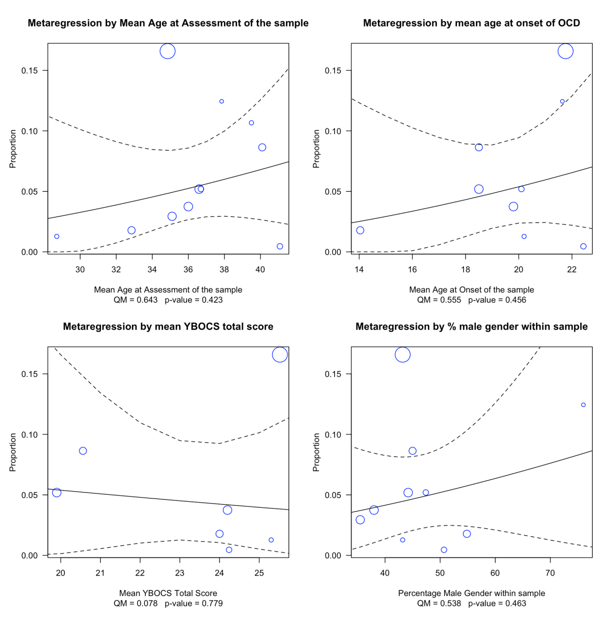

Suppl Figure 3.14 Meta-regression plots for Hypochondriasis/Illness anxiety disorder

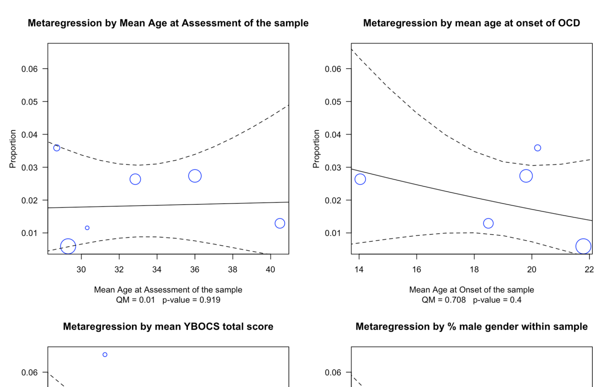

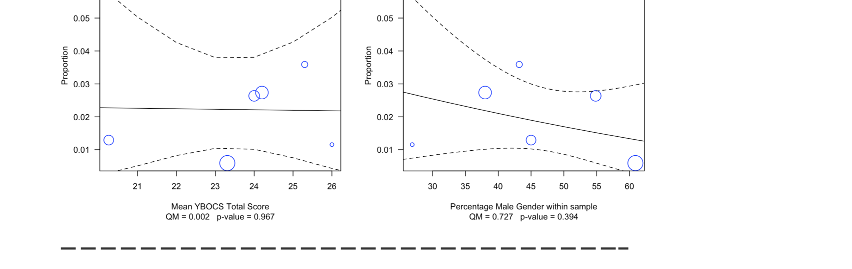

Suppl Figure 3.15 Meta-regression plots for Post-traumatic stress disorder (PTSD)

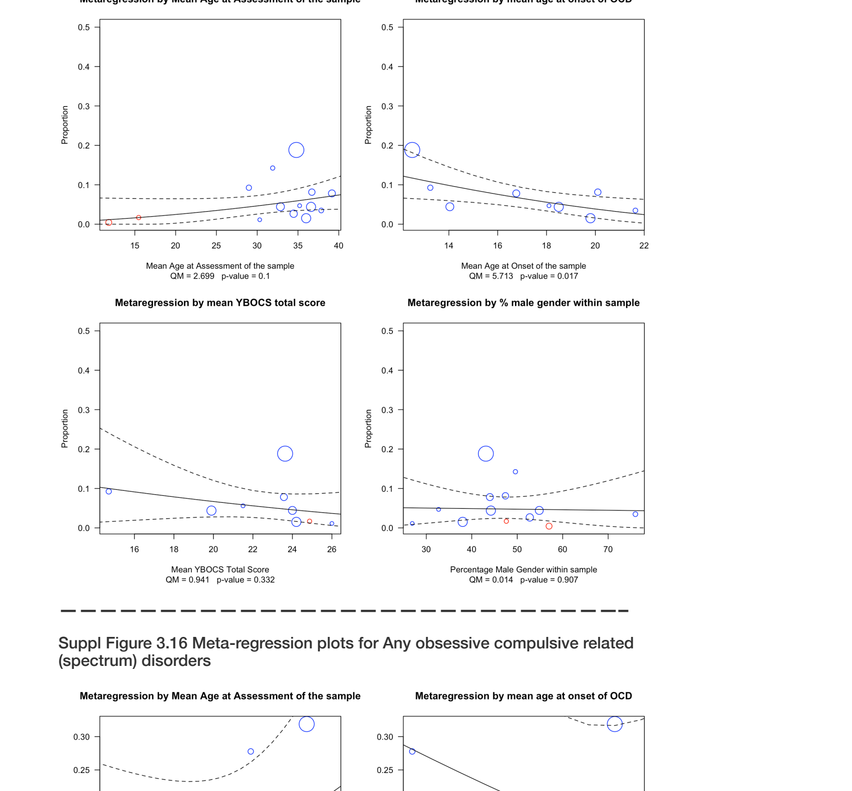

Suppl Figure 3.16 Meta-regression plots for Any obsessive compulsive related (spectrum) disorders

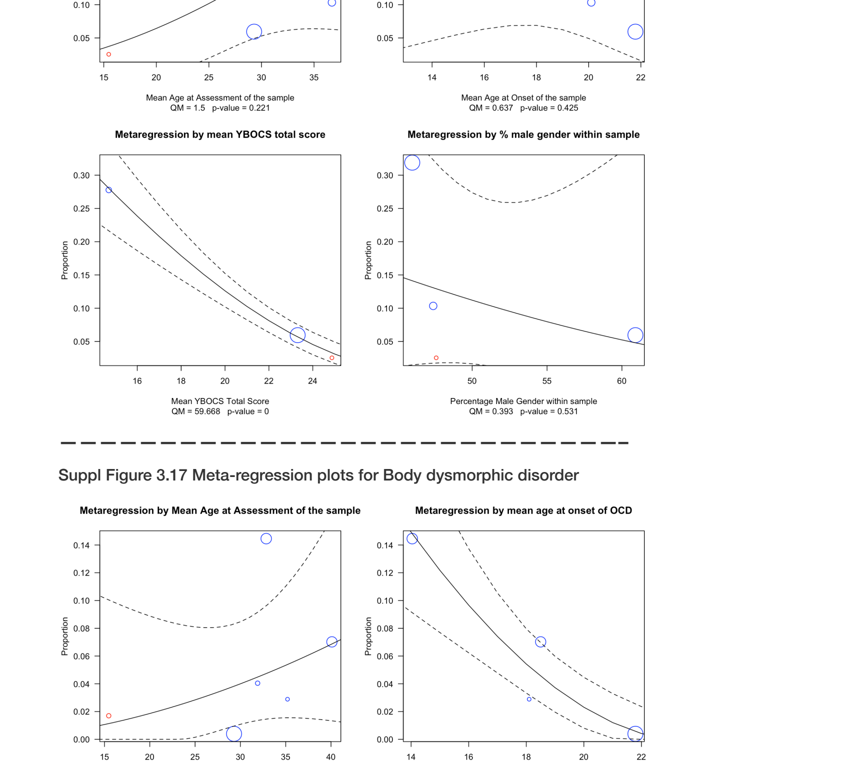

Suppl Figure 3.17 Meta-regression plots for Body dysmorphic disorder

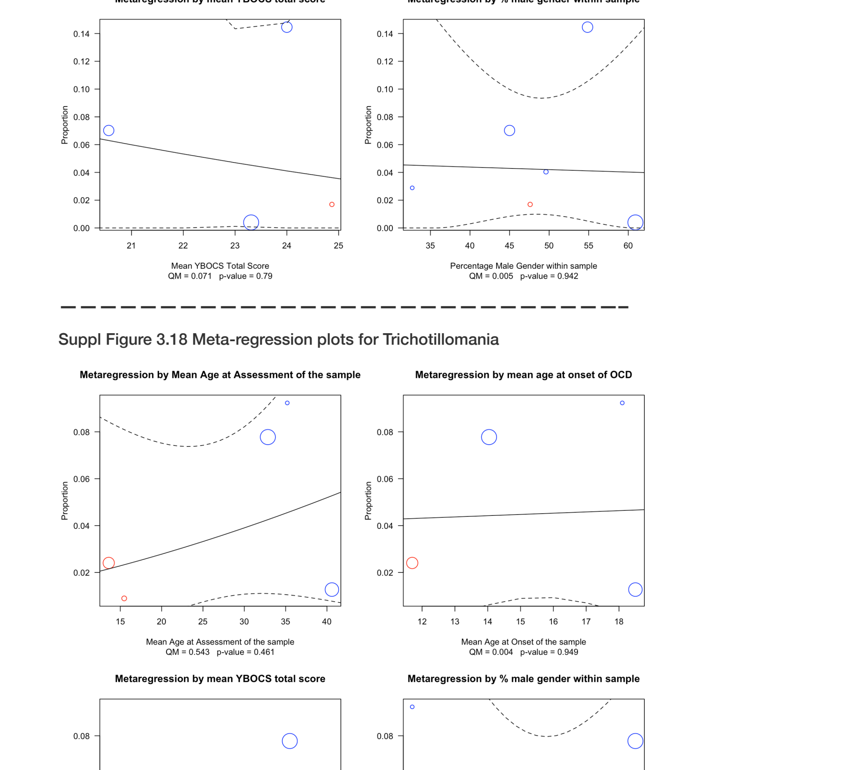

Suppl Figure 3.18 Meta-regression plots for Trichotillomania

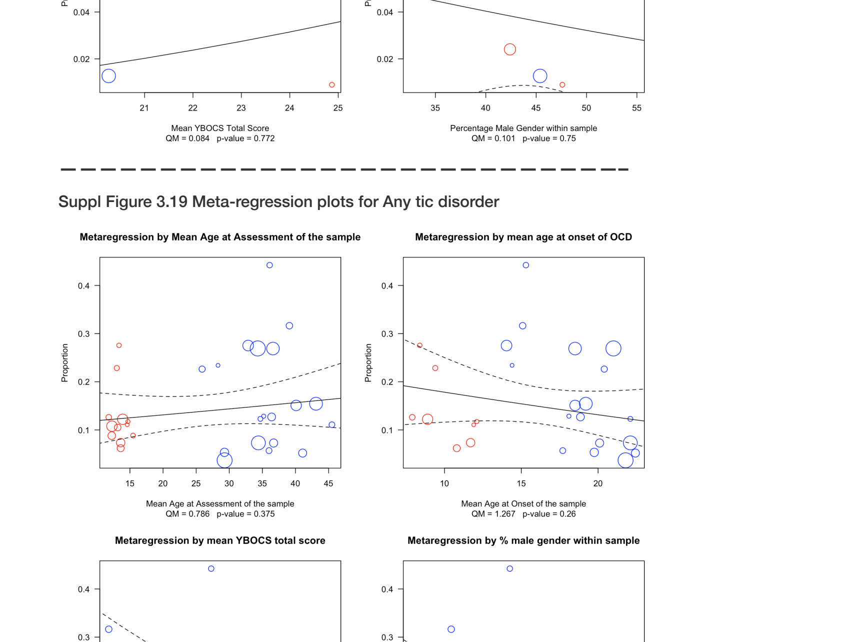

Suppl Figure 3.19 Meta-regression plots for Any tic disorder

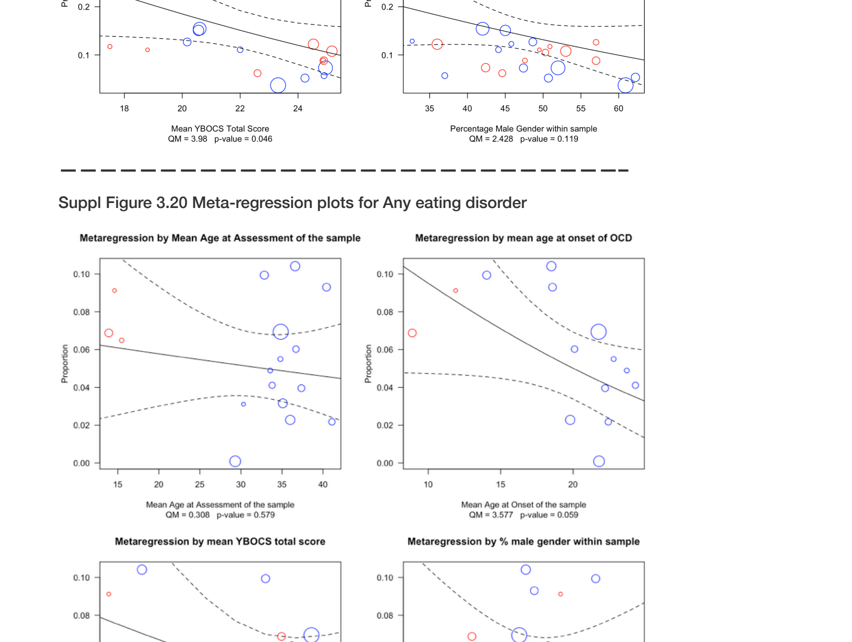

Suppl Figure 3.20 Meta-regression plots for Any eating disorder

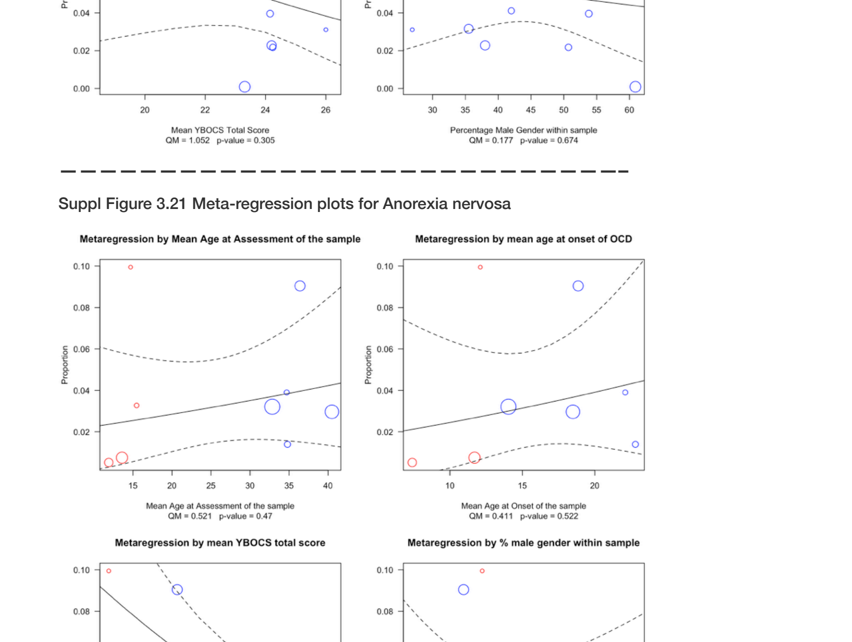

Suppl Figure 3.21 Meta-regression plots for Anorexia nervosa

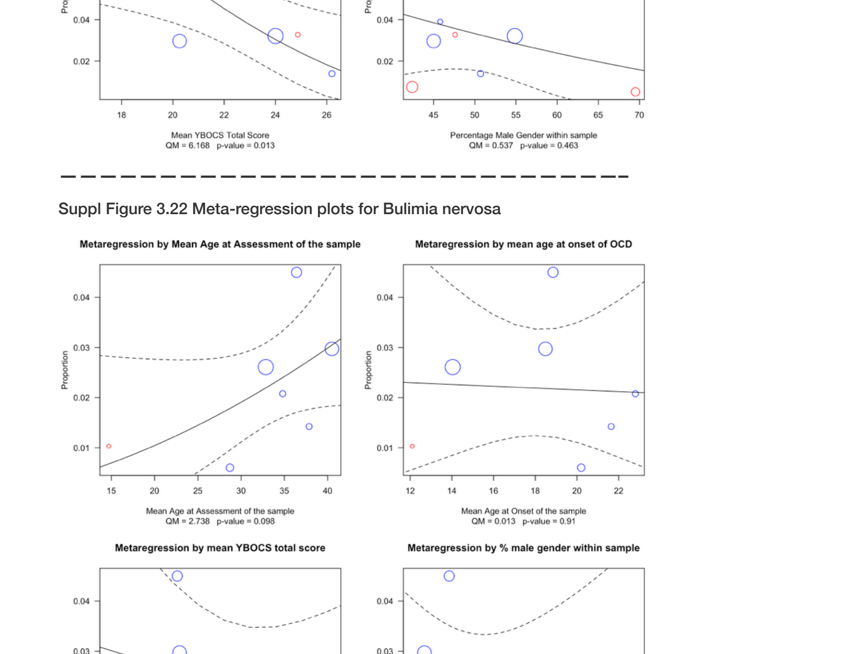

Suppl Figure 3.22 Meta-regression plots for Bulimia nervosa

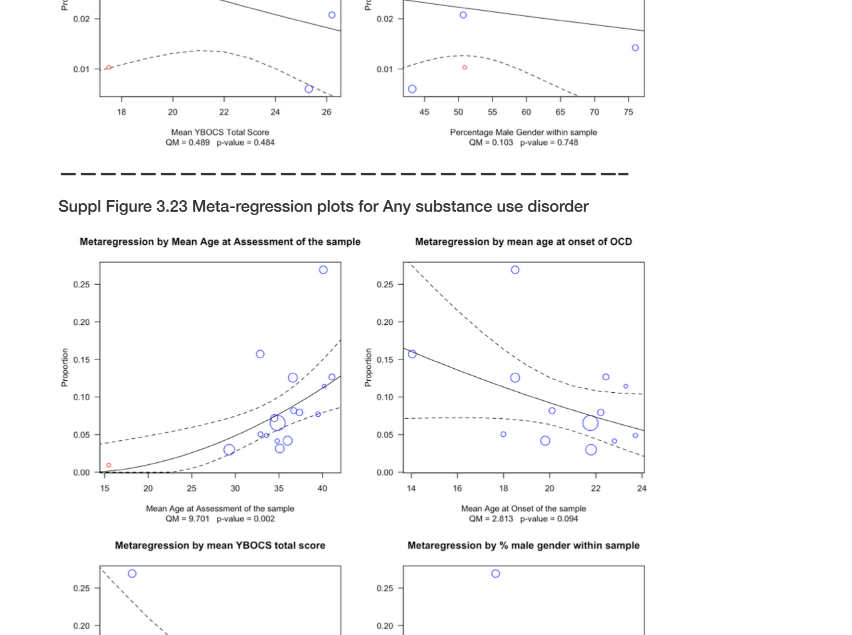

Suppl Figure 3.23 Meta-regression plots for Any substance use disorder

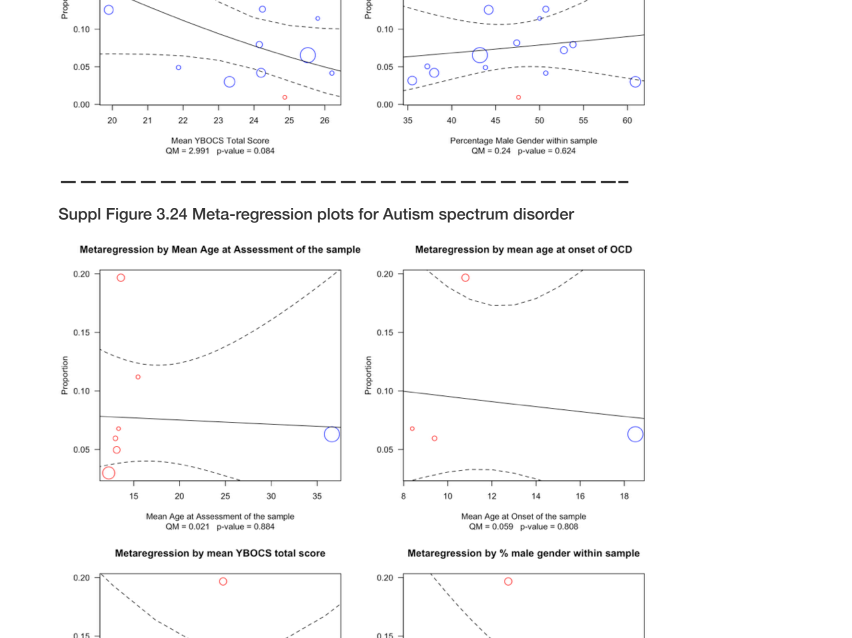

Suppl Figure 3.24 Meta-regression plots for Autism spectrum disorder

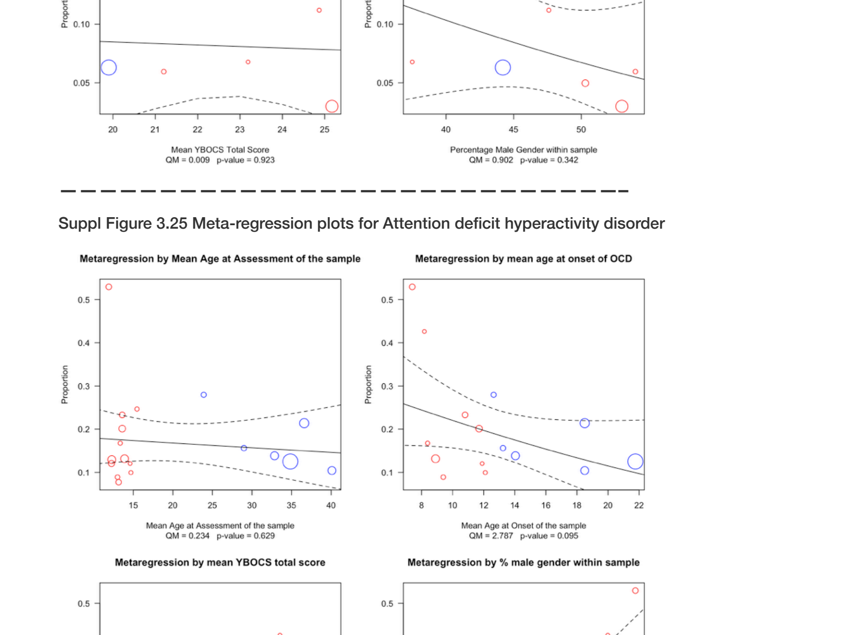

Suppl Figure 3.25 Meta-regression plots for Attention deficit hyperactivity disorder

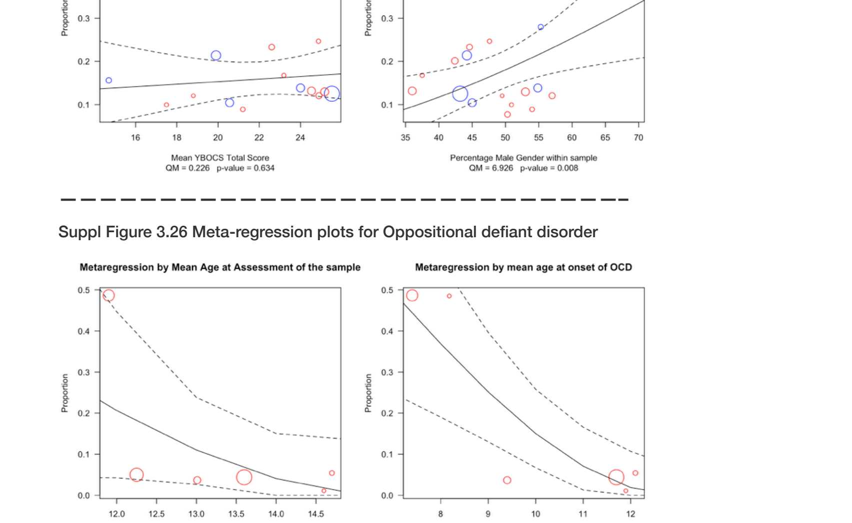

Suppl Figure 3.26 Meta-regression plots for Oppositional defiant disorder

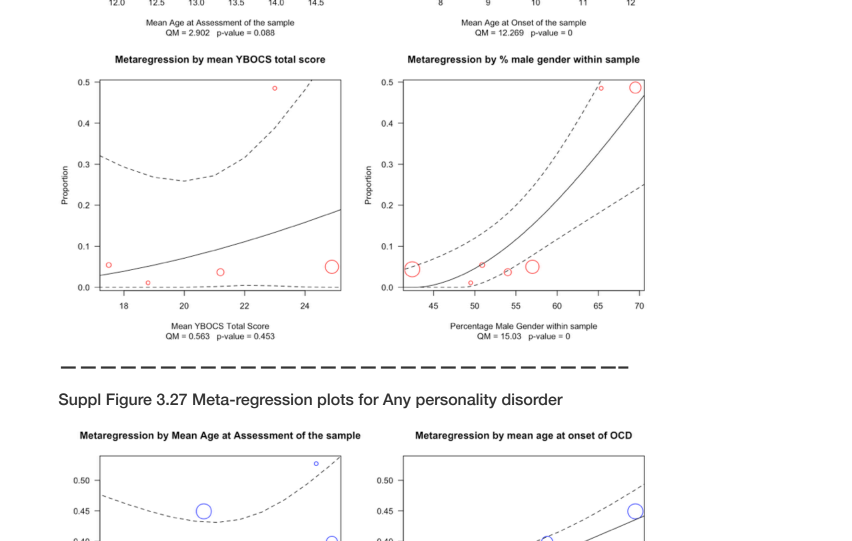

Suppl Figure 3.27 Meta-regression plots for Any personality disorder

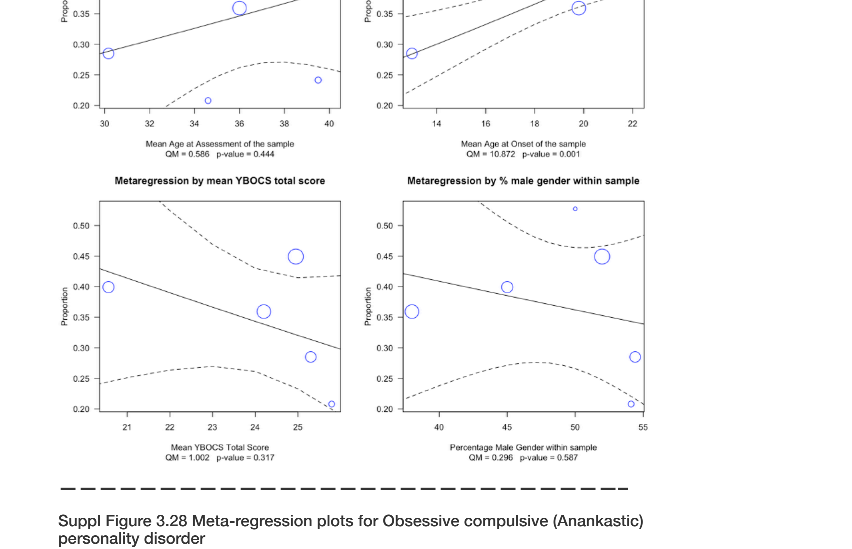

Suppl Figure 3.28 Meta-regression plots for Obsessive compulsive (Anankastic) personality disorder

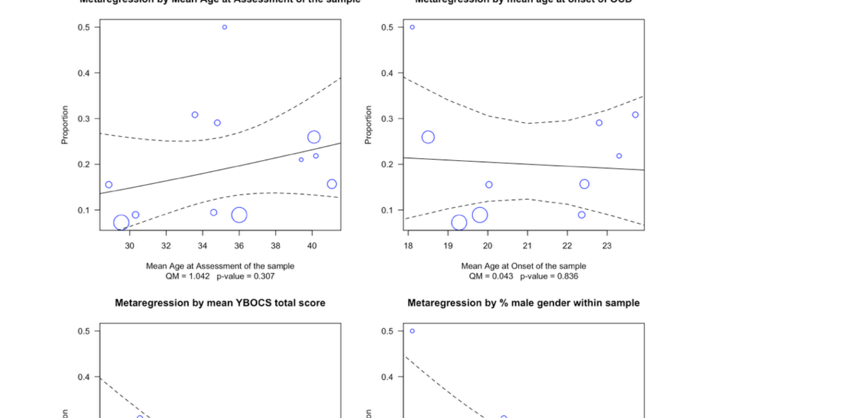

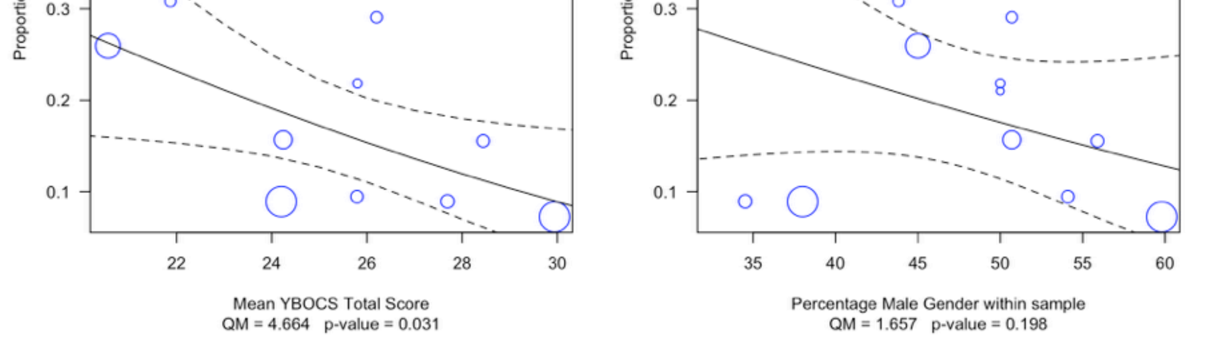

Suppl Figure 3.29 Meta-regression plots for Schizoid personality disorder

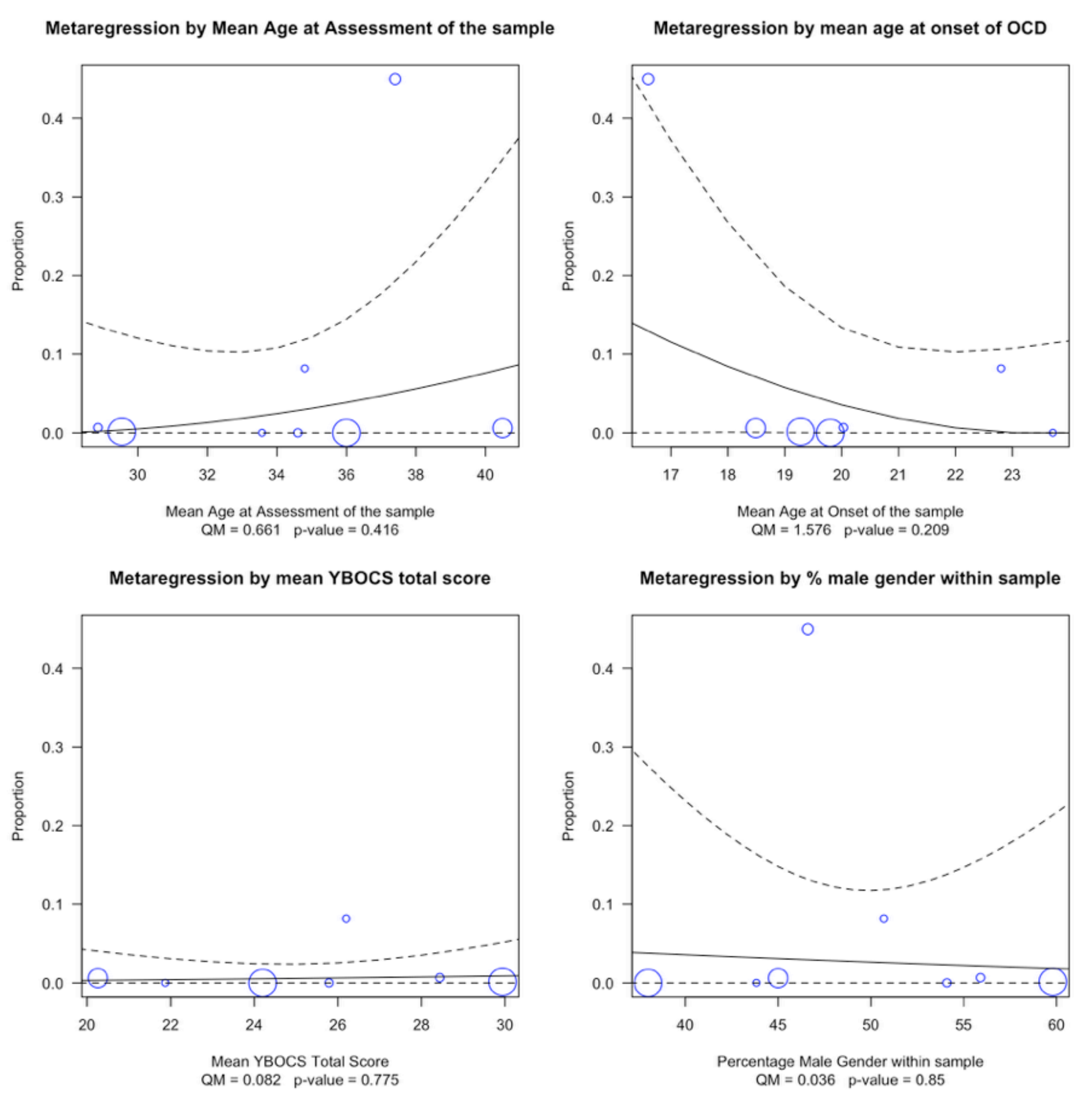

Suppl Figure 3.30 Meta-regression plots for Schizotypal personality disorder

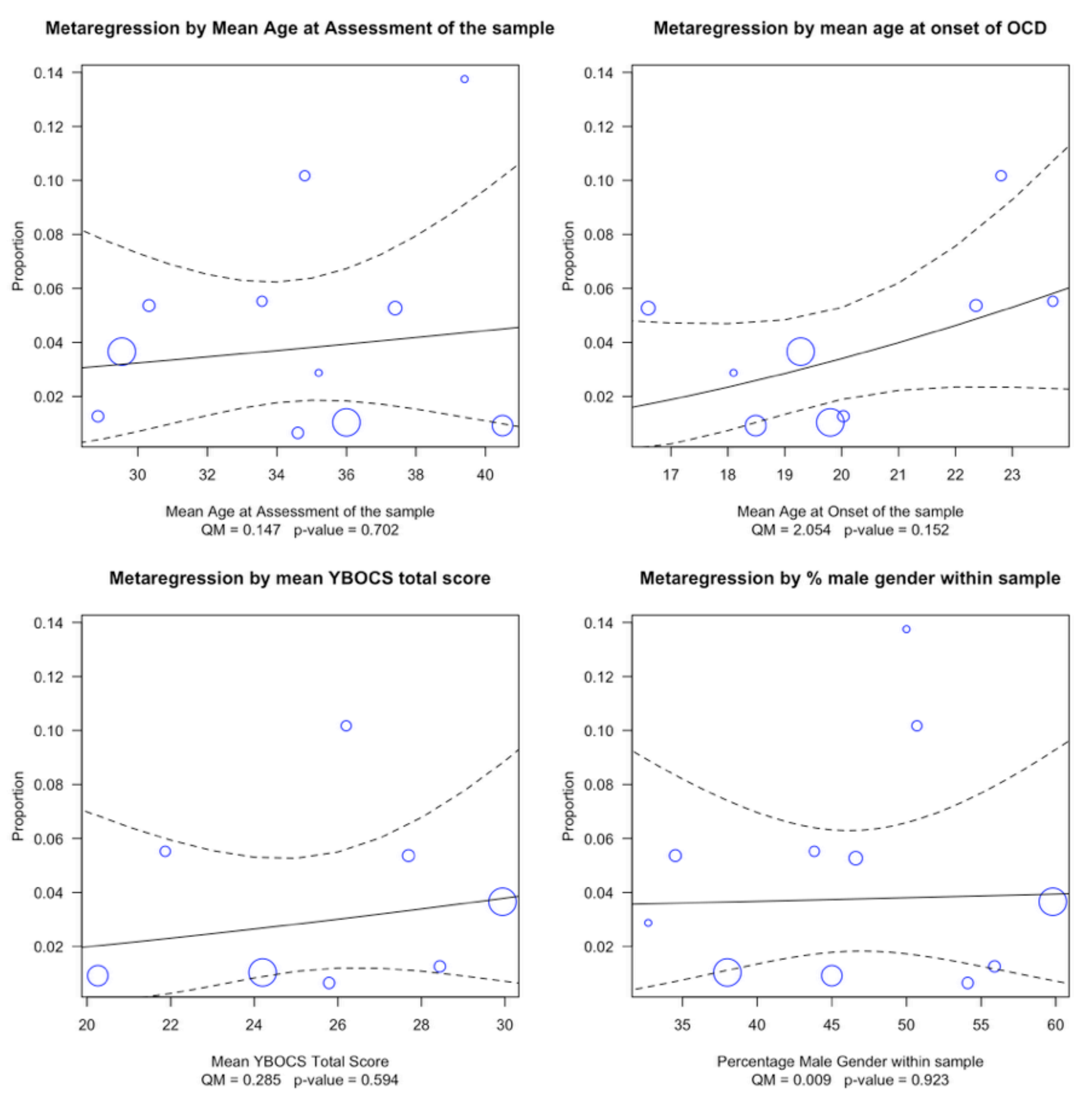

Suppl Figure 3.31 Meta-regression plots for Borderline personality disorder

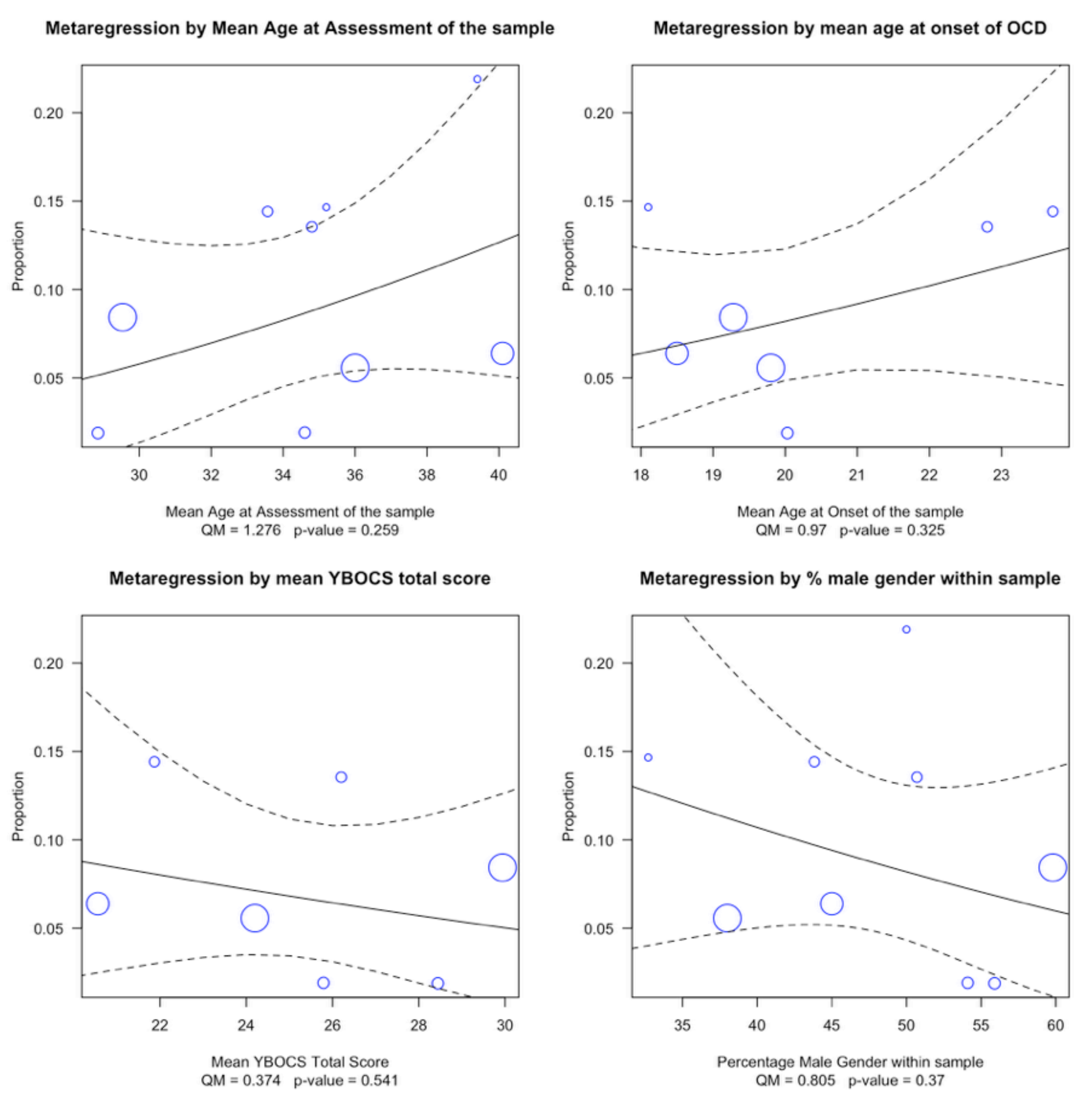

Suppl Figure 3.32 Meta-regression plots for Histrionic personality disorder

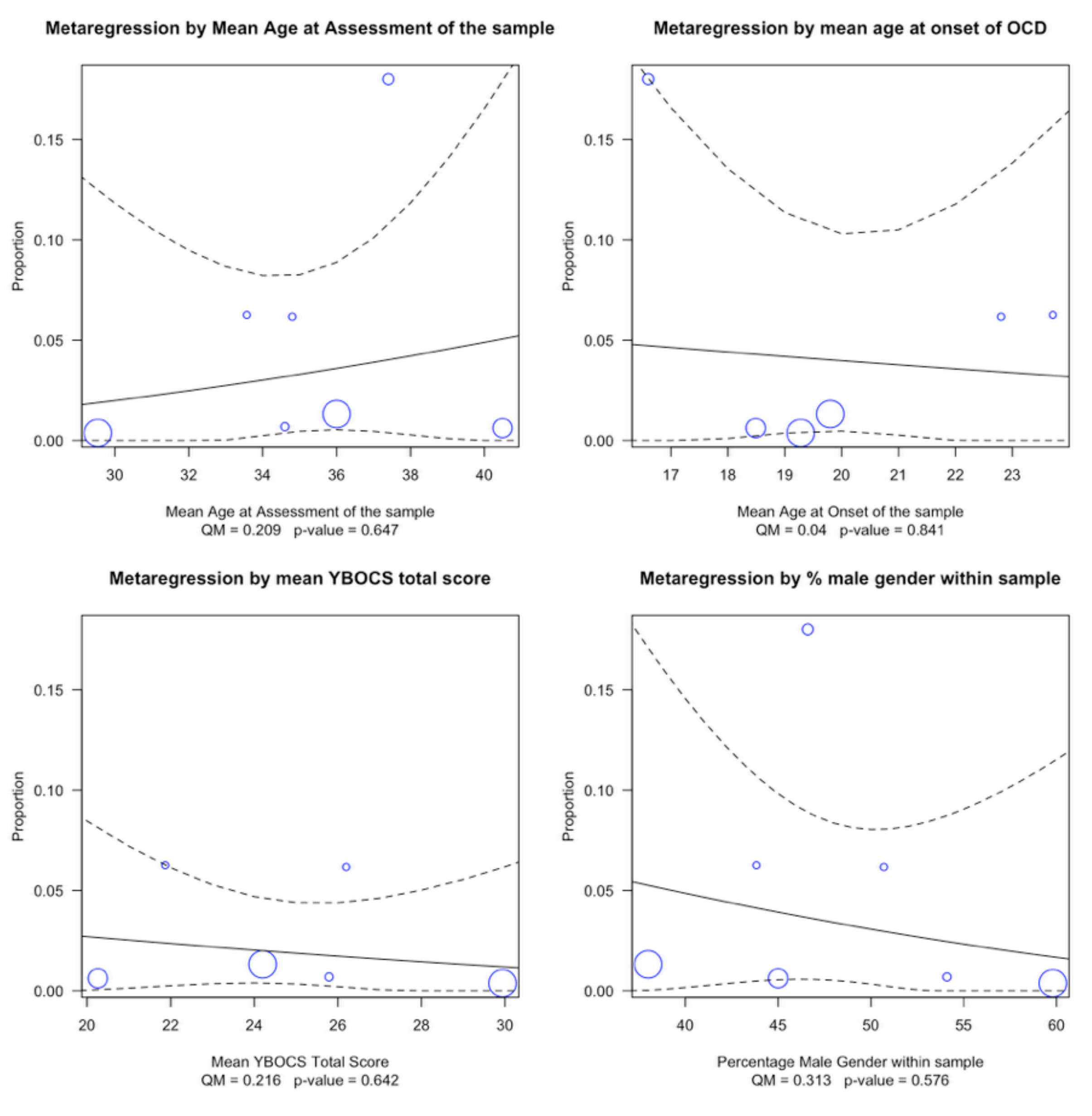

Suppl Figure 3.33 Meta-regression plots for Antisocial personality disorder

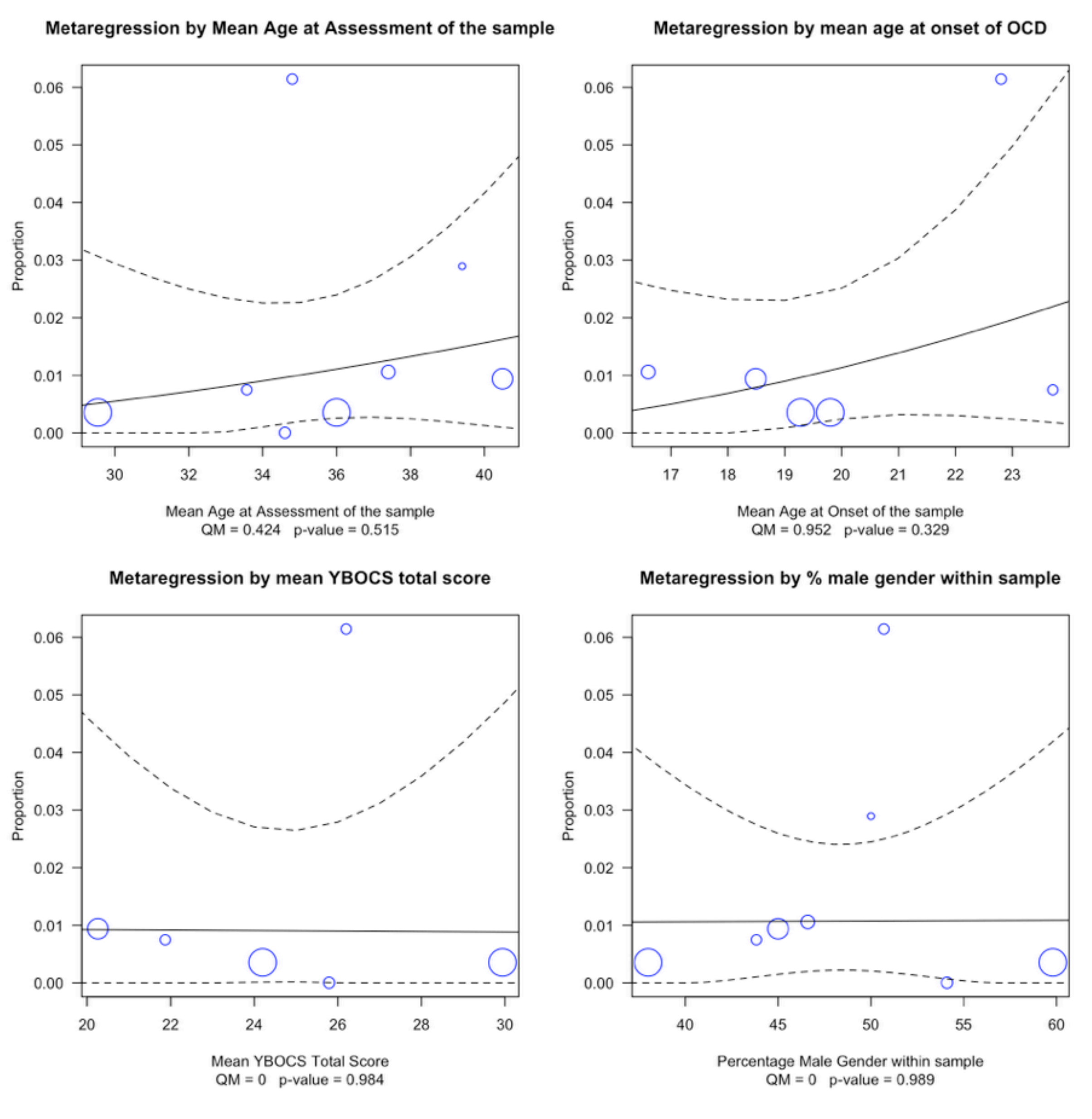

Suppl Figure 3.34 Meta-regression plots for Narcissistic personality disorder

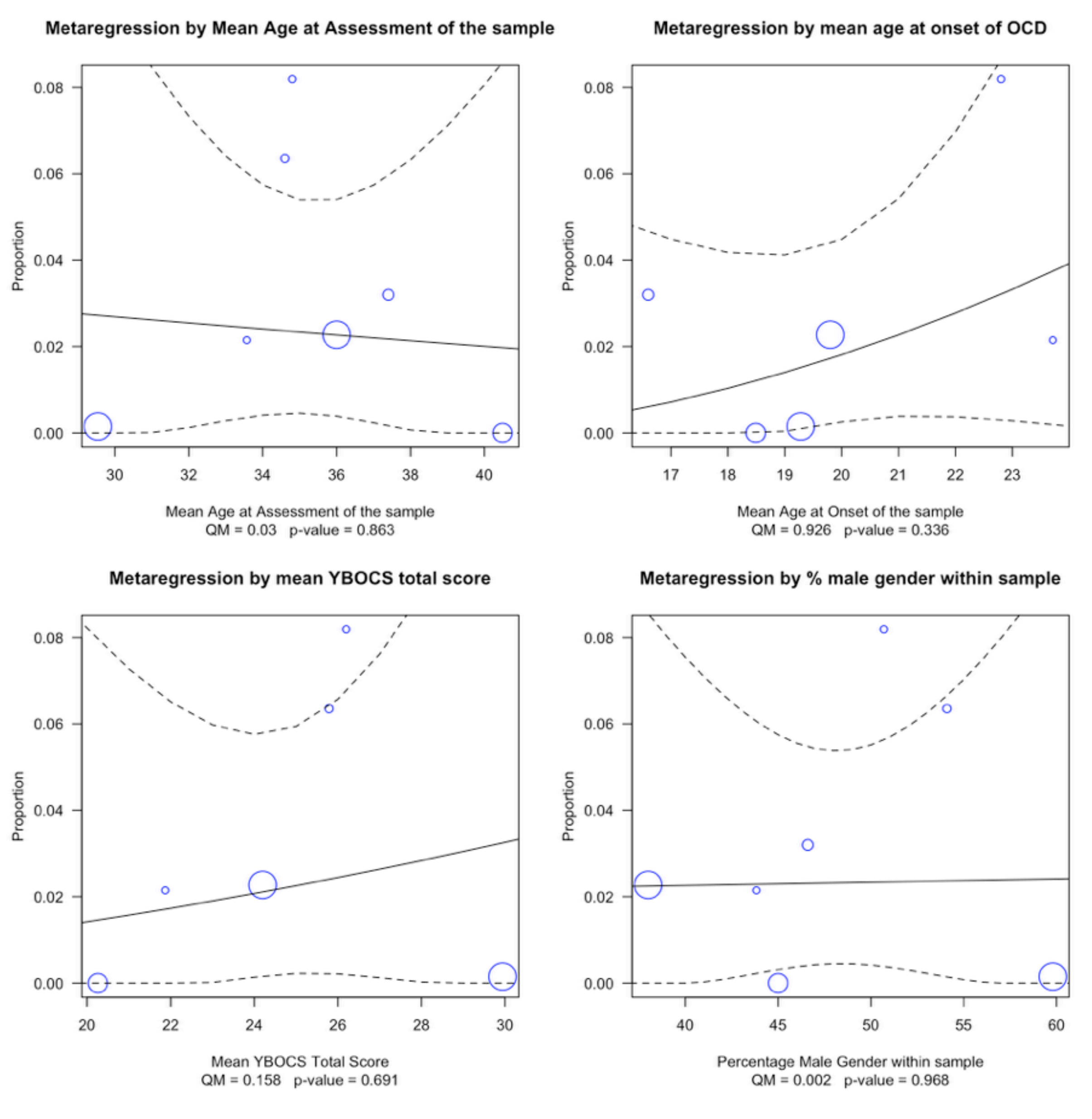

Suppl Figure 3.35 Meta-regression plots for Dependent personality disorder

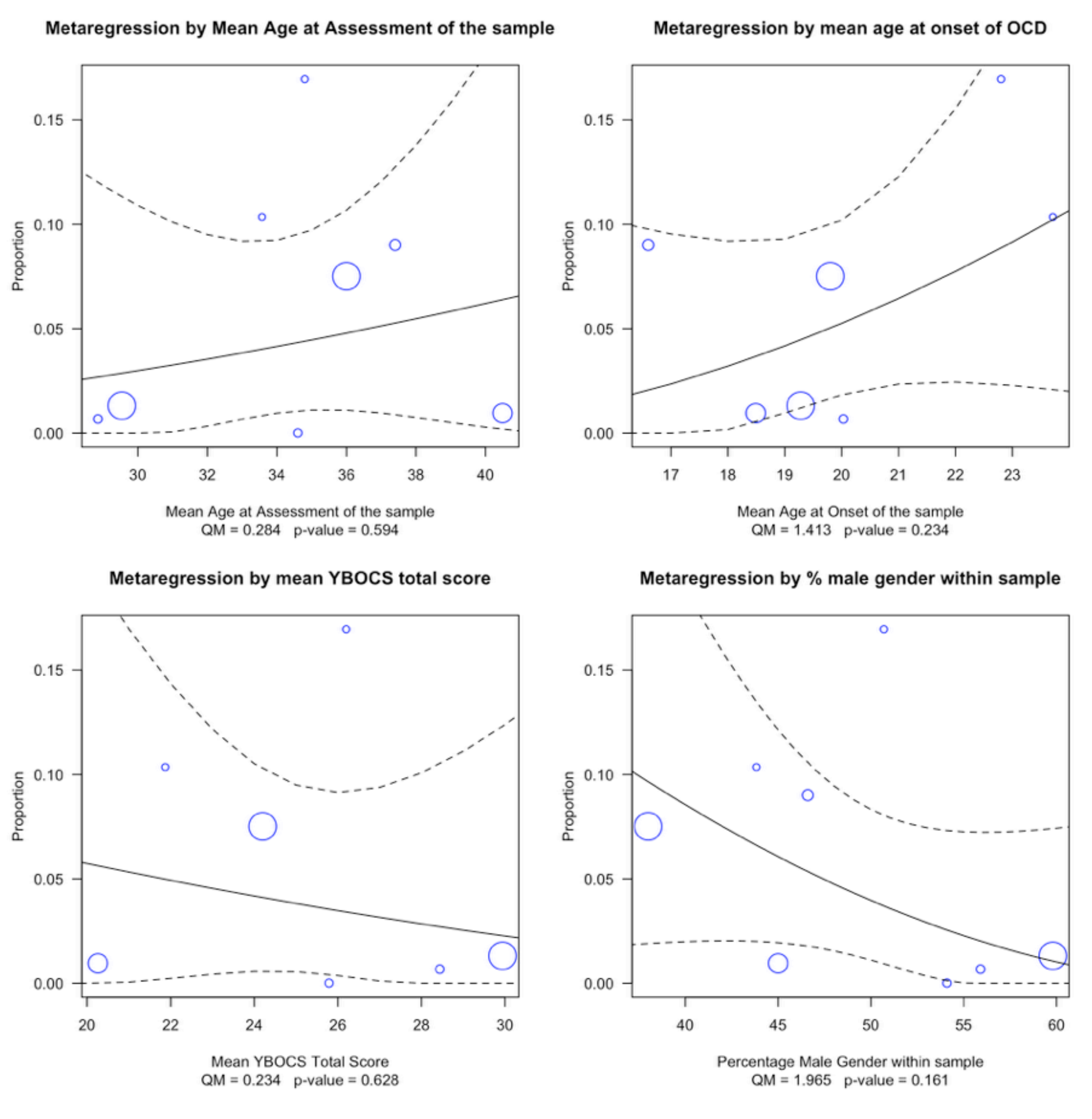

Suppl Figure 3.36 Meta-regression plots for Anxious-avoidant personality disorder

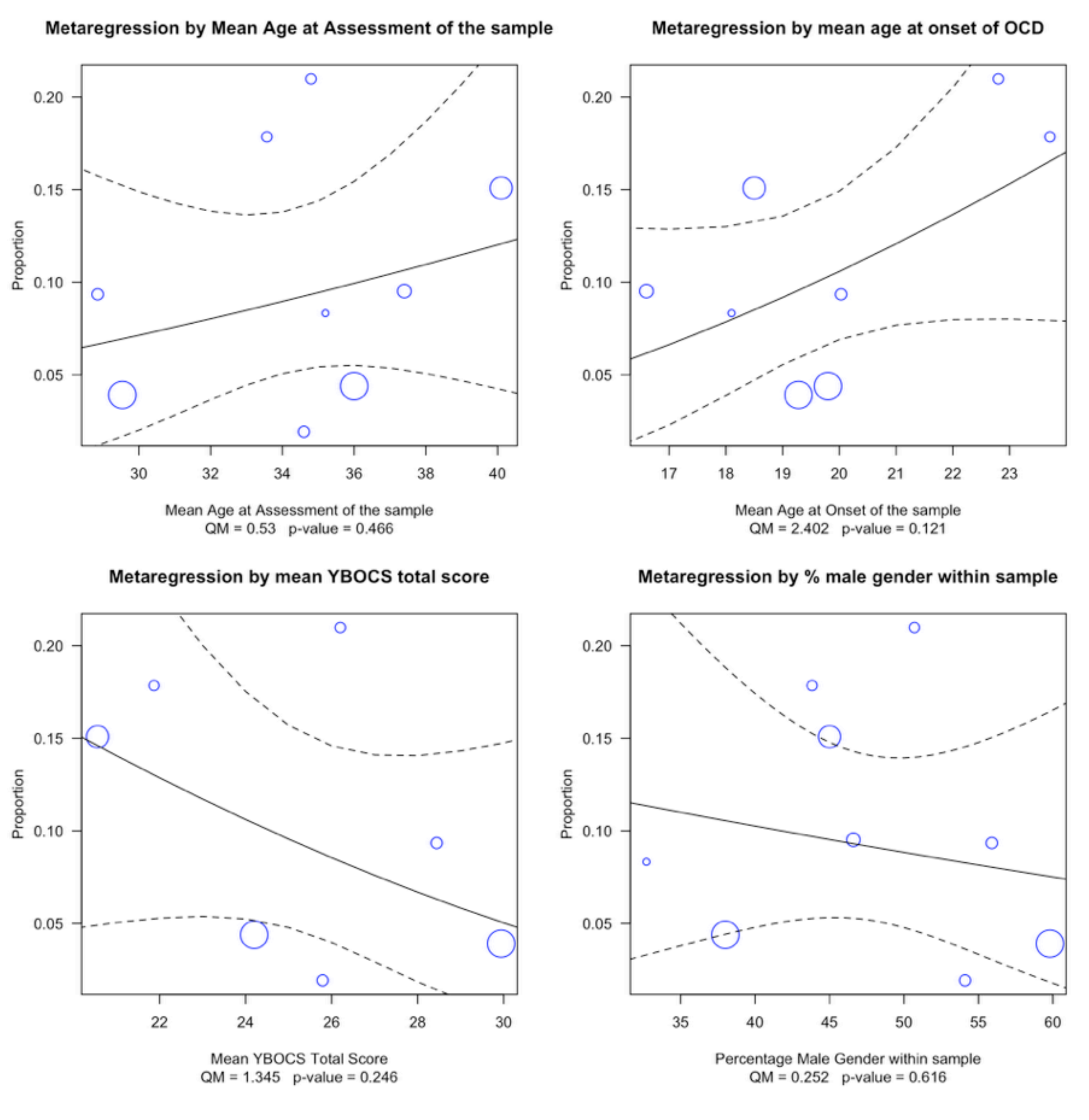

Supplement: Supplementary file 4 [file Data_Sheet_4.PDF]
