# Supplementary material for: Comorbidities in Obsessive-Compulsive Disorder Across the Lifespan: A Systematic Review and Meta-Analysis
Source: Front Psychiatry. 2021 Nov 11;12:703701. doi: 10.3389/fpsyt.2021.703701 (PMC8631971; doi:10.3389/fpsyt.2021.703701)
Supplement: Supplementary file 5 [file Data_Sheet_5.PDF]

Table 1: GRADE scoring for each comorbidity in the meta-analysis

	Comorbidity	Risk of bias	Inconsistency	Imprecision	Indirectness	Publication bias	GRADE score
1	Any psychiatric comorbidity	1	0	1	1	1	4
2	Any mood disorder	1	0	1	1	1	4
3	Major Depressive Disorder	1	0	1 (0 for paediatric)	1	1	4 (3 for paediatric)
4	Dysthymia	1	0	1	1	0	3
5	Bipolar Disorder	1	0	1	1	1	4
6	Schizophrenia/Any psychotic disorder	1	0	1	1	0	3
7	Any anxiety disorder	1	0	1	1	1	4
8	Generalised Anxiety Disorder	1	0	1	1	1	4
9	Social Anxiety Disorder	1	0	1	1	1	4
10	Panic Disorder	1	0	1 (0 for paediatric)	1	1	4 (3 for paediatric)
11	Agoraphobia	1	0	1	1	0	3
12	Simple/Specific Phobia	1	0	1	1	1	4
13	Somatic symptom disorder/Somatoform/Somatization disorder	1	0	1	1	1	4
14	Hypochondriasis/Illness anxiety disorder	1	0	1	1	1	4
15	Post-traumatic stress disorder (PTSD)	1	0	1	1	0	3
16	Any obsessive compulsive related (spectrum) disorders	1	0	0	1	1	3
17	Body dysmorphic disorder	1	0	0	1	1	3
18	Trichotillomania	1	0	0	1	1	3
19	Any tic disorder	1	0	1	1	1	4
20	Any eating disorder	1	0	1	1	0	3
21	Anorexia nervosa	1	0	1	1	1	4
22	Bulimia nervosa	1	1	1	1	1	5
23	Any substance use disorder	1	0	1	1	1	4
24	Autism spectrum disorder	1	1	1	1	1	5
25	Attention deficit hyperactivity disorder	1	0	1	1	1	4
26	Oppositional defiant disorder	1	0	0	1	1	3
27	Any personality disorder	1	0	1	1	1	4
28	Obsessive compulsive (Anankastic) personality disorder	1	0	1	1	0	3
29	Schizoid personality disorder	1	0	1	1	0	3
30	Schizotypal personality disorder	1	0	1	1	1	4

31	Borderline personality disorder	1	0	1	1	1	4
32	Histrionic personality disorder	1	0	1	1	1	4
33	Antisocial personality disorder	1	1	1	1	1	5
34	Narcissistic personality disorder	1	0	1	1	0	3
35	Dependent personality disorder	1	0	1	1	1	4
36	Anxious-avoidant personality disorder	1	0	1	1	1	4

Table 2: Operationalized criteria for GRADE

**GRADE criteria**	**Operationalized criteria**	**Explanation of GRADE scoring**	**GRADE Score assignment**
Risk of bias	1. Study design (cohort)<br><br>2. Representativeness of sample<br><br>3. Standardized assessments<br><br>4. Prevalence estimates considered covariates<br><br>5. Sensitivity analyses	Covered in selection criteria, most studies were cross-sectional, few were longitudinal in nature for the comorbidity assessed<br><br>Covered in selection criteria for studies – we only included studies that did not include/ exclude subjects for any comorbidity<br><br>We only included studies that diagnosed OCD and comorbidities using a standardized tool<br><br>Moderator (gender, age of onset, age at assessment, illness severity) analyses were performed for all the comorbidities studied<br><br>Use of leave-one-out analysis, influence and Baujat plots for identification of outliers was done as part of the statistical analysis.	Score of ‘1’ was given to all comorbidities
Inconsistency	1. Heterogeneity (I^2)<br><br>2. Subgroup analysis (by age)<br><br>3. Sample size variations	High heterogeneity (typically >90%) that was statistically significant led to a reduction in the GRADE score<br><br>This was done for all comorbidities based on adult versus paediatric age group<br><br>All included studies had a minimum sample size of 100. There was significant variation between studies for most comorbidities. This perhaps contributed to the high heterogeneity.	All comorbidities with statistically significant heterogeneity were scored ‘0’. Only ASD, Bulimia nervosa and ASPD had non-significant heterogeneity and were scored ‘1’.
Imprecision	95% confidence intervals	95% confidence intervals that wide enough to impact clinical implications at the upper and lower limit of intervals resulted in a reduction of the GRADE score	“Any OCRD”, “Trichotillomania”, “BDD” and “ODD” received a score of 0, along with Panic

			disorder and MDD in paediatric studies. All others were scored '1'.
Indirectness	Generalizability and applicability of findings	All the included studies recruited subjects from clinical populations, hence the findings may be considered generalizable to clinical populations	All received a score of '1'
Publication bias	Funnel plots<br>Egger's test<br>Begg's test	If either of Egger's or Begg's tests were significant, or asymmetry was evident on the Funnel plot, a score of '0' was assigned	Schizophrenia/any psychotic disorder, OCPD and narcissistic personality disorder were given a score of "0", based on Egger's/Begg's test; Dysthymia, Agoraphobia, PTSD, Any eating disorder, and Schizoid personality disorder were given a score of '0' based on the Funnel plots. All others were scored '1'.
